# Untargeted plasma metabolomic fingerprinting highlights several biomarkers for the diagnosis and prognosis of coronavirus disease 19

**DOI:** 10.3389/fmed.2022.995069

**Published:** 2022-09-29

**Authors:** Céline Occelli, Jean-Marie Guigonis, Sabine Lindenthal, Alexandre Cagnard, Fanny Graslin, Vesna Brglez, Barbara Seitz-Polski, Jean Dellamonica, Jacques Levraut, Thierry Pourcher

**Affiliations:** ^1^Transporter in Imaging and Radiotherapy in Oncology Laboratory (TIRO), Direction de la Recherche Fondamentale (DRF), Institut des Sciences du Vivant Frederic Joliot, Commissariat a l’Energie Atomique et aux Energies Alternatives (CEA), Université Côte d’Azur, School of Medicine, Nice, France; ^2^Department of Emergency, University Hospital, Nice, France; ^3^School of Medicine, Université Côte d’Azur, Nice, France; ^4^Unité de Recherche Clinique Côte d’Azur (UR2CA), Université Côte d’Azur, Nice, France; ^5^Department of Immunology, University Hospital, Nice, France; ^6^Medical Intensive Care Unit, University Hospital, Nice, France

**Keywords:** biomarkers, COVID-19, metabolomics (OMICS), diagnostic, prognostic

## Abstract

**Objectives:**

The COVID-19 pandemic has been a serious worldwide public health crisis since 2020 and is still challenging healthcare systems. New tools for the prognosis and diagnosis of COVID-19 patients remain important issues.

**Design:**

Here, we studied the metabolome of plasma samples of COVID-19 patients for the identification of prognosis biomarkers.

**Patients:**

Plasma samples of eighty-six SARS-CoV-2-infected subjects and 24 healthy controls were collected during the first peak of the COVID-19 pandemic in France in 2020.

**Main results:**

Plasma metabolome fingerprinting allowed the successful discrimination of healthy controls, mild SARS-CoV-2 subjects, and moderate and severe COVID-19 patients at hospital admission. We found a strong effect of SARS-CoV-2 infection on the plasma metabolome in mild cases. Our results revealed that plasma lipids and alterations in their saturation level are important biomarkers for the detection of the infection. We also identified deoxy-fructosyl-amino acids as new putative plasma biomarkers for SARS-CoV-2 infection and COVID-19 severity. Finally, our results highlight a key role for plasma levels of tryptophan and kynurenine in the symptoms of COVID-19 patients.

**Conclusion:**

Our results showed that plasma metabolome profiling is an efficient tool for the diagnosis and prognosis of SARS-CoV-2 infection.

## Introduction

Since 2020, we have been facing the global pandemic of coronavirus disease 19 (COVID-19), caused by severe acute respiratory syndrome coronavirus 2 (SARS-CoV-2). The main difficulties associated with COVID-19 are the lack of general specific symptoms, ranging from asymptomatic forms to acute respiratory distress syndrome (ARDS), its contagiousness and its morbidity and mortality rate (1). Despite rapid vaccine developments, COVID-19 is still a major health threat. COVID-19 vaccines are efficient tools for limiting the spread and severity of the disease but in many countries vaccination rates are still low for economic or ideological reasons. The development of efficient drugs for COVID-19 treatment (2) may improve the outcome of SARS-CoV-2 infection in the future. However, efficient global medical management of the COVID-19 pandemic requires a better understanding of the pathophysiological mechanisms of the disease. More tools are needed to help the clinician to establish a reliable prognostic and thus, to rapidly apply adequate treatment.

SARS-CoV-2 infections are commonly detected by RT-PCR and antigen testing on nose-throat swabs. RT-PCR tests are based on viral gene detection and reliable results can be obtained after several hours (3). Antigen tests based on viral antigen detection are more rapid with results available after approximately 20 min. The diagnosis of moderately and severely affected patients is now codified with combined clinical, biological and CT scan assessments. However, progress is still required in terms of prognostic and screening evaluation. Therefore, new approaches using molecular analyses are needed. Metabolomics, which had already been used to study several viral infections such as influenza (4) and EBOLA (5), was recently also used for studies with COVID-19 patients (6, 7). These metabolomic studies were mainly used with patients suffering moderate to severe symptoms with the aim to develop tools for screening severe patients and for predicting their outcome. The studied cohorts included few patients with mild or asymptomatic forms of COVID-19 (8, 9).

The aims of this study were to identify plasma metabolome signatures that enable the robust classification of asymptomatic, mildly, moderately, and severely affected COVID-19 patients, to predict the course of the patient’s medical condition, and to assess new insights in the underlying biological mechanisms of the disease.

## Materials and methods

### Patient selection and procedure

The analyses were conducted on a cohort of plasma samples previously used in the study of Ruetsch et al. (10). These blood samples were obtained from 86 SARS-CoV-2-infected and 24 healthy control volunteers. All samples were collected during the first peak of the COVID-19 pandemic in France in 2020. Patients were considered to be COVID-19 cases according to the WHO classification: presence of chilblain or CT scan characteristic of COVID-19 or two consecutive positive RT-PCR tests for SARS-CoV-2 virus or positive serological tests. To evaluate whether the metabolomic approach is suitable for discriminating subjects in terms of diagnosis and/or prognosis, patients were separated into three groups according to the severity of the infection: patients with mild symptoms of COVID-19 (chilblains or flu-like symptoms not requiring hospital monitoring), patients with moderate symptoms (patients hospitalized in infectious disease units) and patients with a severe form of COVID-19 (requiring hospitalization in an intensive care unit). Plasma samples of the Moderate and Severe groups were obtained from patients at hospital admission. Non-infected subjects with a negative serological test for SARS-CoV-2 comprised the control group. An informed consent was obtained for all participants. The study protocol conformed to the ethical guidelines of the 1975 Declaration of Helsinki and was approved by the appropriate institutional review committee (NCT04355351).

### Collection and processing of plasma samples for omic analyses

A volume of 100 μL plasma was mixed with 100 μL H_2_O (HPLC grade, Merck Millipore, USA), and 600 μL methanol (HPLC grade, Merck Millipore, USA) was added. Samples were vortexed and incubated overnight at −20°C for protein precipitation. After centrifugation (13.000 × g, 15 min, 4°C), the supernatant was removed, dried using a SpeedVAC concentrator (SVC100H, SAVANT, Thermo Fisher Scientific, Illkirch, France), resuspended in 80 μL 20:80 acetonitrile-H_2_O mixture (HPLC grade, Merck Millipore) and stored at −20°C until use for metabolomic analyses.

### Metabolomic analyses

Chromatographic analyses were performed with the DIONEX Ultimate 3000 HPLC system coupled to a chromatographic column (Phenomenex Synergi 4 u Hydro-RP 80A 250 × 3.0 mm) set at 40°C and a flow rate of 0.9 mL/min. Gradients of mobile phases (mobile phase A: 0.1% formic acid in water and mobile phase B: 0.1% formic acid in acetonitrile) were performed over a total of 25 min. MS analyses were carried out on a Thermo Fisher Scientific Exactive Plus Benchtop Orbitrap mass spectrometer. The heated electrospray ionization source (HESI II) was used in both positive and negative ion modes. The instrument was operated in full scan mode from m/z 60 to m/z 900. Data post-treatment was performed using the MZmine2 version 2.39^1^ (11). Typical Total Ion Chromatograms for positive and negative modes are illustrated in Supplementary Data 1C. Gap filling was performed using the “Same RT and m/z range gap filler” method of the MZmine software. The m/z tolerance was set up at 0.001 m/z or 10.0 ppm. Metabolites were putatively annotated [corresponding to level 2 of the Metabolomics Standard Initiative (MSI)]^2^ using the Human Metabolome Database version 5.0^3^ (12). Only ions identified as [M + H]^+^ adducts in the positive mode and [M-H]^–^ adducts in the negative mode and ions found in all the samples after gap filling were included. Metabolites identified as drugs or exogenous compounds were omitted. Full data sets (raw data) are available upon request.

### Statistical analyses

**FIGURE 1:**
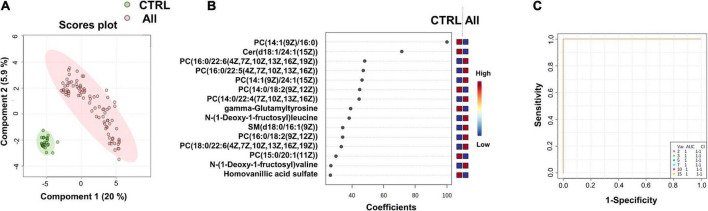
Identification of putative biomarkers of SARS-CoV-2 infection. PLS-DA analyses based on untargeted plasma metabolomic profiling discriminated healthy control subjects (CTRL) from all SARS-CoV-2 infected subjects (All, including mild, moderate and severe cases). Green and red dots represent CTRL and SARS-CoV-2 infected subjects, respectively. The score plot is represented with a confidence ellipse of 95% **(A)**. Loading plots of the top 15 features (metabolites) selected on the average of the first five components of the PLS-DA model. Identified metabolites were validated by MS2 analyses and are shown as chemical names **(B)**. Pairwise comparisons of areas under multivariate ROC curves (AUC) from each prognosis predictor **(C)**.

**FIGURE 2:**
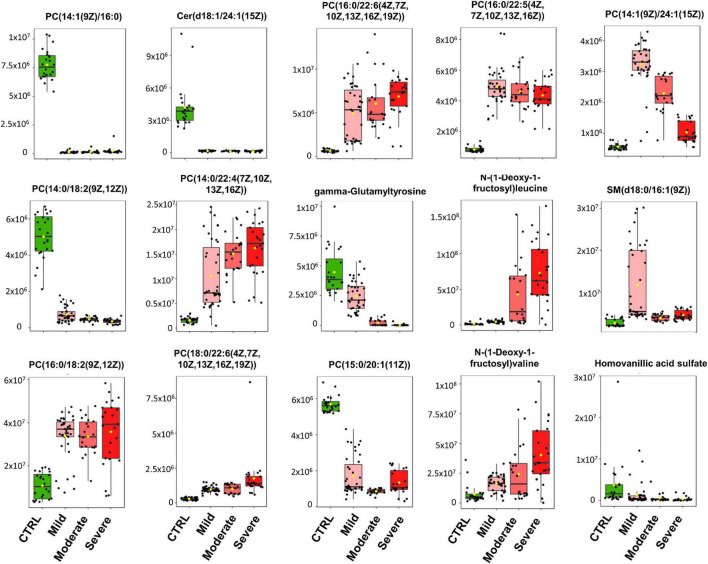
Heatmap of the top 15 metabolites of SARS-CoV-2 infection. Metabolites were clustered using the ward method on *t*-test and ANOVA values. The molecular structures of the lipids are given.

**FIGURE 3:**
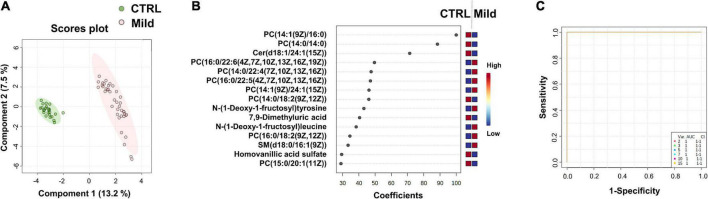
Identification of putative biomarkers of mild SARS-CoV-2 infected subjects compared to healthy control subjects. PLS-DA analysis based on untargeted plasma metabolomic profiling discriminated healthy control subjects (CTRL) from mild SARS-CoV-2-infected cases (Mild). Green and red dots represent CTRL and mild SARS-CoV-2-infected subjects, respectively. The score plot is represented with a confidence ellipse of 95% **(A)**. Loading plots of the top 15 features (metabolites) selected on the average of the first five components of the PLS-DA model. Identified features were MS2 validated and are shown as chemical names **(B)**. Pairwise comparisons of areas under multivariate ROC curve (AUC) from each prognosis predictor **(C)**.

**FIGURE 4:**
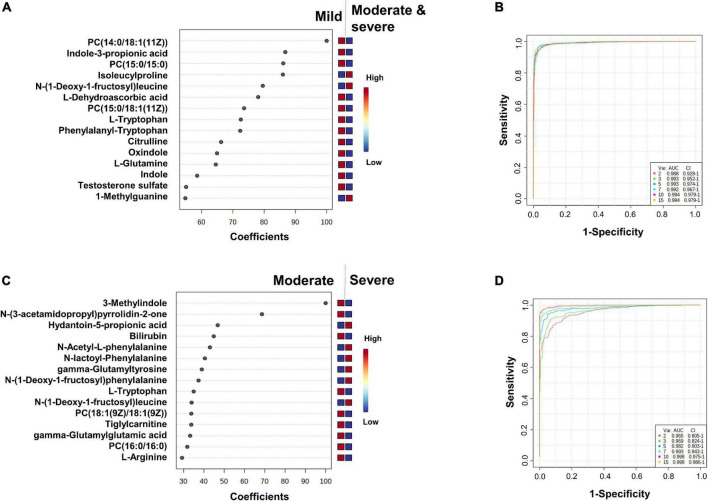
Identification of the putative biomarkers of moderate and severe COVID-19 patients compared to mild SARS-CoV-2 infected subjects **(A,B)** and severe compared to moderate COVID-19 patients **(C,D)**. PLS-DA analyses are shown in Supplementary Figure 2A and Figure 3A. Loading plots of the top 15 metabolites selected on the average of the first five components of the PLS-DA model. Identified features were MS2 validated and are shown as chemical names **(A,C)**. Pairwise comparisons of areas under multivariate ROC curve (AUC) from each prognosis predictor **(B,D)**.

Statistical analyses of the untargeted metabolomic studies were processed using statistical analysis modules (one factor) proposed by MetaboAnalyst 5.0^4^ (12). For each comparison, all samples of the data sets were median normalized and peak intensities were Log transformed. Using Partial Least Squares-Discriminant Analysis (PLS-DA), we analyzed loadings (average) of features selected by the PLS-DA model to identify discriminative metabolites. PLS-DA is considered particularly effective for biological systems to select discriminative features. The data identities of the top features shown in Figures 1–4, 6 were verified by the comparison of the obtained MS2 spectra with those of online data banks (Human Metabolome, MassBank Europe).^5^ MS2 matches are illustrated in Supplementary Data 1D, 2C, 3C, 4C. However, most of MS2 spectra that we found in the HMDB were predicted spectra. Therefore, MS2 matches are not corresponding to the level 1 of the MSI. Statistical tests to measure the association between major metabolites and clinical outcome such as Receiver-Operating Characteristic curves (ROC) were performed using the biomarker analysis module of MetaboAnalyst. The datasets of the four comparisons (see below) of the different groups of this study are given in the Supplementary Data 1A–4A. For each comparison, the data are ranked according to the PLS-DA average score (as per the loading plots in Figures 1B, 3B, 4A,C). Values and graphs are illustrated in the Supplementary Data 1B–4B. Several identified ions are putative biomarkers. To simplify the study, we mainly describe and discuss the top 15 metabolites from the PLS models.

**FIGURE 5:**
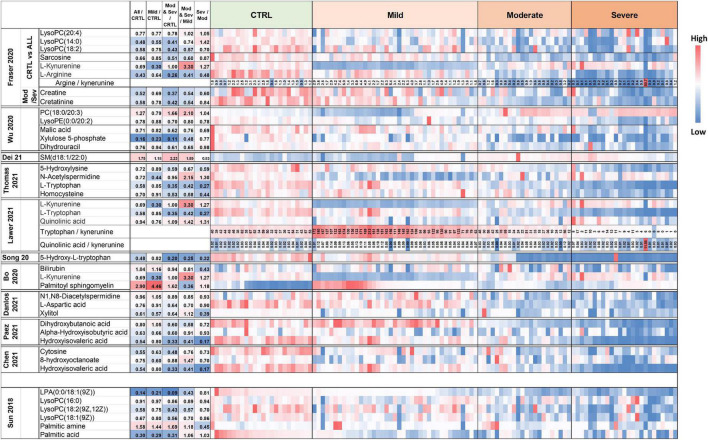
Heatmap of plasma levels of published putative biomarkers of SARS-CoV-2 infection and COVID-19 severity. The values are available in Supplementary Data 5–13.

**FIGURE 6:**
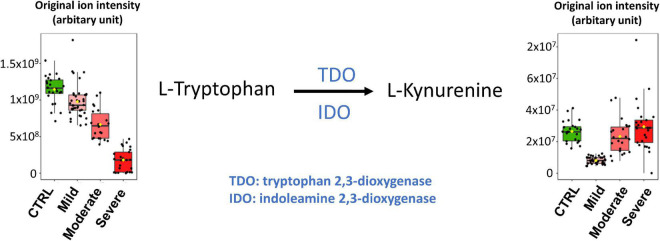
Venn diagram of significantly altered pathways in samples from healthy control subjects compared to SARS-CoV-2 infected subjects (CTRL vs. All), healthy control subjects compared to mild SARS-CoV-2 infected subjects (CTRL vs. Mild) and mild SARS-CoV-2 infected subjects compared to moderate and severe COVID-19 patients (Mild vs. Moderate and Severe). A value of *p* < 0.05 was considered significant.

### Pathway analyses

Analyses of related pathways were processed using pathway analysis modules proposed by MetaboAnalyst 5.0 (see text footnote 4) (12). Venn diagrams were performed to calculate and draw the intersections of pathway lists using a webtool.^6^

## Results

### Group characteristics

In this study, plasma metabolomes from 110 RT-PCR-tested subjects were analyzed. Epidemiological and clinical data are reported in Supplementary Table 1. The group (named “Mild”) of patients with only mild symptoms comprised 39 subjects; the group (named “Moderate”) of patients with moderate symptoms comprised 22 subjects; and the group (named “Severe”) of patients with severe symptoms comprised 25 subjects. Plasma samples from 24 health-care workers with additional negative serological test results and no apparent symptoms constitute the healthy control group (named “CTRL”). The Mild COVID-19 patients did not suffer any respiratory syndrome, however, one patient was treated by oxygen therapy (see Supplementary Table 1). Aiming the identification of biomarkers for the prognosis of COVID-19 severity, plasma samples of the Moderate and Severe groups were obtained from patients at hospital admission for respiratory illness and COVID-19 diagnosis *via* a positive RT-PCR test result. These patients were then divided into the two subgroups, i.e., Moderate or Severe through a follow-up, with those whose medical condition progressed to acute respiratory distress being assigned to the Severe group. All samples of the healthy control donors with a negative serological test for SARS-CoV-2 were obtained at the beginning of 2020 and were therefore from subjects supposedly never exposed to SARS-CoV-2 infection.

Gender (*p* = 0.004), mean age (*p* < 0.001), presence of cardiovascular history (*p* < 0.0001), diabetes (*p* = 0.0003), and cancer history (*p* = 0.04) were statistically different between the groups.

### Comparison of untargeted plasma metabolomic fingerprintings of healthy controls (CTRL) and severe acute respiratory syndrome coronavirus 2-infected (mild, moderate, and severe groups) subjects revealed that important metabolome changes occur in all infected patients (All)

The plasma metabolomes of non-infected control subjects were compared to those of the infected volunteers. After data post-treatment of the LC-MS analyses (see section “Materials and methods”), 605 metabolites were selected from the ions (m/z) obtained in both, positive and negative electrospray ionization modes. Metabolomics data (ranked according to the loading, see below) are available in Supplementary Data 1A.

We performed supervised multivariate analyses [Partial Least Squares-Discriminant Analysis (PLS-DA)] on these data using the MetaboAnalyst website. As illustrated in the score plot of Figure 1A, the plasma metabolomes of the control group (CTRL) could clearly be discriminated from those of the SARS-CoV-2-infected groups (All). The diagnostic performance of the model was evaluated by cross-validation. High accuracy values (1.0) for the PLS-DA model were obtained with 2 or more components. The most relevant model was obtained with five components (Q2 value of 0.99, for details see Supplementary Data 1B). Figure 1B shows the loading plots of the top 15 features ranked according to their overall coefficient scores of the PLS-DA model. This feature top list of 15 metabolites with highlighted lipids as the main contributors of the PLS-DA model.

As illustrated by the loading plots (Figure 1B) and the heatmap of these top 15 features (Figure 2), the levels of saturated lipids were lower in the plasma of SARS-CoV-2-infected subjects when compared to the control samples, whereas the levels of unsaturated lipids were higher. In addition, two N-(1-deoxy-1-fructosyl) amino acids (leucine and valine) were found among the top 15 features. The levels of these two amino acids were higher in the plasma of COVID-19 infected subjects compared to the control samples (Figures 1B, 2). Figure 1C shows that the area under the combined Receiving Operating Characteristic curve (ROC) for the prediction of SARS-CoV-2 infection of the top 15 features identified by the PSL-DA model that reached the score 1. This score remained unchanged even when considering only two metabolites from this top list [AUC = 1, CI 95% (1–1)].

### Comparison of untargeted plasma metabolomic fingerprintings of the control group samples and mild group samples revealed important differences

The main goal of this comparison was to determine if a metabolomic signature could be identified and used for the diagnosis of mild forms of COVID-19 infection. After post-treatment of the LC-MS/MS analyses data, 574 metabolites were selected (Supplementary Data 2A).

The score plot in Figure 3A shows the accuracy of the plasma-metabolome PLS-DA model to differentiate between the control and the mild group of SARS-CoV-2-infected patients. High accuracy values (1.0) of the PLS-DA model are obtained with 1 or more components. The highest scores were obtained with five components (Q2 value of 0.99) (details in Supplementary Data 2B). The top 15 metabolites identified by the PLS-DA model are shown in Figure 3B and in the heatmap of Supplementary Figure 1. Similar to the CTRL vs. All analysis, the metabolic profile shows variations in the levels of several lipids and two N-(1-deoxy-1-fructosyl) amino acids [N-(1-deoxy-1-fructosyl)tyrosine and N-(1-deoxy-1-fructosyl)leucine]. As expected, most metabolites of the top lists of the two comparisons were identical (see also CTRL vs. All in Figure 1B). Three metabolites of the CTRL vs. All top list, i.e., N-(1-deoxy-1-fructosyl)valine, the phosphatidylcholine PC[18:0/22:6(4Z,7Z,10Z,13Z,16Z,19Z)] and gamma-glutamyltyrosine were not found in the CTRL vs. Mild top list, which instead included 7,9-dimethyluric acid, PC (14:0/14:0) and N-(1-deoxy-1-fructosyl)tyrosine. As found for saturated lipids of the CTRL vs. All top list, the levels of PC (14:0/14:0) were significantly lower in the plasma of SARS-CoV-2-infected patients with mild symptoms compared to samples from the healthy control subjects. Similar to the findings of the amino acids of the CTRL vs. All top list, N-(1-deoxy-1-fructosyl)tyrosine levels were higher in samples of the mild group compared to those of the control group. Figure 3C shows the areas under the ROC curve to predict the presence of SARS-CoV-2 infection in asymptomatic subjects or patients with mild symptoms. The AUC values are 1 [95% CI (1–1)] when using two or more metabolites from the top list.

### Comparison of untargeted plasma metabolomic fingerprintings from subjects with mild symptoms (mild) with those of subjects of the moderate and severe group (moderate and severe)

This comparison aimed to determine if metabolomic signatures could be identified and used for the diagnosis and prognosis of moderate and severe forms of COVID-19. After post-treatment of the LC-MS/MS analyses data, 547 metabolites were selected (Supplementary Data 3A). The most relevant plasma metabolome PLS-DA model for the robust differentiation of samples of the mild group from those of the moderate and severe groups was obtained with five components (AUC = 0.99; Q2 value of 0.86) (see score plot in Supplementary Figure 2A and details in Supplementary Data 3B).

The top 15 metabolites identified by the PLS-DA model are shown in Figure 4A (heatmap in Supplementary Figure 2B). We observed that the levels of unsaturated lipids were lower in the plasmas of the moderate and severe group compared to those of the mild group. The N-(1-deoxy-1-fructosyl) leucine level was found to be higher in the plasmas of the moderate and severe group compared to those of the mild group. Indole, oxindole, indole-3-propionic acid, 3-methylindole levels were lower in the plasmas of the moderate and severe group compared to those of the mild group, as well as the L-dehydroascorbic acid, phenylalanyl-tryptophan, and citrulline levels. The areas under the ROC curve to predict the moderate or severe forms in SARS-CoV-2-infected patients are shown in Figure 4B. The AUC value is 0.988 [95% CI (0.928–1)] when using only two metabolites from the top list and the AUC values are 0.994 [95% CI (0.979–1)] when using the 10 or 15 top metabolites.

### Comparison of untargeted metabolomic fingerprintings of plasma from subjects with moderate symptoms (moderate) with those of subjects with severe symptoms (severe)

This comparison aimed to determine if metabolomic signatures could be used for the diagnosis and prognosis of severe forms of COVID-19. After post-treatment of the LC-MS/MS analyses data, 527 metabolites were selected (Supplementary Data 4A). The most relevant plasma-metabolome PLS-DA model to discriminate the moderate group from the severely affected patient group was obtained with four components (AUC = 0.94; Q2 value of 0.72) (score plot in Supplementary Figure 3A and details in Supplementary Data 4B). The top 15 metabolites identified by the PLS-DA model are shown in Figure 4C (heatmap in Supplementary Figure 3B).

We observed that the levels of two saturated lipids [PC (18:1(9Z)/18:1(9Z)) and PC (16:0/16:0)] were lower in the plasmas of the severe group compared to those of the moderate group. The levels of the two amino acids, N-(1-deoxy-1-fructosyl)alanine and N-(1-deoxy-1-fructosyl)leucine were higher in the plasmas of the severe group compared to those of the moderate group. In contrast, 3-methylindole levels were lower in the plasmas of the severe group compared to those of the moderate group, as were L-tryptophan, tiglylcarnitine, gamma-glutamylglutamic acid and L-arginine levels. N-acetyl-L-phenylalanine, N-lactoyl-phenylalanine and gamma-glutamyltyrosine levels were higher in plasmas of the severe group. The areas under the ROC curve to predict plasma from patients with moderate or severe forms of COVID-19 are shown in Figure 4D. The AUC values are 0.965 [95% CI (0.805–1)] when using only two metabolites from the top list, and 0.998 [95% CI (0.986–1)] when using 15 metabolites.

### Comparisons of the pathway analyses of untargeted plasma metabolomic fingerprintings for CTRL vs. all, CTRL vs. mild, and mild vs. moderate and severe

Using the MetaboAnalyst website, we studied the pathways related to the three comparative analyses described above. Only significant pathways were considered (*p*-value < 0.05). All the results are shown in the Supplementary Data 5–10.

A Venn diagram showing the intersections of the pathway lists for three comparisons (CTRL vs. All, CTRL vs. Mild and Mild vs. Moderate and Severe) is illustrated in Supplementary Figure 4 shows the Venn diagram for all four comparisons including Moderate vs. Severe. The Venn diagram (Supplementary Figure 4) highlights the major effects of SARS-CoV-2 infection that were already detected in the plasma samples from COVID-19 patients with mild symptoms compared to that of healthy control subjects. The major pathways found to be altered in mild COVID-19 patients included amino acid biosynthetic pathways, and lipid and energy metabolism.

### Correlation analyses between plasma untargeted metabolomic fingerprintings and IL-6 levels

In a previous study with blood samples from the same patient cohort as those used for the present analyses, Ruetsch et al. (10) revealed a correlation between plasma IL6 levels and the patient outcome. As illustrated in Supplementary Figure 5, our results showed that amino acid (tryptophan, glutamine, and threonine) levels are inversely correlated to plasma IL6 levels (sample correlation coefficient values of −0.616, −0.585, and −0.0551, respectively). In addition, six unidentified metabolites appeared to be positively correlated to plasma IL6 levels (sample correlation coefficient values of 0.779, 0.739, 0.725, 0.721, 0.717, and 0.705) (Supplementary Figure 5).

## Discussion

This study aims to identify plasma metabolomic signatures for the diagnosis of SARS-CoV-2 infection and the prognosis of COVID-19 disease severity. The identification of new biomarkers may also offer unique insights into the physiopathology of the disease.

All blood samples from this study were obtained during the first wave of the SARS-CoV-2 infections in France in 2020. Therefore, all samples of the infected subjects were most probably primary infections and the healthy control subjects had never been exposed to the SARS-CoV-2 virus. No vaccine was available at that time. Plasma samples of COVID-19 patients with moderate and severe symptoms were collected at hospital admission. At that point, patients had not yet benefited from specific drug administration or respiratory treatments.

Our results showed that untargeted metabolomic profiling of plasma samples from healthy control and SARS-CoV-2-infected subjects enabled the identification of new biomarkers and new metabolomic signatures for the diagnosis and prognosis of COVID-19. In the following section, we discuss the potential biological origins of the observed changes in the plasma levels of the identified metabolites due to SARS-CoV-2 infection.

Healthy control subjects (CRTL, *n* = 25) were hospital health-care workers with negative serological test results and no apparent symptoms. Mild subjects (*n* = 39) with positive serological test were patients with chilblains or flu-like symptoms that did not require hospital monitoring. The subjects of these two groups had no underlying diseases or chronic conditions, so we were able to conduct the analysis without having to include additional interfering factors. Therefore, we assumed that the changes in the plasma metabolite levels observed in the comparison CRTL vs. Mild (and, to a lesser extend in CRTL vs. All) were mainly evoked by SARS-CoV-2 infection. The baseline characteristics of the Moderate COVID-19 patients were not significantly different from those of the Severe subjects. Furthermore, the number of samples was low, 22 Moderate cases and 25 Severe cases, respectively. Therefore, we could not form subgroups. However, we assumed that the changes in the plasma metabolite levels found in the comparison Moderate vs. Severe were mainly caused by the severity of COVID-19 symptoms. In contrast, the baseline characteristics of the subjects in the Mild group differed from those in the Moderate and Severe groups. Corrections for the patient’s baseline characteristics were not performed as this would require a larger number of subjects in each group and subgroup. Therefore, the results of this comparison should be treated with caution. Thus, we have them carefully discussed here, focusing mainly on metabolites that have already been described in other publications.

We found that metabolome fingerprinting of control subjects were significantly different from those of all SARS-CoV-2-infected subjects independent of the severity of the disease (CTRL vs. All, illustrated by the PLS-DA score plot and multivariate ROC curves in Figures 1A,C, respectively and CTRL vs. Mild, illustrated by the PLS-DA score plot and multivariate ROC curves in Figures 3A,C, respectively). For both comparisons CTRL vs. All and CTRL vs. Mild, we identified a top list of metabolites that allow one to assign each subject to a specific class, i.e., CTRL, ALL, or Mild (illustrated in Figures 1B, 2, 3B and Supplementary Figure 1). The most frequent metabolites of both lists were lipids. In the top metabolite lists of the PLS models of the comparisons between infected subjects suffering from the disease at different severities (Mild vs. Moderate and Severe and Moderate vs. Severe), the elevated lipids were not found (see loading plots in Figures 4A,C). This result shows that the alteration of lipid metabolism is an early event in the SARS-CoV-2 infection. Fraser and collaborators performed targeted LC-MS/MS analyses with plasma samples of COVID-19 patients (13) and report the presence of LysoPCs in the top metabolite list.

Figure 5 (top lines) shows the heat map of the peak intensities of some of these LysoPCs of our dataset. Although these lipids are not part of our top list, we also detected higher levels in plasma from infected subjects. Wu and collaborators (7) report variations of the plasma lipid concentrations in COVID-19 patients. We detected similar changes in the plasma concentration of two of these lipids in our dataset (see Figure 5 and Supplementary Figure 6). Dei Cas and collaborators report a correlation between lipid signatures and prognostic factors for SARS-CoV-2 infected subjects (14). Only one of the lipids described in this study was also identified in our dataset (variation illustrated in Figure 5). Thomas and collaborators describe changes in free fatty acids and acylcarnitine levels in the plasma of COVID-19 patients when compared to control subjects (15). In addition, Bruzzone et al. show altered regulation of lipoproteins in the plasma of COVID-19 patients, with a reduction in HDL size, an increase in LDL size, and an increase in the level of intermediate VLDL subclasses (16). Song and collaborators report that plasma levels of sphingomyelins (SMs) are enhanced, and GM3s and those of diacylglycerols (DAGs) are reduced in COVID-19 patients (17). Several other groups suggest that certain fatty acids, including arachidonic acid and other unsaturated fatty acids play a key role in the cytokine storm during the initial phase of the infection and later in the inactivation and inhibition of SARS-CoV-2 proliferation (18, 19). Altered plasma lipid levels were also found in recovered COVID-19 patients (20) and during COVID-19 progression as shown by longitudinal metabolomic analyses (21). It will be interesting to assess if the levels of the lipids identified in this study also remain altered in recovered patients.

We performed LC-MS/MS experiments for untargeted metabolomic analyses of polar but also of hydrophobic compounds leading to the identification of new lipid biomarkers for SARS-CoV-2 infection. Interestingly, the top lists of our classification models revealed that the majority of lipids with lower levels in the plasma of SARS-CoV-2 infected subjects, i.e., CTRL vs. All and CTRL vs. Mild were mainly saturated lipids (as illustrated in Figures 2, 5 and Supplementary Figures 1, 6). In contrast, the majority of lipids with higher levels in the plasma of SARS-CoV-2 infected patients vs. healthy control subjects were unsaturated lipids. This finding could be related to an increased level of oxidative stress in SARS-CoV-2 infected subjects as has been suggested by other metabolomic studies (14, 15). Lipid metabolic remodeling of the host cell is a key feature of virus infection (22) and has been described and discussed for SARS-CoV-2 infection in several reviews (18, 23, 24). Recent study also indicates that expression of Spike protein impairs lipid metabolism of the host cells (25). This remodeling could evoke most variations in the endogenous lipid levels that we reported here. But, we also found that 2 odd-chain PCs were putative biomarkers of the SARS-CoV-2 infection and severity of the disease: PC[15/0/20:1(11Z)] for the comparison of CRTL vs. Mild (Figure 3B) and PC[15:0/18:1(11Z)] for the comparison Mild vs. Moderate and Severe (Figure 4A). Odd-chain lipids are considered as exogenous lipids mainly from milk consumption. The presence of odd-chain lipids could be related to exogenous (ingested) compounds and, in this case, should be removed from datasets in metabolomics studies. But, PC[15/0/20:1(11Z)] was detected at lower level in plasma samples of Mild COVID-19 patients compared to CRTL subjects. These 2 groups have many similarities (age and sex). The subjects of these groups were healthy. It is difficult to guess that in the Mild subjects had mainly diet with low odd-chain lipids. In addition, low levels of PC[15/0/20:1(11Z)] was also detected in plasma samples of COVID-19 patients (Moderate and Severe). These results indicate that PC[15/0/20:1(11Z)] is a putative biomarker of the SARS-CoV-2 infection. More experiments are needed to confirm this finding. PC[15:0/18:1(11Z)] was also detected at lower level in plasma samples of Moderate and Severe COVID-19 patients compared to Mild subjects. For this comparison Mild vs. Moderate and Severe, baseline characteristics of the subjects are different (Supplementary Table 1). The Moderate and Severe patients had COVID-19 symptoms and were hospitalized. But plasma samples were collected at hospital admission. All the subjects are living in the same area and they could have a similar diet. However, we cannot exclude an effect of the baseline characteristics when Mild (and CRTL) subjects are compared to Moderate and Severe patients. Therefore, further experiments are required to confirm that PC[15:0/18:1(11Z)] is a biomarker of disease severity.

Our study showed that N-(1-deoxy-1-fructosyl)amino acids were also putative biomarkers for SARS-CoV-2 infection (see Supplementary Figure 6). Leucine, valine and tyrosine derivatives were found in the top lists of our PLS models for the comparisons of CTRL vs. Mild and CTRL vs. ALL. N-(1-deoxy-1-fructosyl)leucine was detected in the top list of all the PLS models of this study and N-(1-deoxy-1-fructosyl)phenylalanine, as well as other phenylalanine derivatives (N-acetyl-L-phenylalanine and N-lactoyl-phenylalanine), were found in the top list of the PLS model of the comparison Moderate vs. Severe. To our knowledge, N-(1-deoxy-1-fructosyl)amino acids are poorly described in the literature and further studies are needed to understand the altered plasma levels of these amino acids in SARS-CoV-2 infected subjects. As discussed for odd-chain lipids, the presence of N-(1-deoxy-1-fructosyl)amino acids could be related to exogenous (ingested) compounds and, in this case, should be removed from datasets in metabolomics studies. Here, we included the N-(1-deoxy-1-fructosyl)amino acids in our analyses because these compounds were identified in all subject classes allowing us to assume that if they were exogenous, they were ingested randomly by all volunteers. Four N-(1-deoxy-1-fructosyl)amino acids were detected at a higher level in plasma samples of mild COVID-19 patients compared to healthy control subjects and were even higher with increasing severity of the disease.

In the CTRL vs. Mild comparison, lower levels of gamma-glutamyltyrosine and homovanillic acid sulfate were found for samples of infected subjects (Figure 1B and Figure 2). The levels of these compounds decreased even further in samples from the moderate group and especially in those from the severe group (see heat map in Figure 2). Gamma-glutamyltyrosine is produced when protein digestion or protein catabolism is incomplete. Homovanillic acid is a putative marker of metabolic stress (26). The role of these compounds during SARS-CoV-2 infection is still unclear and remains to be investigated. Likewise, the levels of 7,9-dimethyluric acid, a methyl derivative of uric acid, were found to be lower in the plasma of infected subjects than in the plasma of healthy control subjects (Figure 3B and Supplementary Figure 1).

The plasma levels of the amino acids, glutamine, arginine, citrulline, and tryptophan and their derivatives, indole-3-propionic acid and phenylalanyl-tryptophan, were found to be lower in samples of the moderate and severe groups than in samples of the mild group (Figure 3A and Supplementary Figure 2) suggesting an effect of disease severity on amino acid metabolism. Indole and indole-3-propionic acid are microbial metabolites and the observed effects on these metabolite levels could result from COVID-19-associated digestive disorders. The effects on dehydroascorbic acid levels could be linked to oxidative stress.

For the comparison of the Mild vs. Moderate and Severe groups (Figure 4C and Supplementary Figure 3), the metabolite top list of the PLS model contained N-(3-acetamidopropyl)pyrrolidin-2-one, an intermediate of spermidine catabolism and bilirubin, a degradation product of heme. Bilirubin degradation products are also found by Shen et al. (6) in the sera of COVID-19 patients. Similar to findings by Fraser and collaborators (13), we detected lower levels of arginine in samples of the severe group when compared to those of the moderate group. Kynurenine is a potential biomarker for COVID-19 reported in several studies (6, 13, 27–30) and Fraser et al. propose the use of an arginine vs. kynurenine ratio to predict the severity of the COVID-19 disease.

In our study, kynurenine was not found among the top lists of 15 metabolites of our four PLS models. However, kynurenine occupied the 29th, 24th, and 60th rank of the PLS models of the comparisons CTRL vs. All, CTRL vs. Mild and Mild vs. Moderate and Severe, respectively (see Supplementary Data 1A–4A). Although kynurenine did not appear in our top lists, this metabolite could still be considered as an interesting biomarker of SARS-CoV-2 infection.

As illustrated in the heatmap in Figure 5 and the box plot in Figure 6, lower plasma kynurenine levels were found for mild COVID-19 patients when compared to healthy control subjects and to moderate and severe COVID-19 patients. Kynurenine is described as a suppressor of the tumor-induced immune response in cancer patients (31, 32). The lower levels of kynurenine in the plasma of patients with mild COVID-19 symptoms, when compared to CTRL, could stimulate the immune response. Consequently, SARS-COV-2 infection and symptoms are reduced. In contrast, kynurenine levels in the plasmas of moderate and severe COVID-19 patients are like those in healthy control subjects and may contribute to limit the “cytokine storm” induced by SARS-CoV-2 infection and thus prevent inflammation and the appearance of more severe symptoms. The kynurenine precursor is tryptophan, and two enzymes catalyze the transformation of tryptophan to kynurenine [see (33) for review], indoleamine-2,3-dioxygenase (IDO) and tryptophan-2,3-dioxygenase (TDO). The plasma levels of tryptophan were decreased in the mild group compared to CTRL, but, in contrast to kynurenine, tryptophan levels were lower in the moderate group and lowest in the severe group (Figure 6). Our tryptophan level data corroborate the findings of Thomas et al. (15) and Lawler et al. (27). The two groups also report altered plasma tryptophan levels in the plasma of COVID-19 patients. We found that the levels of tryptophan decreased with the severity of the symptoms. Our results also showed that plasma kynurenine levels are only decreased in mild COVID-19. Therefore, we propose that the lower levels of kynurenine in mild COVID-19 patients compared to healthy control subjects could be a consequence of lower tryptophan levels in mild COVID-19 patients without activation of IDO or TDO. In contrast, increased transformation of tryptophan to kynurenine should be evoked in moderate and severe COVID-19 patients. This transformation stimulation should restore kynurenine levels in the presence of lower tryptophan levels but also contribute to lower the tryptophan level. Higher IL-6 levels (as reported in plasma of moderate and severe COVID-19 patients, see Supplementary Figure 5) could be responsible for the activation of IDO (34). Therefore, we propose that IL-6 could mediate an induction of tryptophan to kynurenine transformation for these patients *via* IDO activation. The raised levels of pro-inflammatory cytokines evident in SARS-CoV-2 infected patients have a number of important consequences, including the induction of IDO and the conversion of tryptophan to kynurenine, which can then activate the aryl hydrocarbon receptor (AhR), thereby dysregulating the immune response, including suppressing natural killer cell activation (35). Pro-inflammatory cytokines also increase gut permeability and gut dysbiosis, leading heightened levels of circulating LPS and suppressed levels of the short-chain fatty acid, butyrate (35). The suppression of butyrate heightens histone acetylation-driven epigenetic regulation, thereby altering the nature of patterned gene expression, including as driven by kynurenine activation of the AhR (36). Given the clinical utility of melatonin in the management of SARS-CoV-2 infection severity and mortality (37), it is important to note that the conversion of tryptophan to kynurenine will suppress serotonin levels, and therefore serotonin as the necessary precursor for the induction of the melatonergic pathway in body cells, including immune cells (38). Given that melatonin production and autocrine effects are necessary to shift macrophages and microglia from an M1-like pro-inflammatory phenotype to an M2-like pro-phagocytic phenotype (39, 40), such alterations in tryptophan availability will significantly dysregulate the patterned immune response. The data in the current study may therefore link to wider systemic aspects of immune regulation involving the gut microbiome/permeability, AhR activation and suppressed mitochondrial melatonergic pathway activity, as recently proposed (41).

We also studied the pathways related to the obtained metabolome fingerprintings. As shown in Supplementary Data 5–8, we used MetaboAnalyst to identify significant pathways (*p*-values > 0.05) by performing two-class comparisons (45 pathways for the comparison CTRL vs. All; 31 pathways for CTRL vs. Mild; 42 pathways for Mild vs. Moderate and Severe; 22 pathways for Moderate vs. Severe). We then established Venn diagrams (see Supplementary Data 9, 10) to identify common pathways among the different comparisons (Supplementary Figure 4). The Venn diagram obtained for the three comparisons, CTRL vs. All, CTRL vs. Mild and Mild vs. Moderate and Severe, is given in Supplementary Data 10. It showed that the majority of pathways that were altered by SARS-CoV-2 infection were detected in samples from subjects with only mild symptoms. These pathways mainly belonged to lipid and amino acid metabolism (Supplementary Figure 4). These results clearly indicate that SARS-CoV-2 infection caused strong alterations of the plasma metabolome even though the infected subjects suffered only very mild symptoms. Further studies are now needed to obtain a better understanding of the physiopathology of the mild form of COVID-19 and possible side effects the virus infection. In this context, it will be interesting to elucidate the effect of COVID-19 vaccination on the plasma metabolome response to SARS-CoV-2 infection.

All samples of our cohort were collected in the hospital of Nice during the first COVID-19 wave of the pandemic. This means there was a restricted coverage area for sample collection but also guaranteed that all subjects that participated in the study (healthy controls and COVID-19 patients) were never exposed to SARS-CoV-2 infection or vaccination prior to sample collection. The limited number of samples of each class did not allow for both a training set and a validation set. However, as described above and illustrated in Figure 6, our results are supported by metabolomic analyses published by other groups.

Based on our studies and those of the other groups, several robust metabolome plasma biomarkers for COVID-19 diagnosis and prognosis have been identified. These plasma biomarkers could be used for targeted LC-MS/MS analyses which are fast, low-cost, and suitable for routine clinical testing. Metabolomic studies of other biological fluids such as urine (42) or exhaled breath (43) already gave interesting results and could also be very useful for rapid COVID-19 diagnosis and prognosis. However, further studies are now needed to identify biomarkers for the diagnosis of specific SARS-CoV-2 variants. It would also be beneficial to compare the plasma metabolome fingerprintings of SARS-CoV-2-infected subjects and patients infected by other viruses that evoke flu-like or COVID-like symptoms. In this context, Sun and collaborators studied the levels of serum fatty acids related to disease severity after infection with H7N9, an avian-origin influenza A virus that can lead to severe lung damage (44). As illustrated in Figure 6, and similar to the findings of Sun et al., the plasma levels of the identified biomarkers for H7N9 decrease with increasing COVID-19 severity. Here, aiming the identification of biomarkers for the prognosis of COVID-19 severity, we studied plasma samples of COVID-19 patients with moderate and severe symptoms that were collected at hospital admission. For better understanding of the physiopathology of COVID-19, further studies will focus on metabolome analyses of samples that were collected repeatedly over the whole period in which our patient cohort stayed at the hospital. Finally, this study will be complemented by metabolomic analyses on samples from subjects infected with other SARS-CoV-2 variants (delta, omicron, etc.) and with samples from infected subjects who were previously vaccinated.

In conclusion, our results showed that plasma metabolome profiling is an efficient tool for the diagnosis and prognosis of SARS-CoV-2 infection. We found that modifications in the plasma levels of lipids and amino acids were the main features of these metabolomic signatures. In particular, high levels of unsaturated lipids and fructosyl amino acids, and low levels of saturated lipids were identified in the plasma of infected subjects and serve as robust biomarkers for the diagnosis of SARS-CoV-2 infection and the prognosis of COVID-19 severity. Our results also show that major alterations of metabolite levels due to SARS-CoV-2 infection occurred primarily in the plasma of subjects with only mild symptoms and that the kynurenate pathways play a key role in the symptoms of COVID-19.

## Data availability statement

All datasets can be found in the Supplementary material.

## Ethics statement

The studies involving human participants were reviewed and approved by the Comite de Protection des Personnes Sud-Ouest et Outre-Mer 1. The patients/participants provided their written informed consent to participate in this study. Written informed consent was obtained from the individual(s) for the publication of any potentially identifiable images or data included in this article.

## Author contributions

CO, SL, JL, and TP designed the study. CO, VB, BS-P, and JD collected clinical data. CO, JL, and TP verified the underlying data. CO and FG prepared the samples. J-MG performed the mass spectrometry analyses. CO, AC, and TP performed post treatment of the raw data, statistical analysis, and pathway analysis. CO, SL, and TP analyzed and interpreted the results. CO and TP wrote the manuscript. SL revised critically the report for important intellectual content. All authors revised critically the report and gave final approval of the version for publication and agreed to be accountable for all aspects of the work in ensuring that questions related to the accuracy or integrity of any part of the work are appropriately investigated and resolved.

## Footnotes

1
http://mzmine.github.io/

2
https://github.com/MSI-Metabolomics-Standards-Initiative/CIMR

3
http://www.hmdb.ca

4
https://www.metaboanalyst.ca

5
https://massbank.eu/MassBank/

6
http://bioinformatics.psb.ugent.be/webtools/Venn/
