# Supplementary material for: Untargeted plasma metabolomic fingerprinting highlights several biomarkers for the diagnosis and prognosis of coronavirus disease 19
Source: Front Med (Lausanne). 2022 Sep 29;9:995069. doi: 10.3389/fmed.2022.995069 (PMC9556858; doi:10.3389/fmed.2022.995069)
Supplement: Supplementary file 2 [file Presentation_1.pptx]

## Slide 1
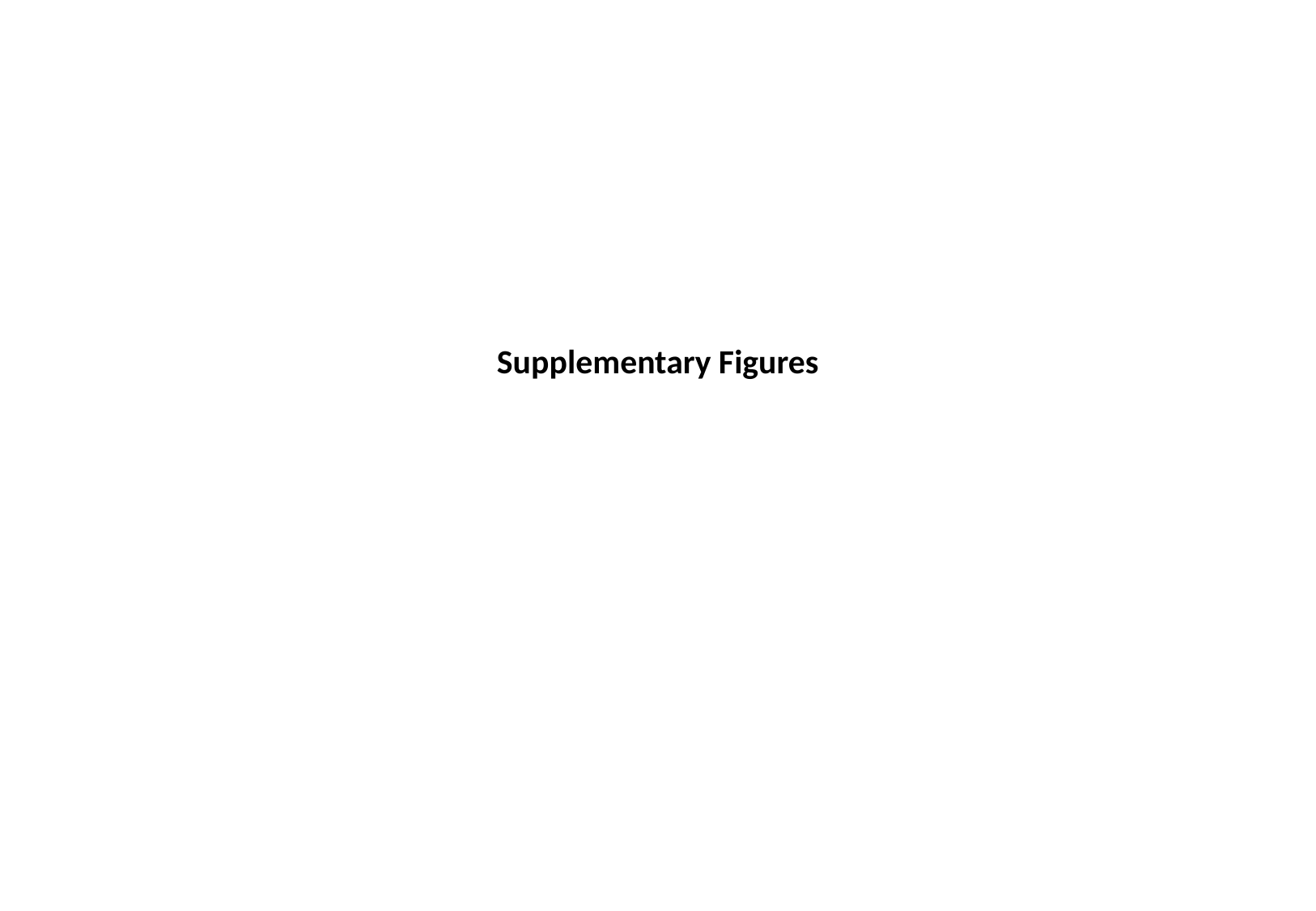

Supplementary Figures

## Slide 2
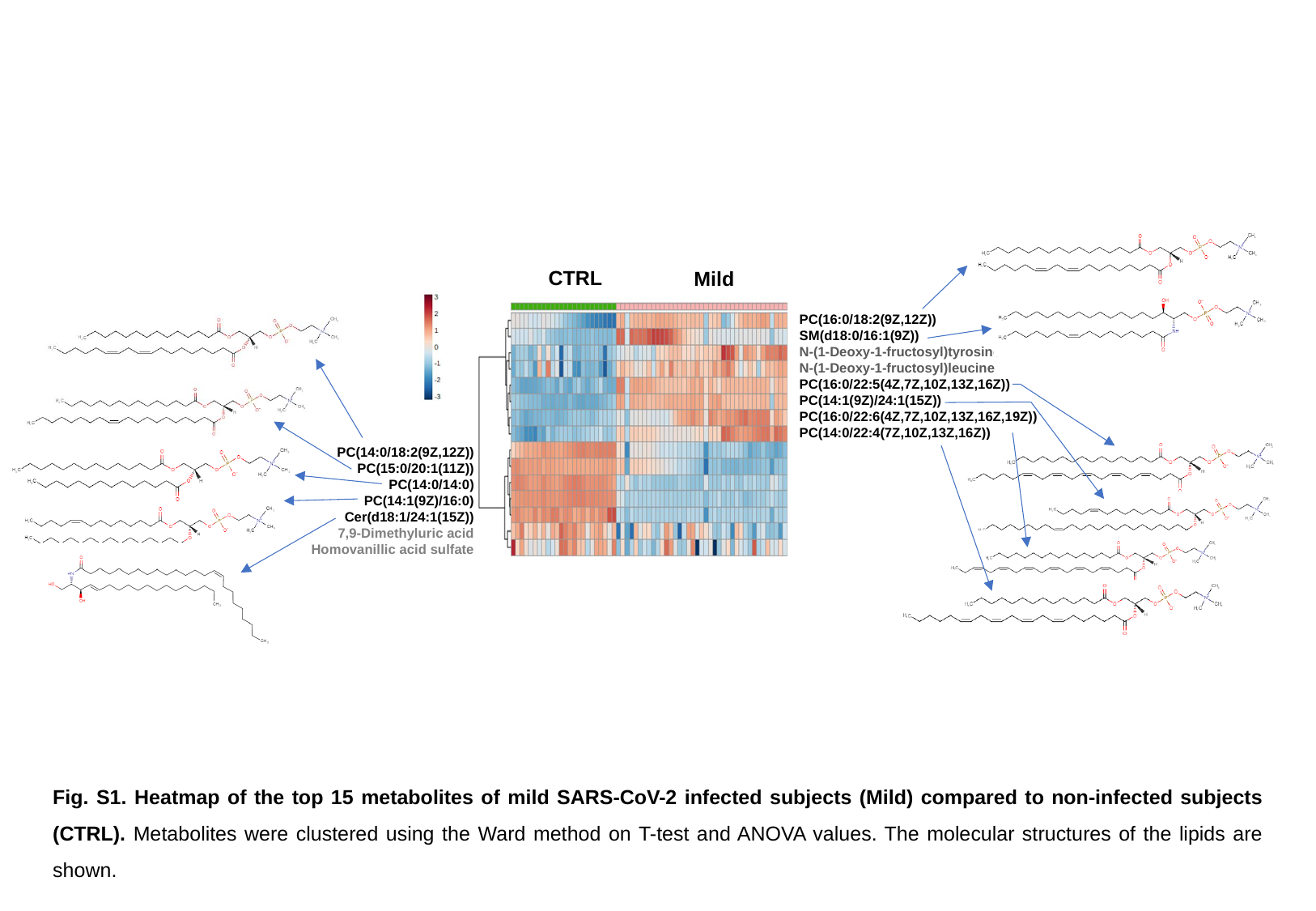

CTRL
Mild
PC(16:0/18:2(9Z,12Z))
SM(d18:0/16:1(9Z))
N-(1-Deoxy-1-fructosyl)tyrosine
N-(1-Deoxy-1-fructosyl)leucine
PC(16:0/22:5(4Z,7Z,10Z,13Z,16Z))
PC(14:1(9Z)/24:1(15Z))
PC(16:0/22:6(4Z,7Z,10Z,13Z,16Z,19Z))
PC(14:0/22:4(7Z,10Z,13Z,16Z))
PC(14:0/18:2(9Z,12Z))
PC(15:0/20:1(11Z))
PC(14:0/14:0)
PC(14:1(9Z)/16:0)
Cer(d18:1/24:1(15Z))
7,9-Dimethyluric acid
Homovanillic acid sulfate
Fig. S1. Heatmap of the top 15 metabolites of mild SARS-CoV-2 infected subjects (Mild) compared to non-infected subjects (CTRL). Metabolites were clustered using the Ward method on T-test and ANOVA values. The molecular structures of the lipids are shown.

## Slide 3
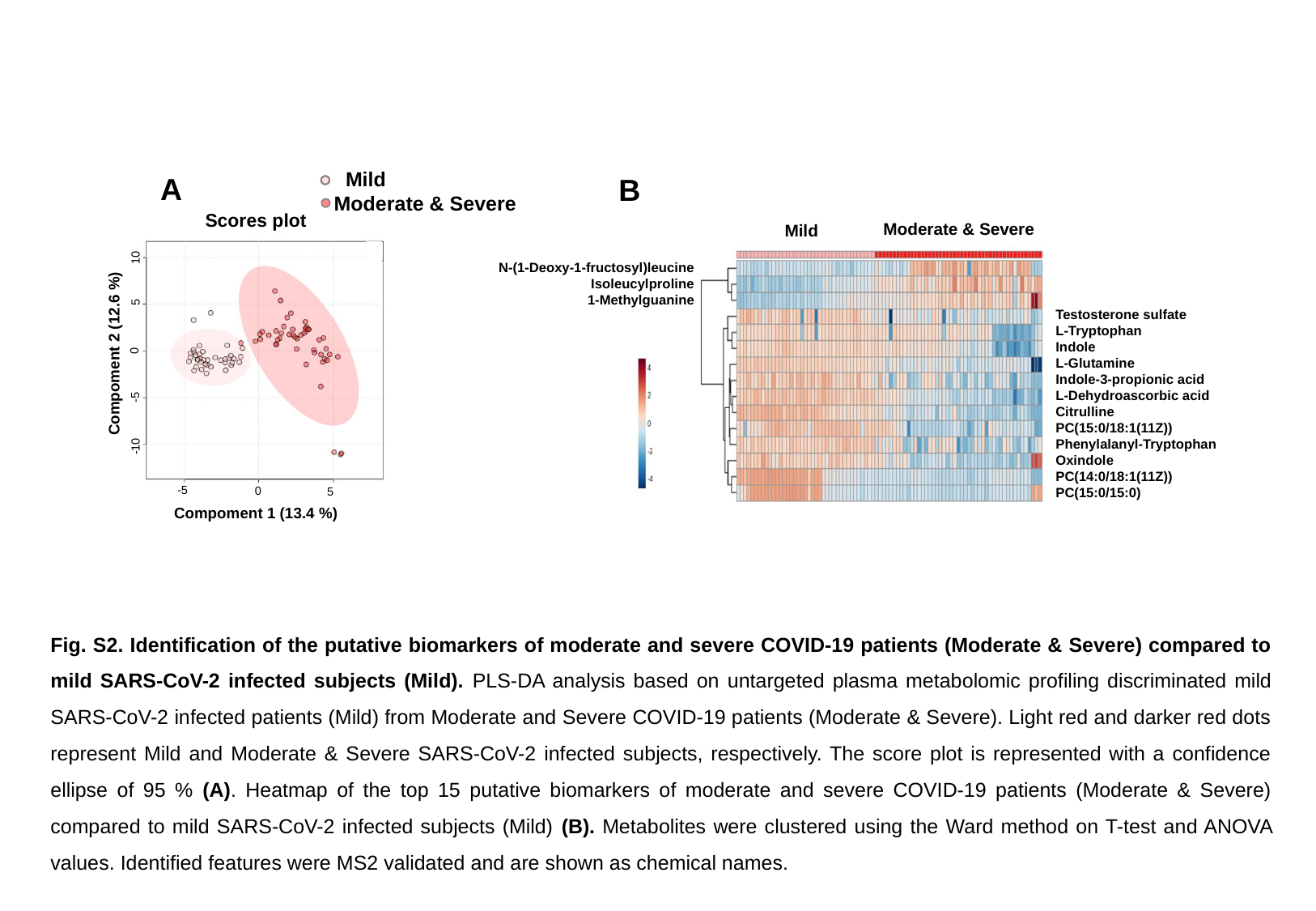

Mild
A
B
Moderate & Severe
Scores plot
Moderate & Severe
Mild
Testosterone sulfate
L-Tryptophan
Indole
L-Glutamine
Indole-3-propionic acid
L-Dehydroascorbic acid
Citrulline
PC(15:0/18:1(11Z))
Phenylalanyl-Tryptophan
Oxindole
PC(14:0/18:1(11Z))
PC(15:0/15:0)
10
N-(1-Deoxy-1-fructosyl)leucine
Isoleucylproline
1-Methylguanine
5
0
Compoment 2 (12.6 %)
-5
-10
-5
0
5
Compoment 1 (13.4 %)
Fig. S2. Identification of the putative biomarkers of moderate and severe COVID-19 patients (Moderate & Severe) compared to mild SARS-CoV-2 infected subjects (Mild). PLS-DA analysis based on untargeted plasma metabolomic profiling discriminated mild SARS-CoV-2 infected patients (Mild) from Moderate and Severe COVID-19 patients (Moderate & Severe). Light red and darker red dots represent Mild and Moderate & Severe SARS-CoV-2 infected subjects, respectively. The score plot is represented with a confidence ellipse of 95 % (A). Heatmap of the top 15 putative biomarkers of moderate and severe COVID-19 patients (Moderate & Severe) compared to mild SARS-CoV-2 infected subjects (Mild) (B). Metabolites were clustered using the Ward method on T-test and ANOVA values. Identified features were MS2 validated and are shown as chemical names.

## Slide 4
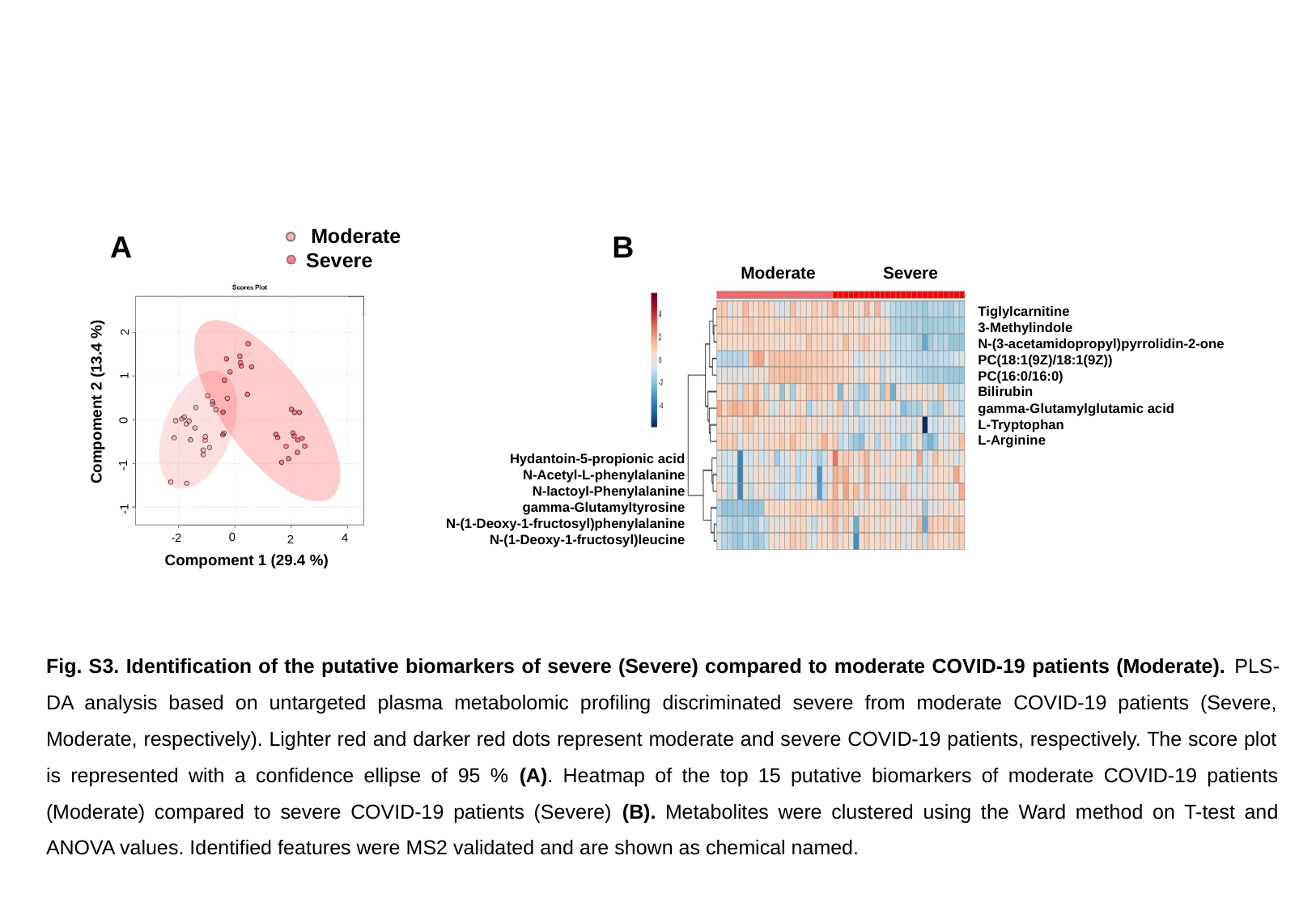

Moderate
A
B
Severe
Moderate
Severe
Tiglylcarnitine
3-Methylindole
N-(3-acetamidopropyl)pyrrolidin-2-one
PC(18:1(9Z)/18:1(9Z))
PC(16:0/16:0)
Bilirubin
gamma-Glutamylglutamic acid
L-Tryptophan
L-Arginine
2
1
Compoment 2 (13.4 %)
0
Hydantoin-5-propionic acid
N-Acetyl-L-phenylalanine
N-lactoyl-Phenylalanine
gamma-Glutamyltyrosine
N-(1-Deoxy-1-fructosyl)phenylalanine
N-(1-Deoxy-1-fructosyl)leucine
-1
-1
0
4
-2
2
Compoment 1 (29.4 %)
Fig. S3. Identification of the putative biomarkers of severe (Severe) compared to moderate COVID-19 patients (Moderate). PLS-DA analysis based on untargeted plasma metabolomic profiling discriminated severe from moderate COVID-19 patients (Severe, Moderate, respectively). Lighter red and darker red dots represent moderate and severe COVID-19 patients, respectively. The score plot is represented with a confidence ellipse of 95 % (A). Heatmap of the top 15 putative biomarkers of moderate COVID-19 patients (Moderate) compared to severe COVID-19 patients (Severe) (B). Metabolites were clustered using the Ward method on T-test and ANOVA values. Identified features were MS2 validated and are shown as chemical named.

## Slide 5
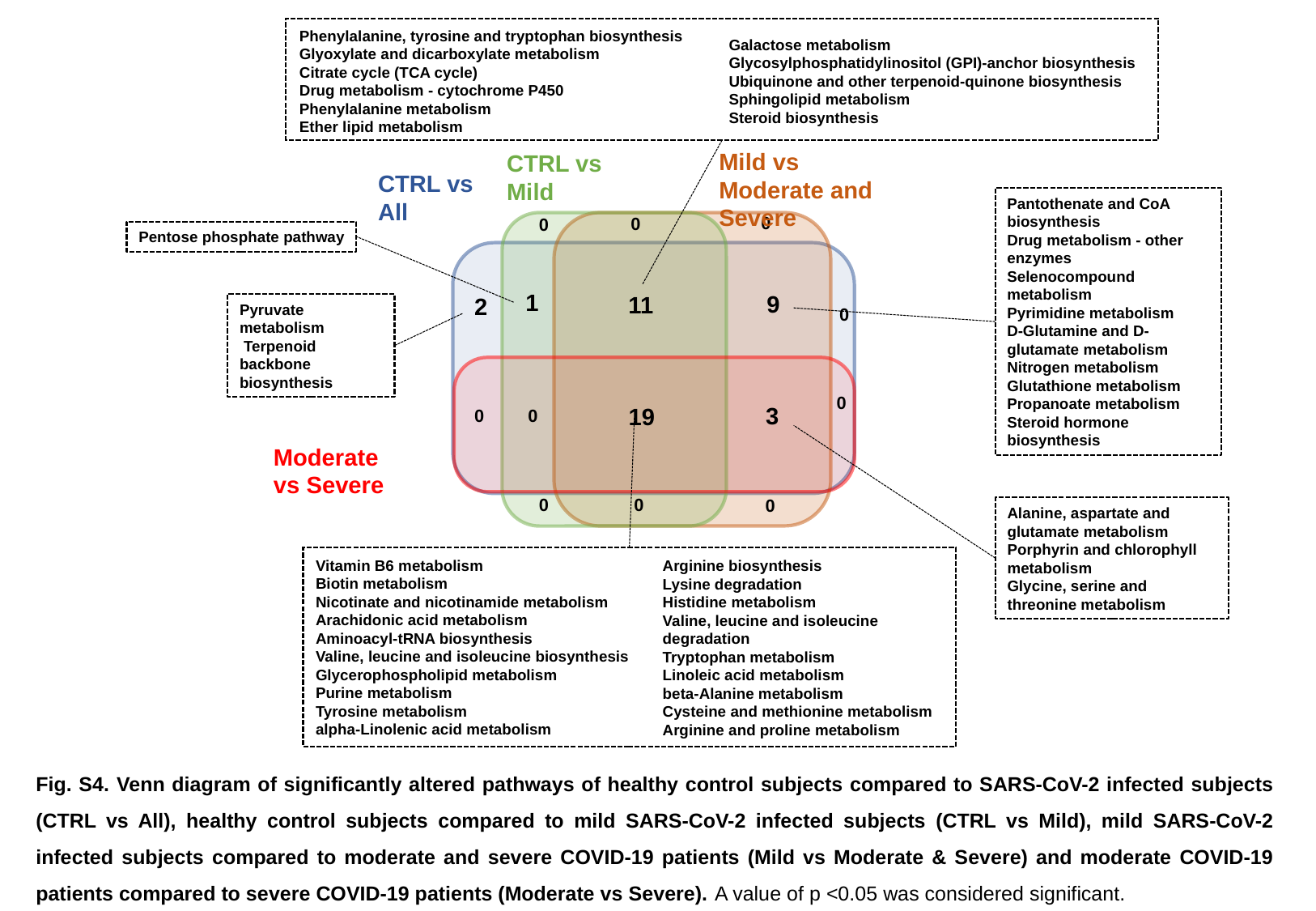

Phenylalanine, tyrosine and tryptophan biosynthesis Glyoxylate and dicarboxylate metabolism
Citrate cycle (TCA cycle)
Drug metabolism - cytochrome P450
Phenylalanine metabolism
Ether lipid metabolism
Galactose metabolism
Glycosylphosphatidylinositol (GPI)-anchor biosynthesis
Ubiquinone and other terpenoid-quinone biosynthesis
Sphingolipid metabolism
Steroid biosynthesis
Mild vs
Moderate and Severe
CTRL vs
Mild
CTRL vs
All
Pantothenate and CoA biosynthesis
Drug metabolism - other enzymes
Selenocompound metabolism
Pyrimidine metabolism
D-Glutamine and D-glutamate metabolism
Nitrogen metabolism
Glutathione metabolism
Propanoate metabolism
Steroid hormone biosynthesis
0
0
0
Pentose phosphate pathway
1
9
11
2
Pyruvate metabolism
 Terpenoid backbone biosynthesis
0
0
3
19
0
0
Moderate vs Severe
0
0
0
Alanine, aspartate and glutamate metabolism
Porphyrin and chlorophyll metabolism
Glycine, serine and threonine metabolism
Vitamin B6 metabolism
Biotin metabolism
Nicotinate and nicotinamide metabolism
Arachidonic acid metabolism
Aminoacyl-tRNA biosynthesis
Valine, leucine and isoleucine biosynthesis
Glycerophospholipid metabolism
Purine metabolism
Tyrosine metabolism
alpha-Linolenic acid metabolism
Arginine biosynthesis
Lysine degradation
Histidine metabolism
Valine, leucine and isoleucine degradation
Tryptophan metabolism
Linoleic acid metabolism
beta-Alanine metabolism
Cysteine and methionine metabolism
Arginine and proline metabolism
Fig. S4. Venn diagram of significantly altered pathways of healthy control subjects compared to SARS-CoV-2 infected subjects (CTRL vs All), healthy control subjects compared to mild SARS-CoV-2 infected subjects (CTRL vs Mild), mild SARS-CoV-2 infected subjects compared to moderate and severe COVID-19 patients (Mild vs Moderate & Severe) and moderate COVID-19 patients compared to severe COVID-19 patients (Moderate vs Severe). A value of p <0.05 was considered significant.

## Slide 6
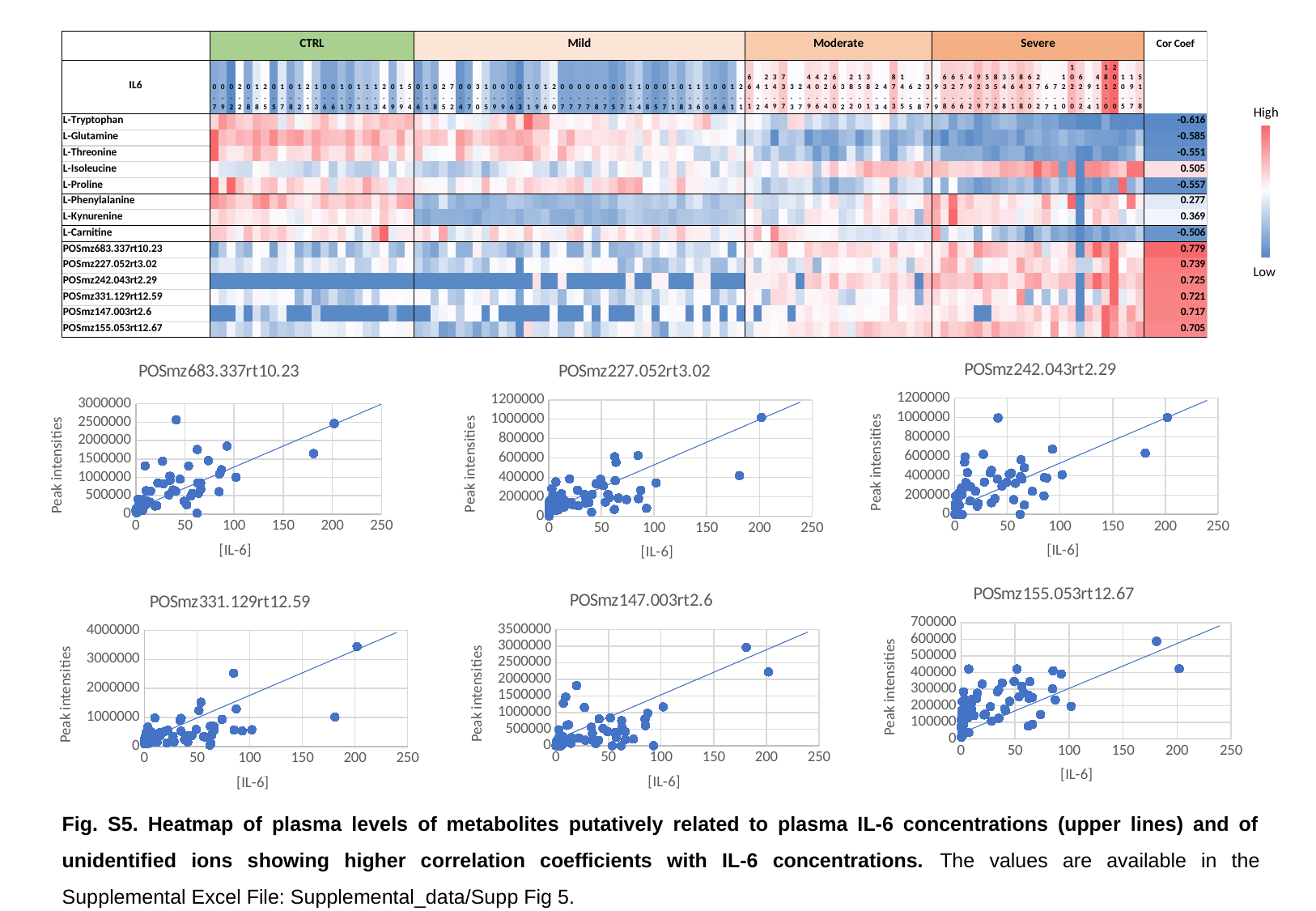

High
Low
### Chart:
| Category | POSmz242.043rt2.29 |
|---|---|
### Chart:
| Category | POSmz683.337rt10.23 |
|---|---|
### Chart:
| Category | POSmz227.052rt3.02 |
|---|---|
### Chart:
| Category | POSmz155.053rt12.67 |
|---|---|
### Chart:
| Category | POSmz147.003rt2.6 |
|---|---|
### Chart:
| Category | POSmz331.129rt12.59 |
|---|---|Fig. S5. Heatmap of plasma levels of metabolites putatively related to plasma IL-6 concentrations (upper lines) and of unidentified ions showing higher correlation coefficients with IL-6 concentrations. The values are available in the Supplemental Excel File: Supplemental_data/Supp Fig 5.

## Slide 7
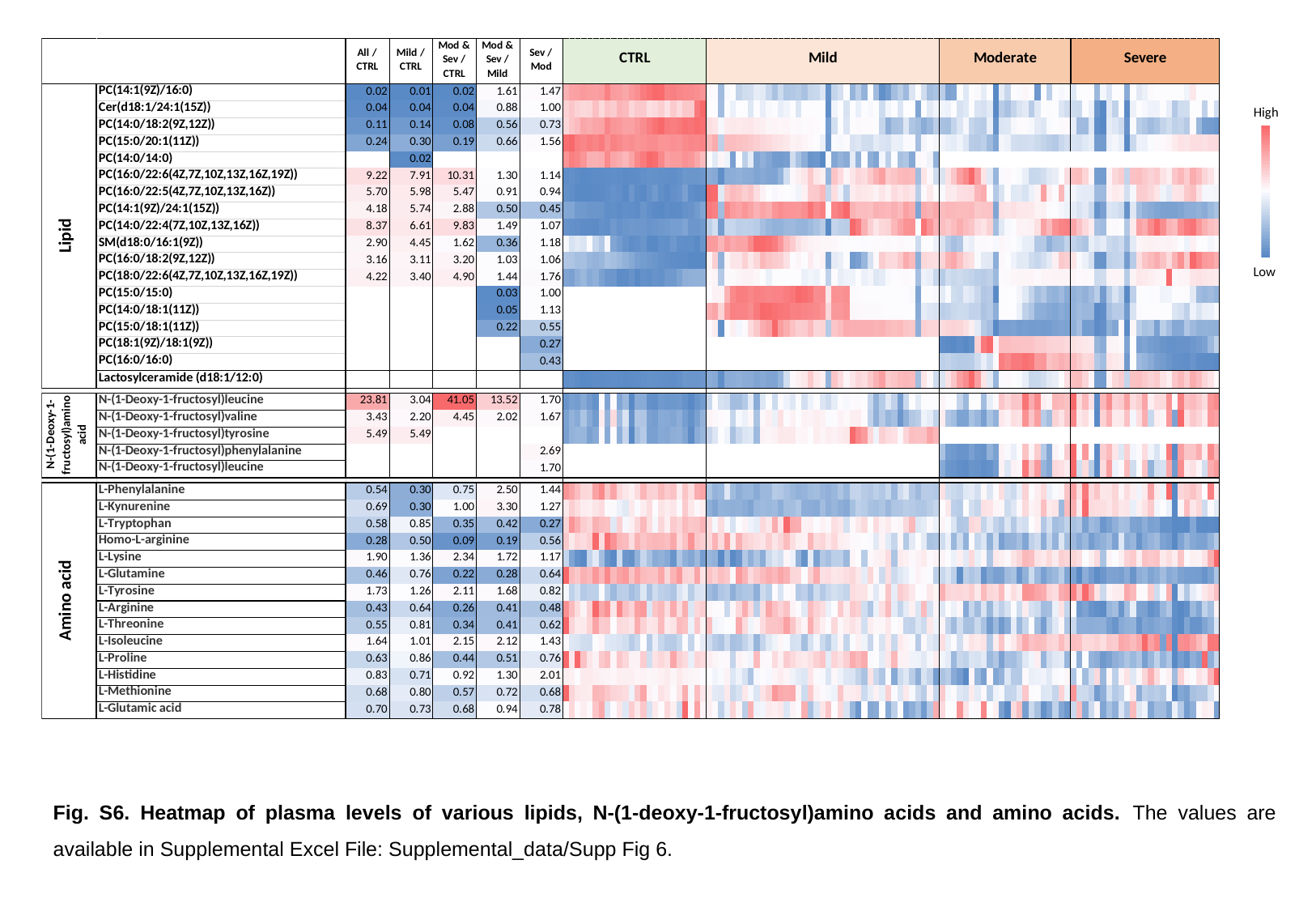

High
Low
Fig. S6. Heatmap of plasma levels of various lipids, N-(1-deoxy-1-fructosyl)amino acids and amino acids. The values are available in Supplemental Excel File: Supplemental_data/Supp Fig 6.

## Slide 8
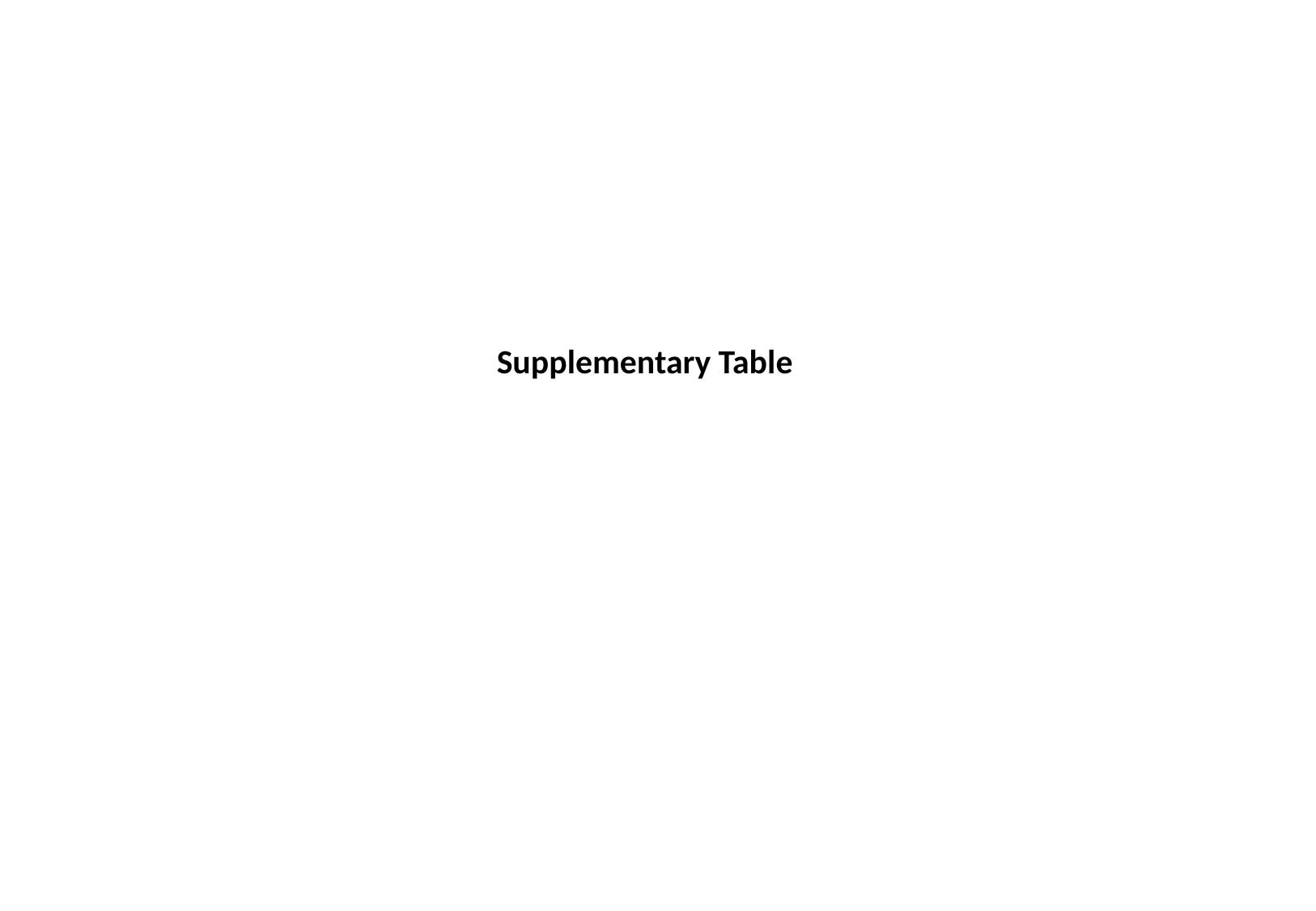

Supplementary Table

## Slide 9
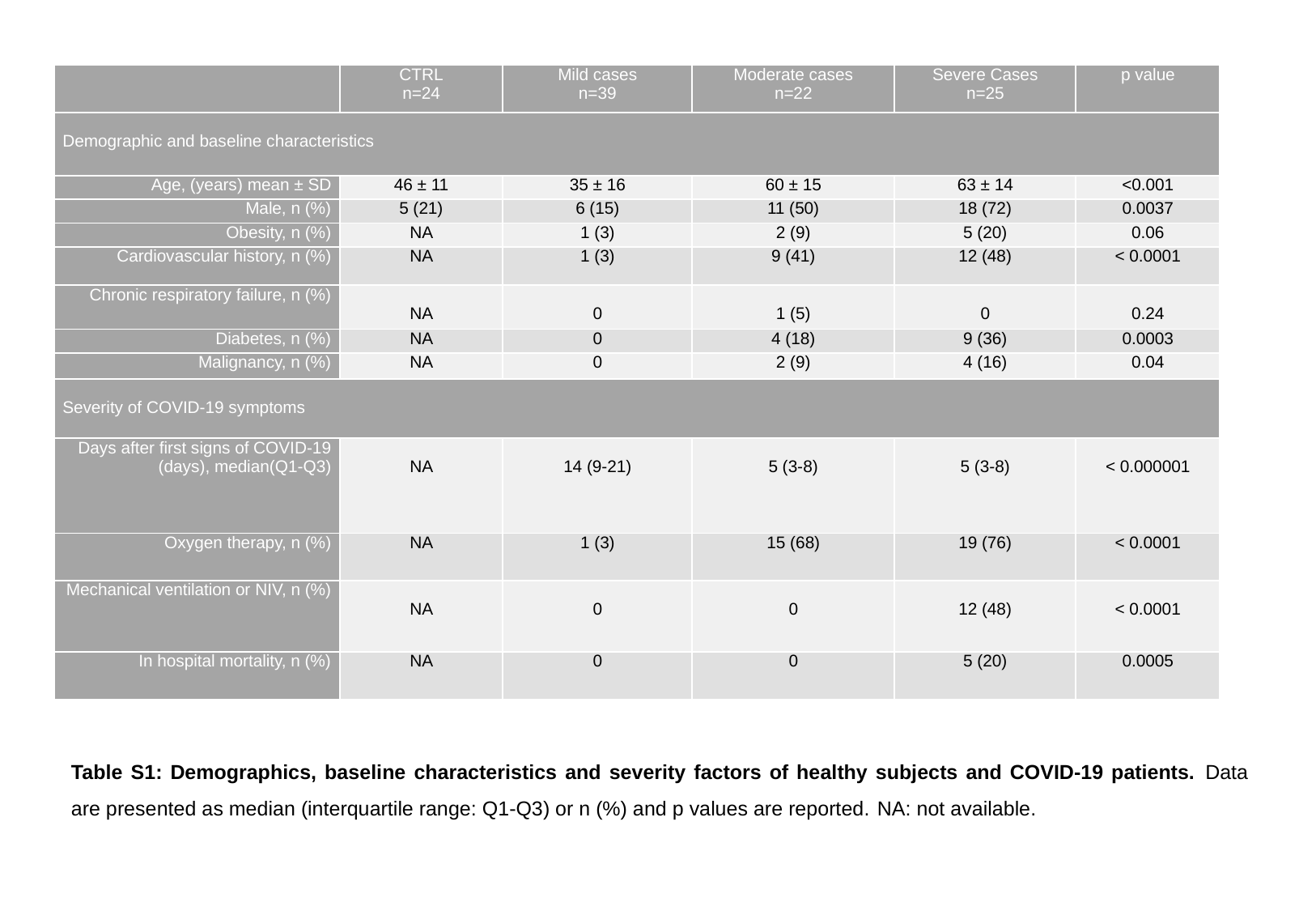

| | CTRL n=24 | Mild cases n=39 | Moderate cases n=22 | Severe Cases n=25 | p value |
| --- | --- | --- | --- | --- | --- |
| Demographic and baseline characteristics | | | | | |
| Age, (years) mean ± SD | 46 ± 11 | 35 ± 16 | 60 ± 15 | 63 ± 14 | <0.001 |
| Male, n (%) | 5 (21) | 6 (15) | 11 (50) | 18 (72) | 0.0037 |
| Obesity, n (%) | NA | 1 (3) | 2 (9) | 5 (20) | 0.06 |
| Cardiovascular history, n (%) | NA | 1 (3) | 9 (41) | 12 (48) | < 0.0001 |
| Chronic respiratory failure, n (%) | NA | 0 | 1 (5) | 0 | 0.24 |
| Diabetes, n (%) | NA | 0 | 4 (18) | 9 (36) | 0.0003 |
| Malignancy, n (%) | NA | 0 | 2 (9) | 4 (16) | 0.04 |
| Severity of COVID-19 symptoms | | | | | |
| Days after first signs of COVID-19 (days), median(Q1-Q3) | NA | 14 (9-21) | 5 (3-8) | 5 (3-8) | < 0.000001 |
| Oxygen therapy, n (%) | NA | 1 (3) | 15 (68) | 19 (76) | < 0.0001 |
| Mechanical ventilation or NIV, n (%) | NA | 0 | 0 | 12 (48) | < 0.0001 |
| In hospital mortality, n (%) | NA | 0 | 0 | 5 (20) | 0.0005 |
Table S1: Demographics, baseline characteristics and severity factors of healthy subjects and COVID-19 patients. Data are presented as median (interquartile range: Q1-Q3) or n (%) and p values are reported. NA: not available.

## Slide 10
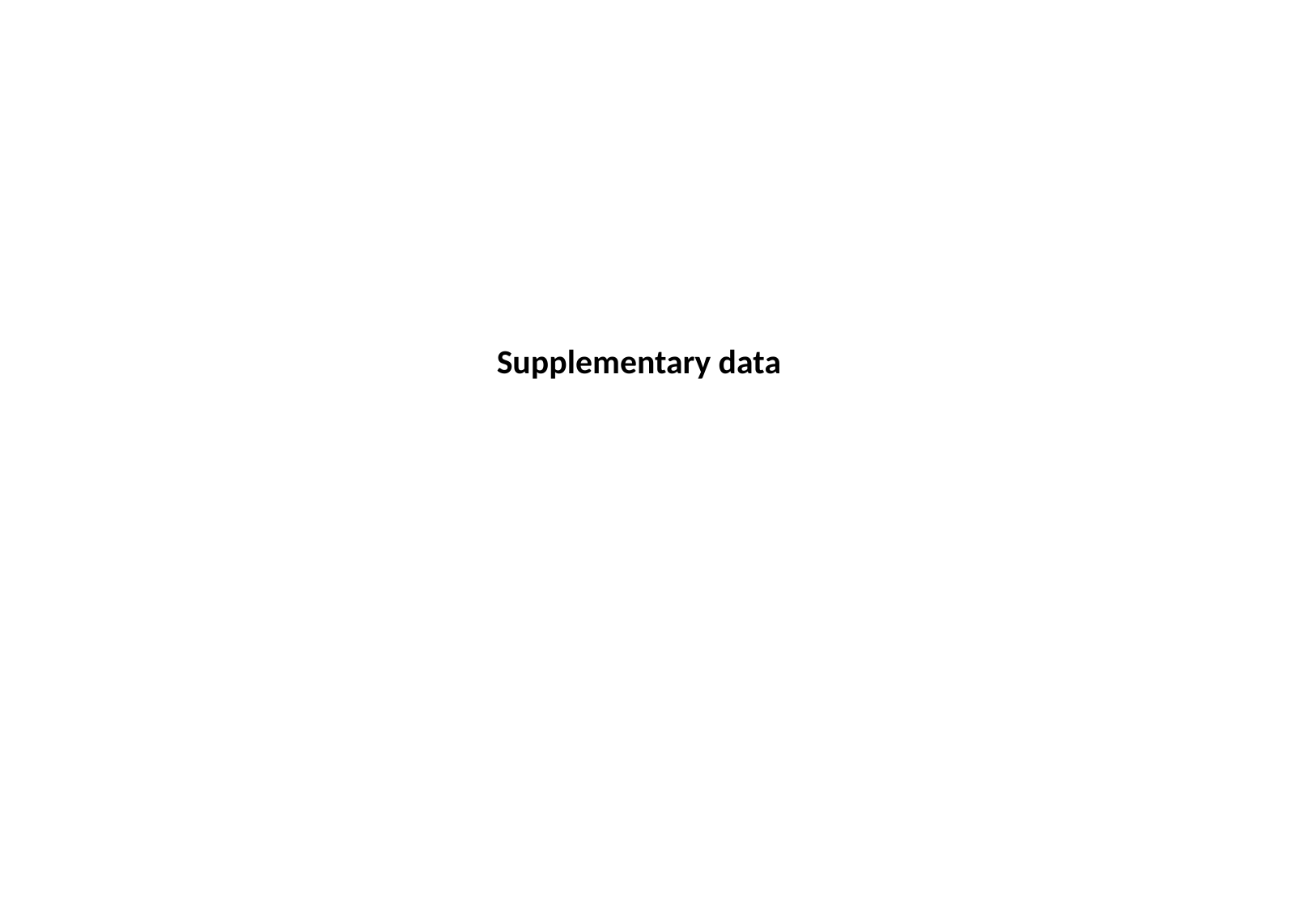

Supplementary data

## Slide 11
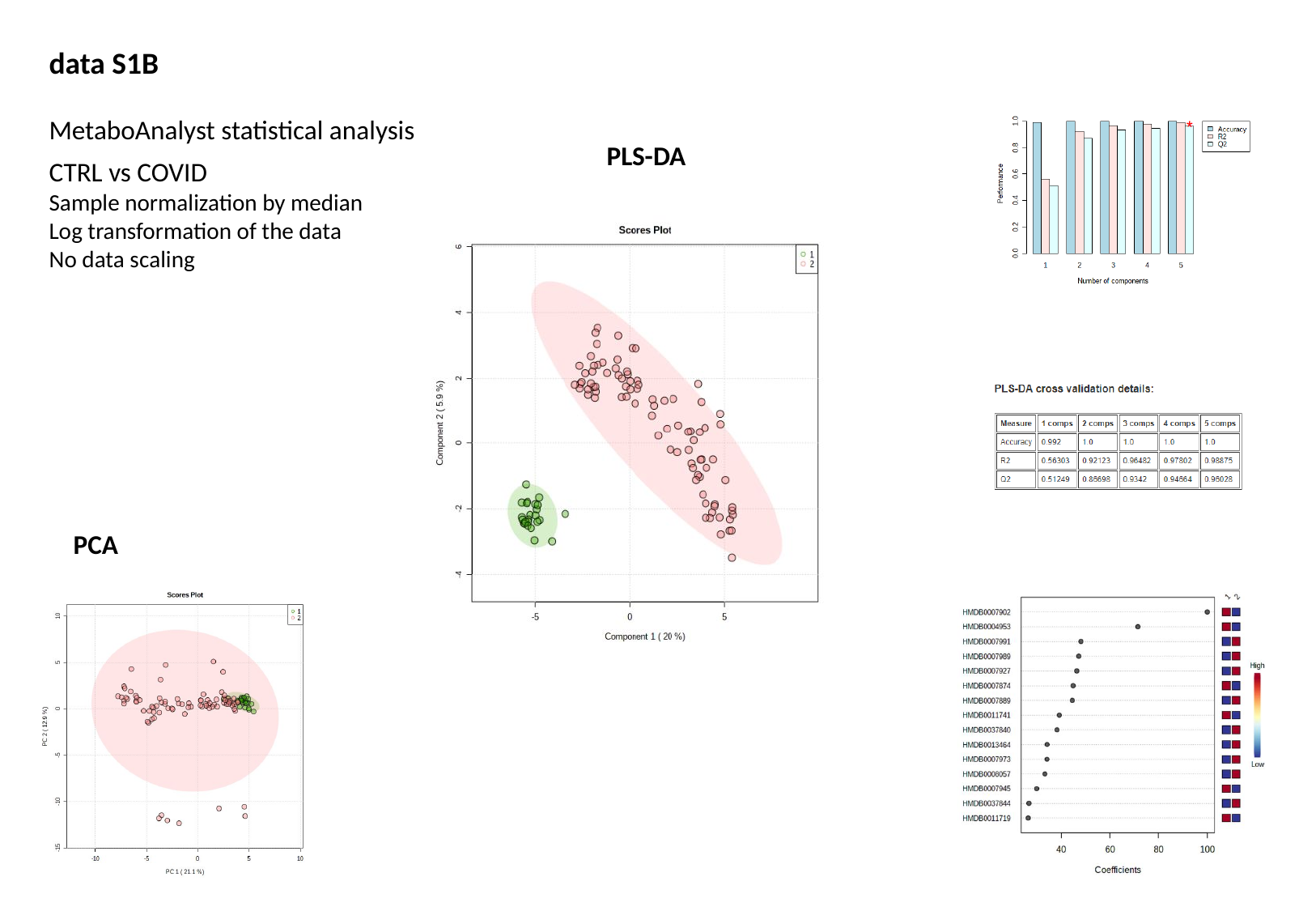

data S1B
MetaboAnalyst statistical analysis
CTRL vs COVID
Sample normalization by median
Log transformation of the data
No data scaling
PLS-DA
PCA

## Slide 12
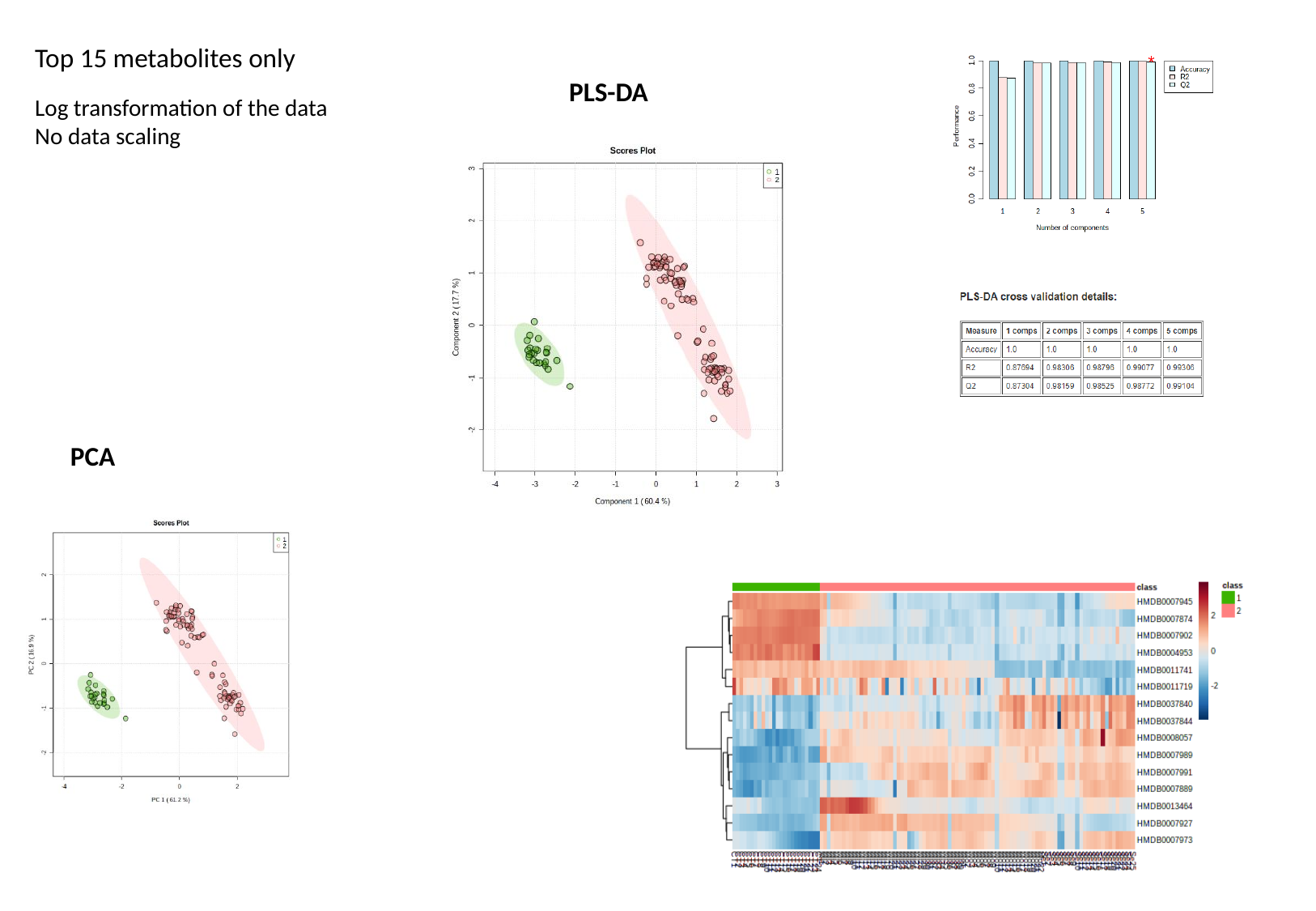

Top 15 metabolites only
Log transformation of the data
No data scaling
PLS-DA
PCA

## Slide 13
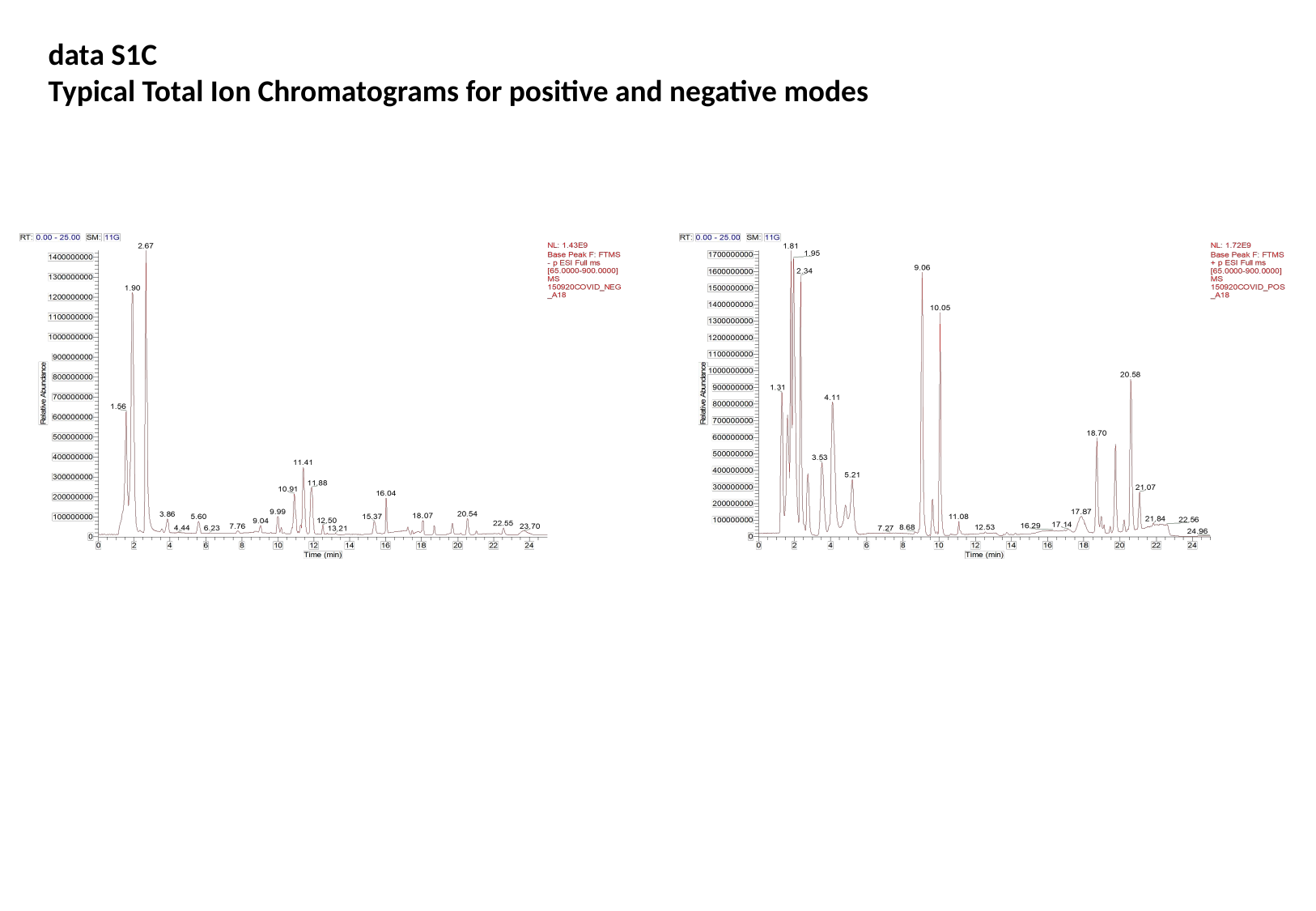

data S1C
Typical Total Ion Chromatograms for positive and negative modes

## Slide 14
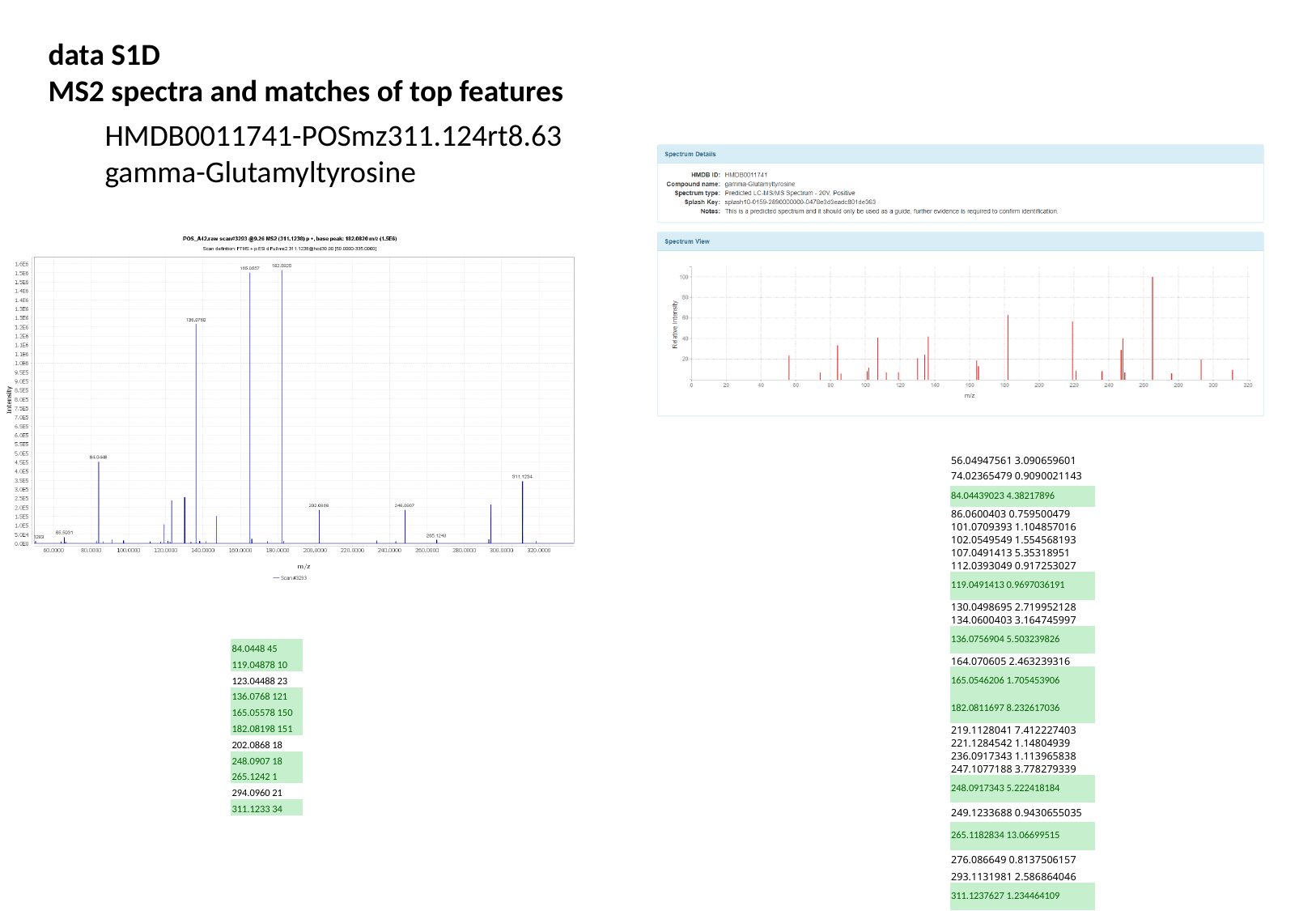

data S1D
MS2 spectra and matches of top features
HMDB0011741-POSmz311.124rt8.63
gamma-Glutamyltyrosine
| 56.04947561 3.090659601 |
| --- |
| 74.02365479 0.9090021143 |
| 84.04439023 4.38217896 |
| 86.0600403 0.759500479 |
| 101.0709393 1.104857016 |
| 102.0549549 1.554568193 |
| 107.0491413 5.35318951 |
| 112.0393049 0.917253027 |
| 119.0491413 0.9697036191 |
| 130.0498695 2.719952128 |
| 134.0600403 3.164745997 |
| 136.0756904 5.503239826 |
| 164.070605 2.463239316 |
| 165.0546206 1.705453906 |
| 182.0811697 8.232617036 |
| 219.1128041 7.412227403 |
| 221.1284542 1.14804939 |
| 236.0917343 1.113965838 |
| 247.1077188 3.778279339 |
| 248.0917343 5.222418184 |
| 249.1233688 0.9430655035 |
| 265.1182834 13.06699515 |
| 276.086649 0.8137506157 |
| 293.1131981 2.586864046 |
| 311.1237627 1.234464109 |
| 84.0448 45 |
| --- |
| 119.04878 10 |
| 123.04488 23 |
| 136.0768 121 |
| 165.05578 150 |
| 182.08198 151 |
| 202.0868 18 |
| 248.0907 18 |
| 265.1242 1 |
| 294.0960 21 |
| 311.1233 34 |

## Slide 15
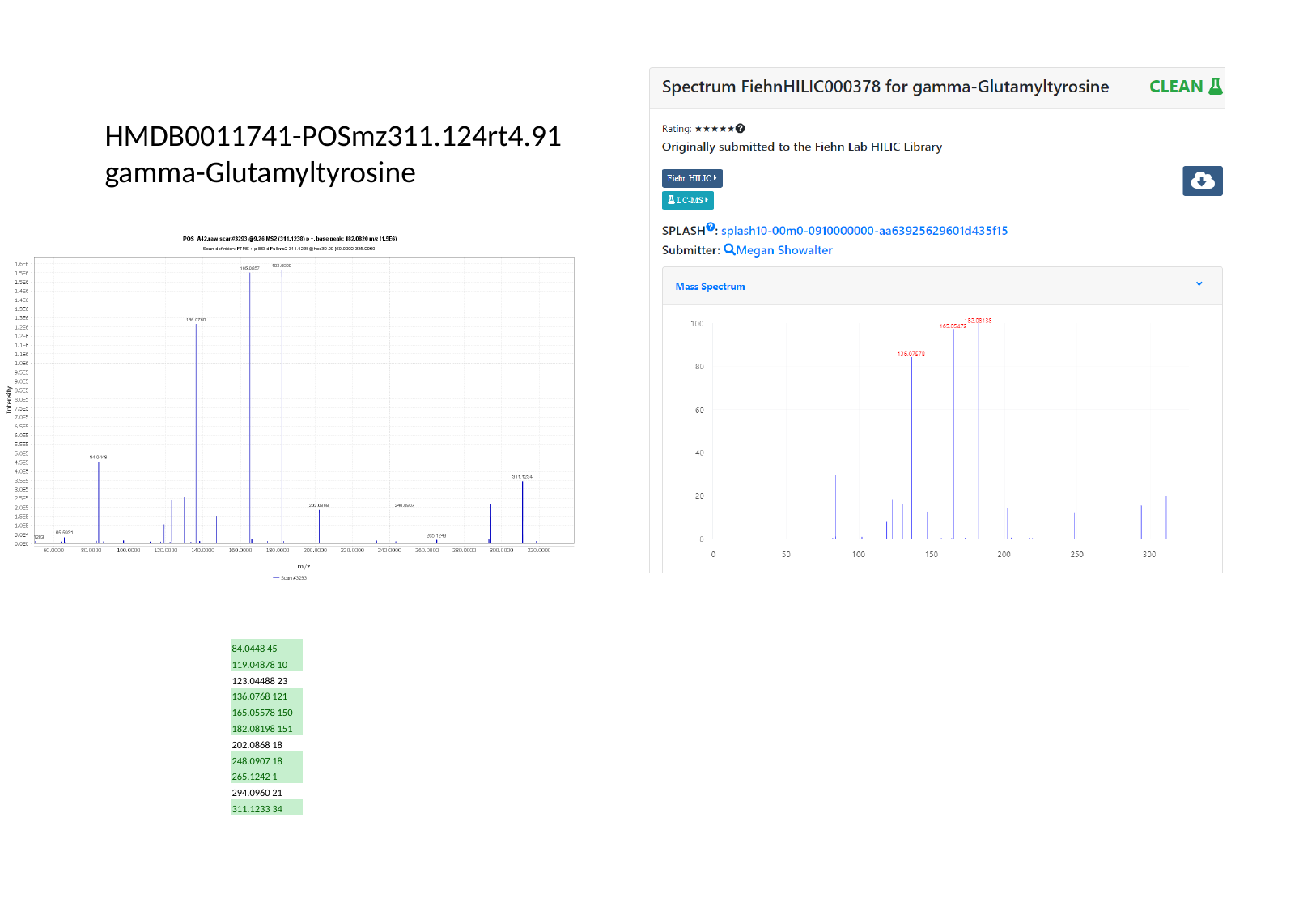

HMDB0011741-POSmz311.124rt4.91
gamma-Glutamyltyrosine
| 84.0448 45 |
| --- |
| 119.04878 10 |
| 123.04488 23 |
| 136.0768 121 |
| 165.05578 150 |
| 182.08198 151 |
| 202.0868 18 |
| 248.0907 18 |
| 265.1242 1 |
| 294.0960 21 |
| 311.1233 34 |

## Slide 16
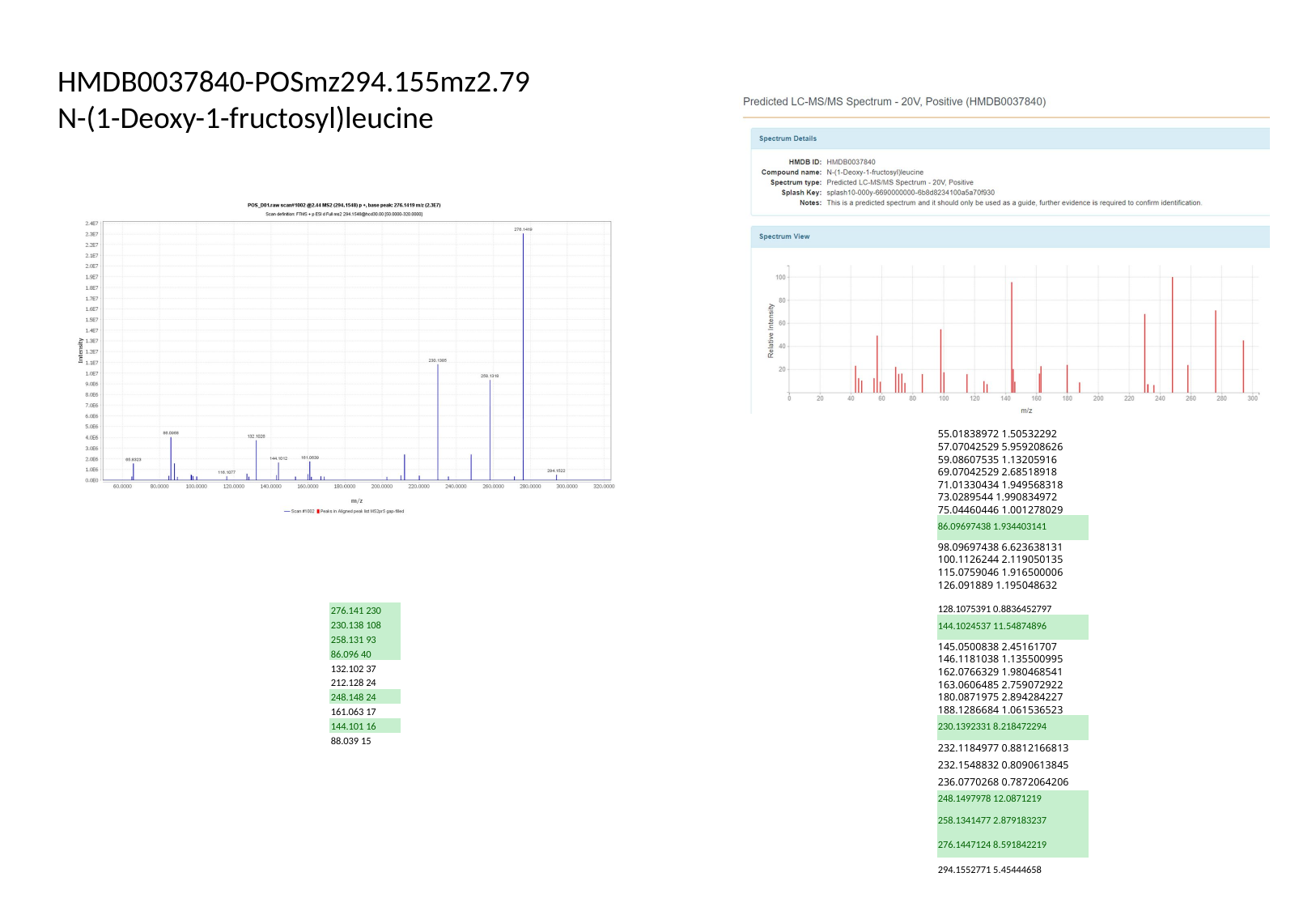

HMDB0037840-POSmz294.155mz2.79
N-(1-Deoxy-1-fructosyl)leucine
| 55.01838972 1.50532292 |
| --- |
| 57.07042529 5.959208626 |
| 59.08607535 1.13205916 |
| 69.07042529 2.68518918 |
| 71.01330434 1.949568318 |
| 73.0289544 1.990834972 |
| 75.04460446 1.001278029 |
| 86.09697438 1.934403141 |
| 98.09697438 6.623638131 |
| 100.1126244 2.119050135 |
| 115.0759046 1.916500006 |
| 126.091889 1.195048632 |
| 128.1075391 0.8836452797 |
| 144.1024537 11.54874896 |
| 145.0500838 2.45161707 |
| 146.1181038 1.135500995 |
| 162.0766329 1.980468541 |
| 163.0606485 2.759072922 |
| 180.0871975 2.894284227 |
| 188.1286684 1.061536523 |
| 230.1392331 8.218472294 |
| 232.1184977 0.8812166813 |
| 232.1548832 0.8090613845 |
| 236.0770268 0.7872064206 |
| 248.1497978 12.0871219 |
| 258.1341477 2.879183237 |
| 276.1447124 8.591842219 |
| 294.1552771 5.45444658 |
| 276.141 230 |
| --- |
| 230.138 108 |
| 258.131 93 |
| 86.096 40 |
| 132.102 37 |
| 212.128 24 |
| 248.148 24 |
| 161.063 17 |
| 144.101 16 |
| 88.039 15 |

## Slide 17
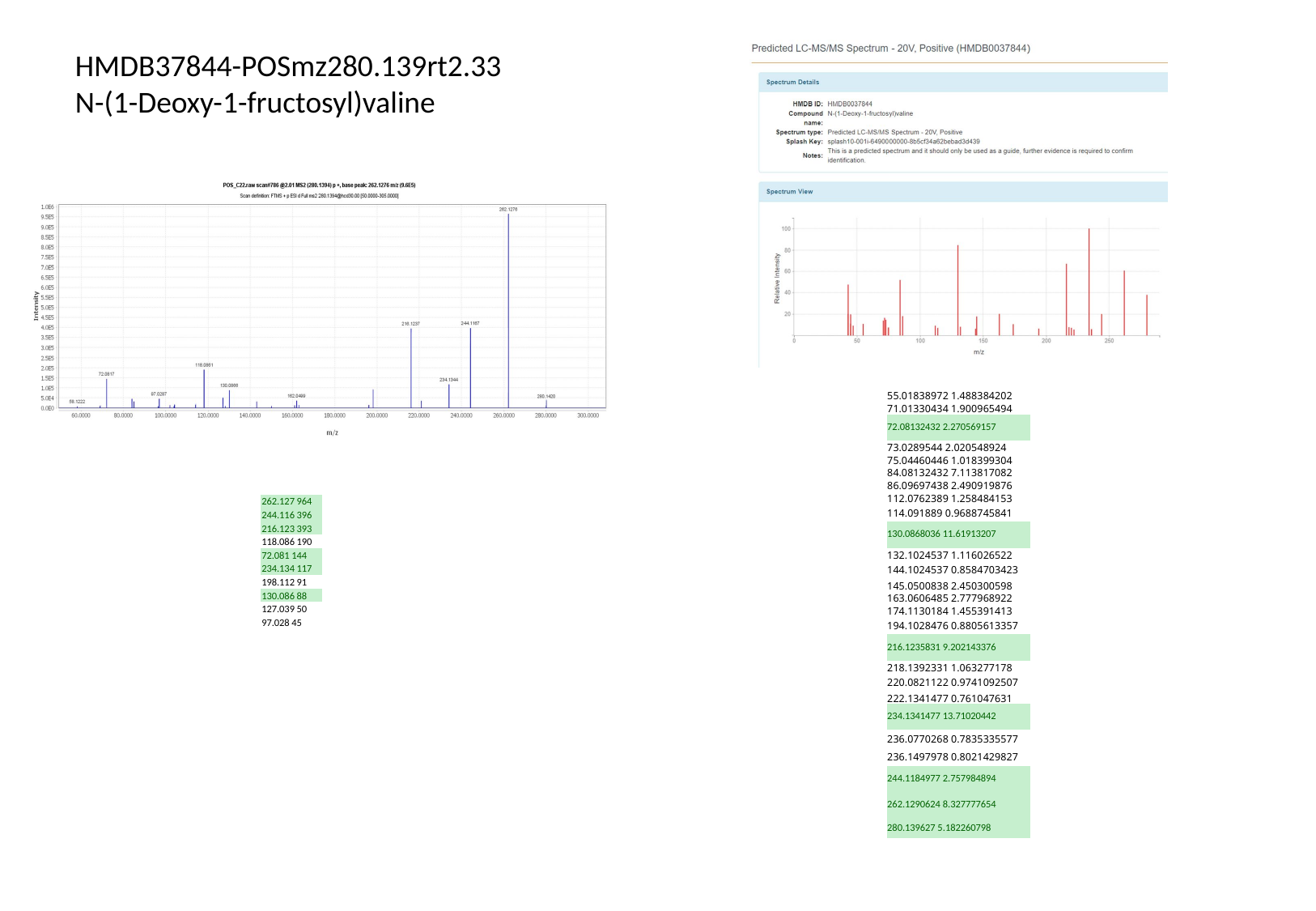

HMDB37844-POSmz280.139rt2.33
N-(1-Deoxy-1-fructosyl)valine
| 55.01838972 1.488384202 |
| --- |
| 71.01330434 1.900965494 |
| 72.08132432 2.270569157 |
| 73.0289544 2.020548924 |
| 75.04460446 1.018399304 |
| 84.08132432 7.113817082 |
| 86.09697438 2.490919876 |
| 112.0762389 1.258484153 |
| 114.091889 0.9688745841 |
| 130.0868036 11.61913207 |
| 132.1024537 1.116026522 |
| 144.1024537 0.8584703423 |
| 145.0500838 2.450300598 |
| 163.0606485 2.777968922 |
| 174.1130184 1.455391413 |
| 194.1028476 0.8805613357 |
| 216.1235831 9.202143376 |
| 218.1392331 1.063277178 |
| 220.0821122 0.9741092507 |
| 222.1341477 0.761047631 |
| 234.1341477 13.71020442 |
| 236.0770268 0.7835335577 |
| 236.1497978 0.8021429827 |
| 244.1184977 2.757984894 |
| 262.1290624 8.327777654 |
| 280.139627 5.182260798 |
| 262.127 964 |
| --- |
| 244.116 396 |
| 216.123 393 |
| 118.086 190 |
| 72.081 144 |
| 234.134 117 |
| 198.112 91 |
| 130.086 88 |
| 127.039 50 |
| 97.028 45 |

## Slide 18
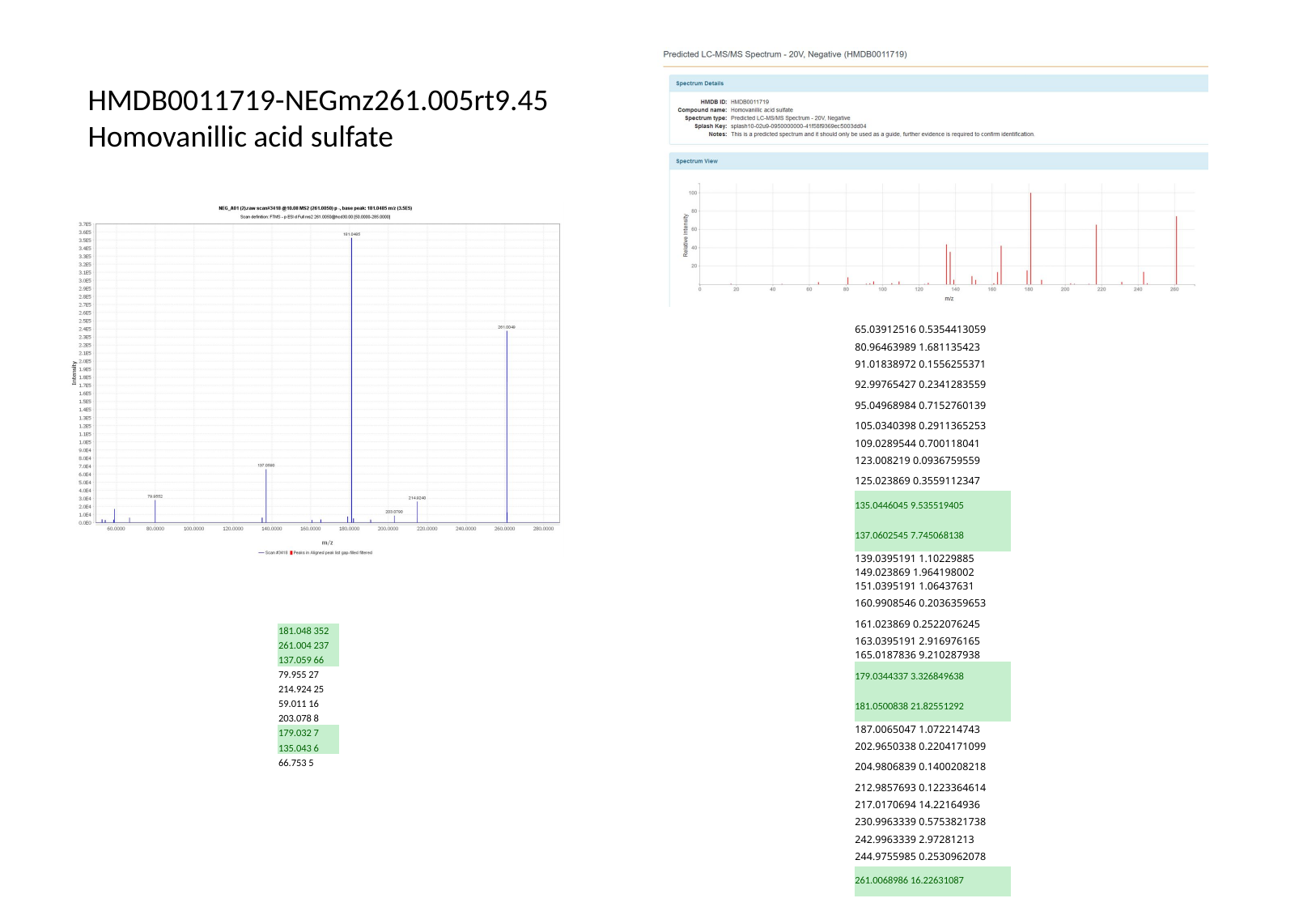

HMDB0011719-NEGmz261.005rt9.45
Homovanillic acid sulfate
| 65.03912516 0.5354413059 |
| --- |
| 80.96463989 1.681135423 |
| 91.01838972 0.1556255371 |
| 92.99765427 0.2341283559 |
| 95.04968984 0.7152760139 |
| 105.0340398 0.2911365253 |
| 109.0289544 0.700118041 |
| 123.008219 0.0936759559 |
| 125.023869 0.3559112347 |
| 135.0446045 9.535519405 |
| 137.0602545 7.745068138 |
| 139.0395191 1.10229885 |
| 149.023869 1.964198002 |
| 151.0395191 1.06437631 |
| 160.9908546 0.2036359653 |
| 161.023869 0.2522076245 |
| 163.0395191 2.916976165 |
| 165.0187836 9.210287938 |
| 179.0344337 3.326849638 |
| 181.0500838 21.82551292 |
| 187.0065047 1.072214743 |
| 202.9650338 0.2204171099 |
| 204.9806839 0.1400208218 |
| 212.9857693 0.1223364614 |
| 217.0170694 14.22164936 |
| 230.9963339 0.5753821738 |
| 242.9963339 2.97281213 |
| 244.9755985 0.2530962078 |
| 261.0068986 16.22631087 |
| 181.048 352 |
| --- |
| 261.004 237 |
| 137.059 66 |
| 79.955 27 |
| 214.924 25 |
| 59.011 16 |
| 203.078 8 |
| 179.032 7 |
| 135.043 6 |
| 66.753 5 |

## Slide 19
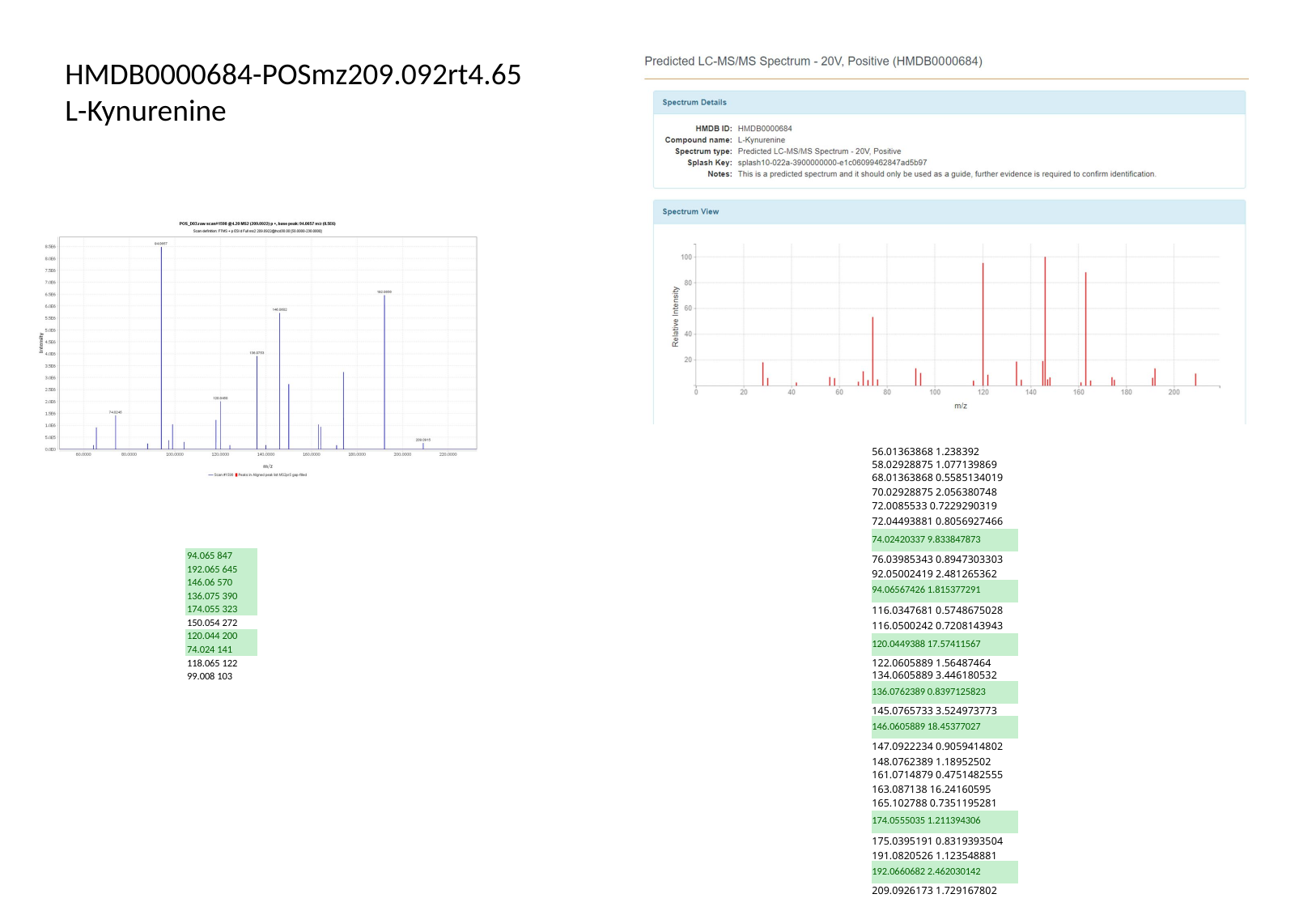

HMDB0000684-POSmz209.092rt4.65
L-Kynurenine
| 56.01363868 1.238392 |
| --- |
| 58.02928875 1.077139869 |
| 68.01363868 0.5585134019 |
| 70.02928875 2.056380748 |
| 72.0085533 0.7229290319 |
| 72.04493881 0.8056927466 |
| 74.02420337 9.833847873 |
| 76.03985343 0.8947303303 |
| 92.05002419 2.481265362 |
| 94.06567426 1.815377291 |
| 116.0347681 0.5748675028 |
| 116.0500242 0.7208143943 |
| 120.0449388 17.57411567 |
| 122.0605889 1.56487464 |
| 134.0605889 3.446180532 |
| 136.0762389 0.8397125823 |
| 145.0765733 3.524973773 |
| 146.0605889 18.45377027 |
| 147.0922234 0.9059414802 |
| 148.0762389 1.18952502 |
| 161.0714879 0.4751482555 |
| 163.087138 16.24160595 |
| 165.102788 0.7351195281 |
| 174.0555035 1.211394306 |
| 175.0395191 0.8319393504 |
| 191.0820526 1.123548881 |
| 192.0660682 2.462030142 |
| 209.0926173 1.729167802 |
| 94.065 847 |
| --- |
| 192.065 645 |
| 146.06 570 |
| 136.075 390 |
| 174.055 323 |
| 150.054 272 |
| 120.044 200 |
| 74.024 141 |
| 118.065 122 |
| 99.008 103 |

## Slide 20
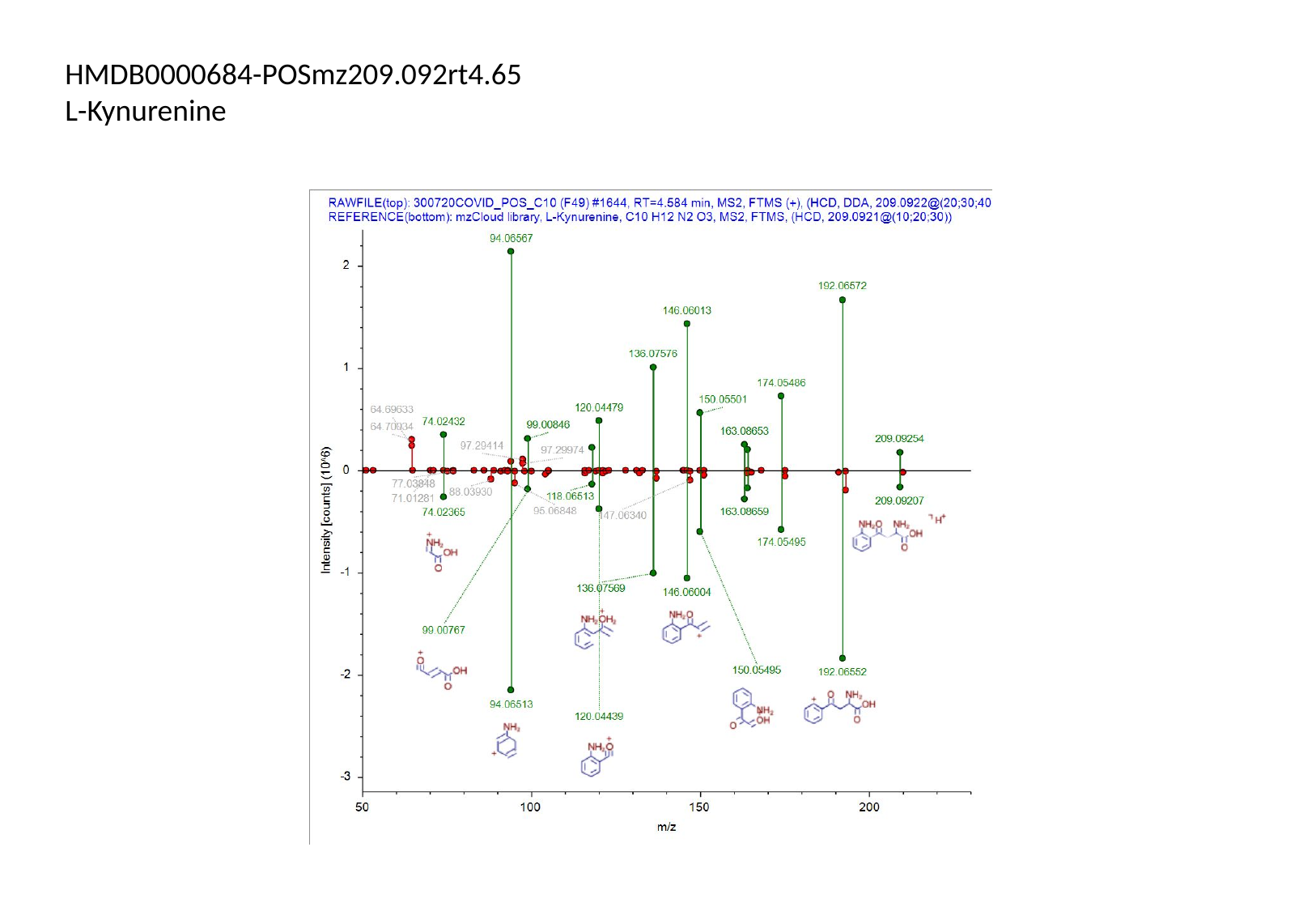

HMDB0000684-POSmz209.092rt4.65
L-Kynurenine

## Slide 21
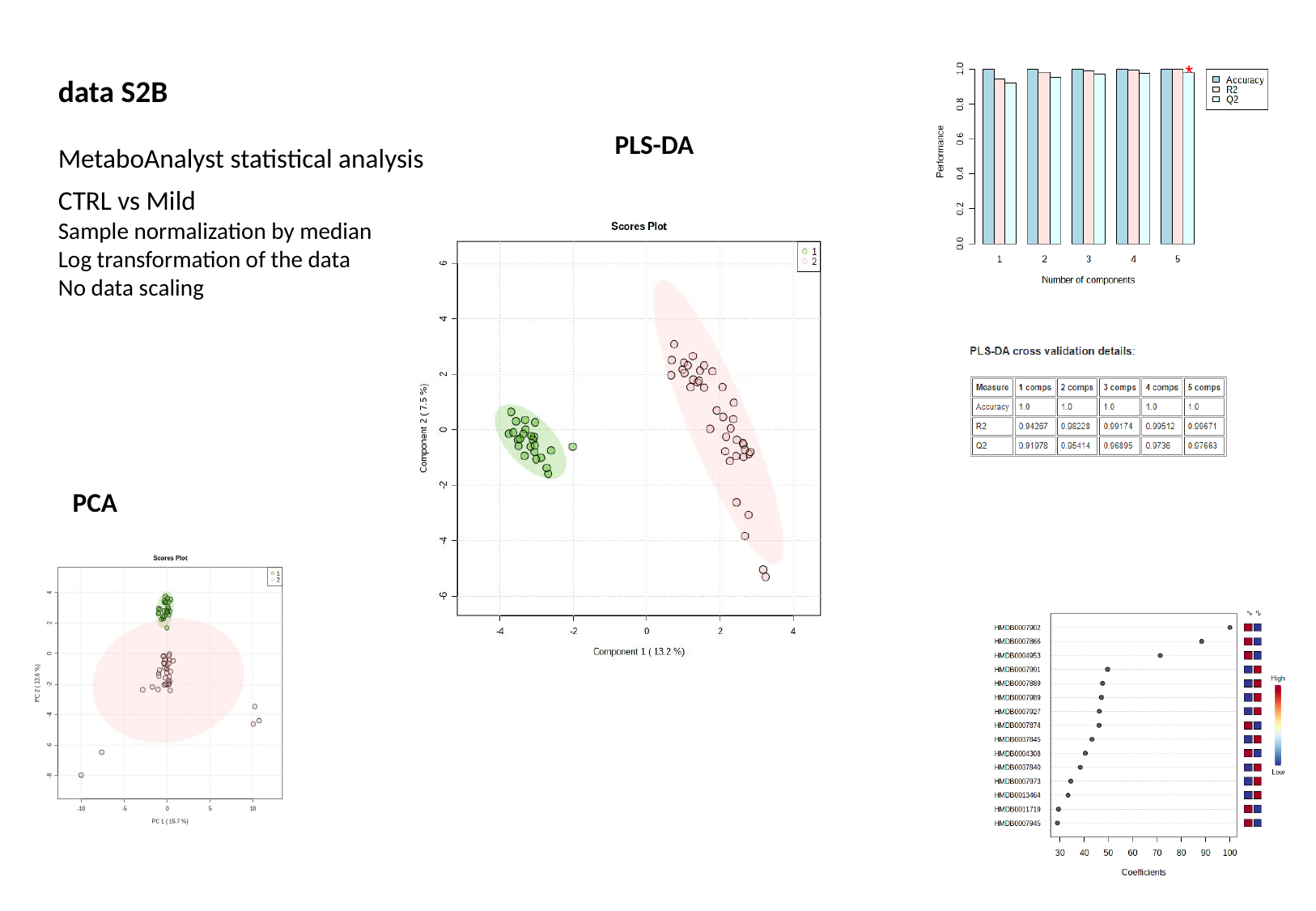

data S2B
MetaboAnalyst statistical analysis
CTRL vs Mild
Sample normalization by median
Log transformation of the data
No data scaling
PLS-DA
PCA

## Slide 22
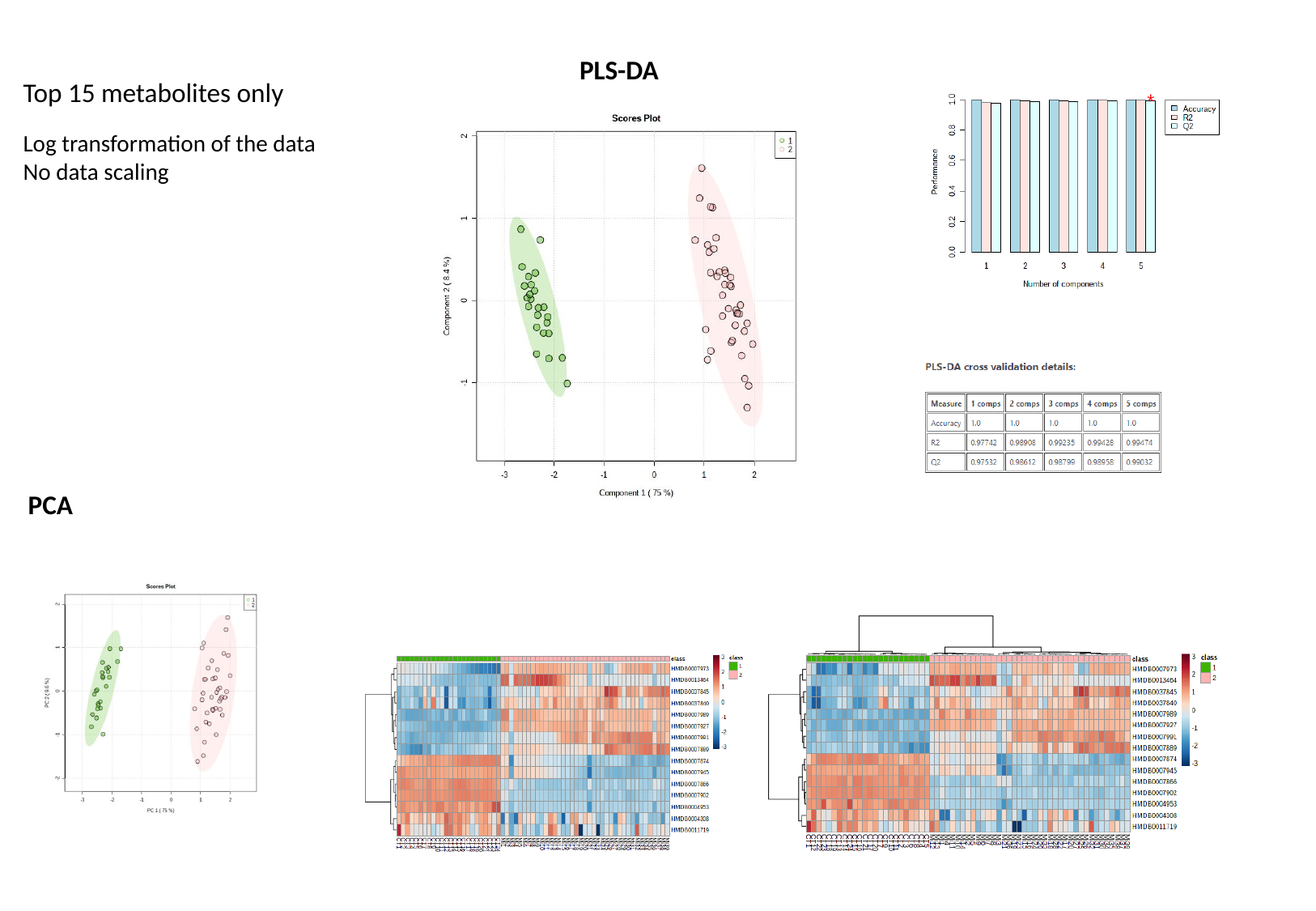

PLS-DA
Top 15 metabolites only
Log transformation of the data
No data scaling
PCA

## Slide 23
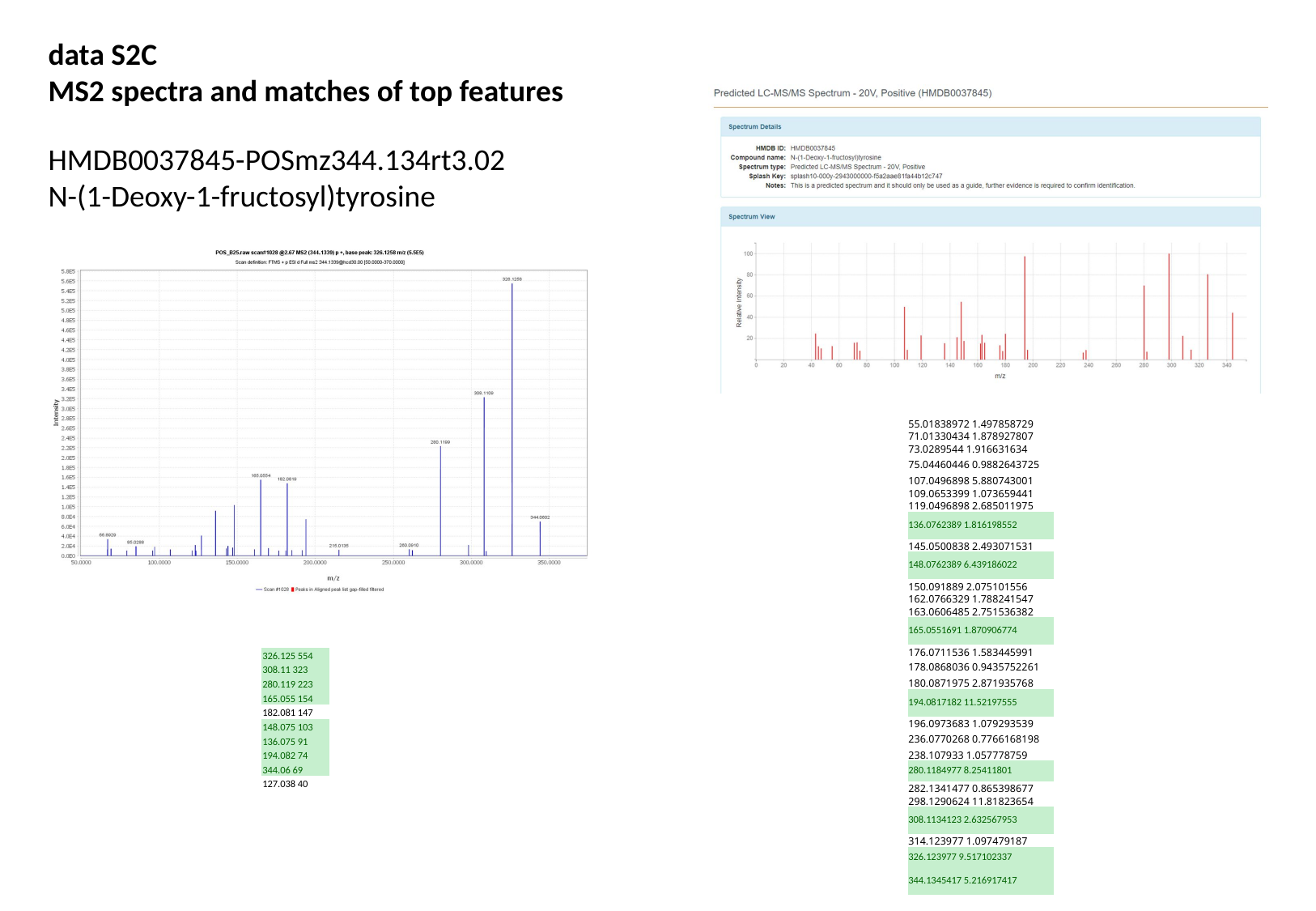

data S2C
MS2 spectra and matches of top features
HMDB0037845-POSmz344.134rt3.02
N-(1-Deoxy-1-fructosyl)tyrosine
| 55.01838972 1.497858729 |
| --- |
| 71.01330434 1.878927807 |
| 73.0289544 1.916631634 |
| 75.04460446 0.9882643725 |
| 107.0496898 5.880743001 |
| 109.0653399 1.073659441 |
| 119.0496898 2.685011975 |
| 136.0762389 1.816198552 |
| 145.0500838 2.493071531 |
| 148.0762389 6.439186022 |
| 150.091889 2.075101556 |
| 162.0766329 1.788241547 |
| 163.0606485 2.751536382 |
| 165.0551691 1.870906774 |
| 176.0711536 1.583445991 |
| 178.0868036 0.9435752261 |
| 180.0871975 2.871935768 |
| 194.0817182 11.52197555 |
| 196.0973683 1.079293539 |
| 236.0770268 0.7766168198 |
| 238.107933 1.057778759 |
| 280.1184977 8.25411801 |
| 282.1341477 0.865398677 |
| 298.1290624 11.81823654 |
| 308.1134123 2.632567953 |
| 314.123977 1.097479187 |
| 326.123977 9.517102337 |
| 344.1345417 5.216917417 |
| 326.125 554 |
| --- |
| 308.11 323 |
| 280.119 223 |
| 165.055 154 |
| 182.081 147 |
| 148.075 103 |
| 136.075 91 |
| 194.082 74 |
| 344.06 69 |
| 127.038 40 |

## Slide 24
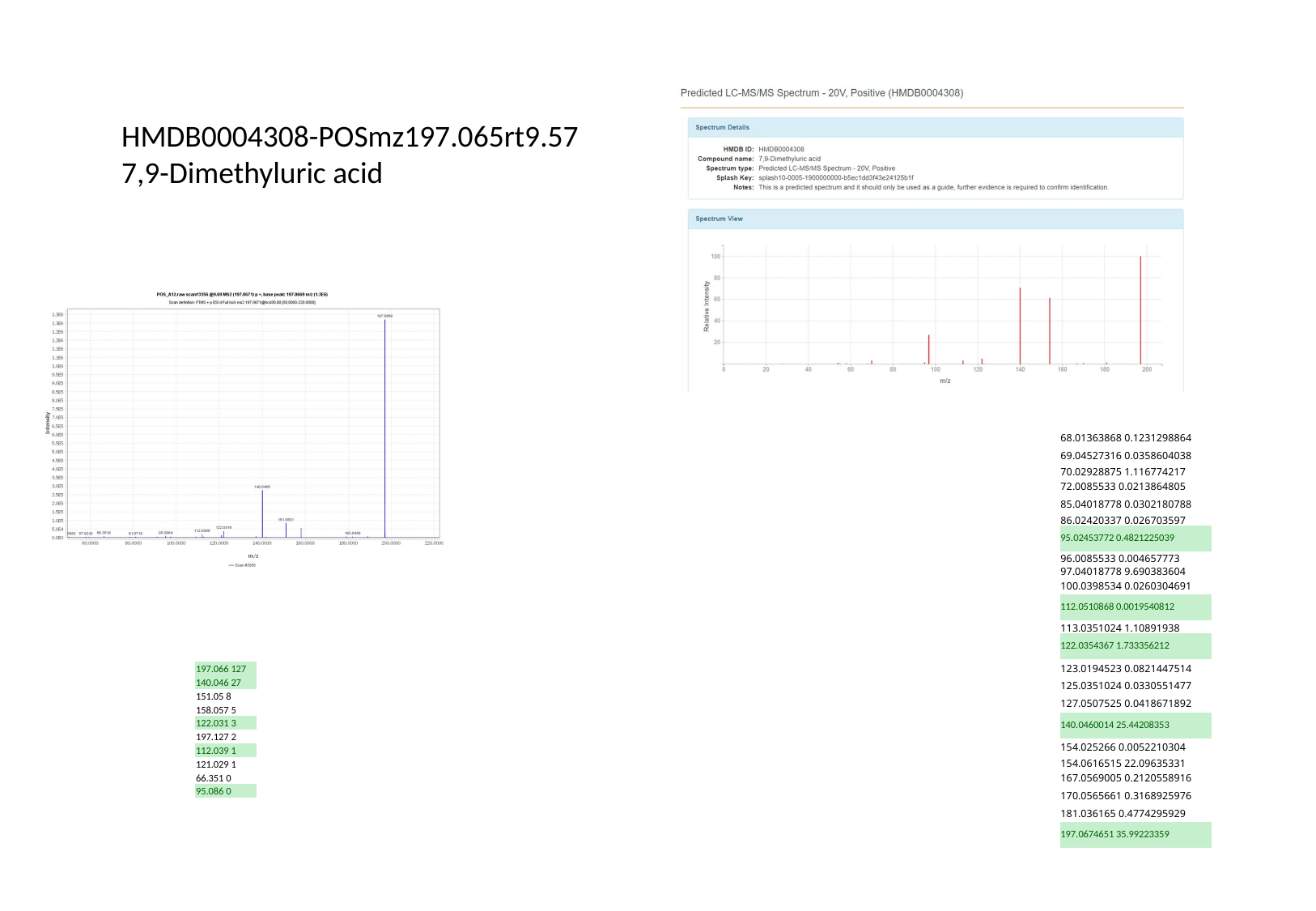

HMDB0004308-POSmz197.065rt9.57
7,9-Dimethyluric acid
| 68.01363868 0.1231298864 |
| --- |
| 69.04527316 0.0358604038 |
| 70.02928875 1.116774217 |
| 72.0085533 0.0213864805 |
| 85.04018778 0.0302180788 |
| 86.02420337 0.026703597 |
| 95.02453772 0.4821225039 |
| 96.0085533 0.004657773 |
| 97.04018778 9.690383604 |
| 100.0398534 0.0260304691 |
| 112.0510868 0.0019540812 |
| 113.0351024 1.10891938 |
| 122.0354367 1.733356212 |
| 123.0194523 0.0821447514 |
| 125.0351024 0.0330551477 |
| 127.0507525 0.0418671892 |
| 140.0460014 25.44208353 |
| 154.025266 0.0052210304 |
| 154.0616515 22.09635331 |
| 167.0569005 0.2120558916 |
| 170.0565661 0.3168925976 |
| 181.036165 0.4774295929 |
| 197.0674651 35.99223359 |
| 197.066 127 |
| --- |
| 140.046 27 |
| 151.05 8 |
| 158.057 5 |
| 122.031 3 |
| 197.127 2 |
| 112.039 1 |
| 121.029 1 |
| 66.351 0 |
| 95.086 0 |

## Slide 25
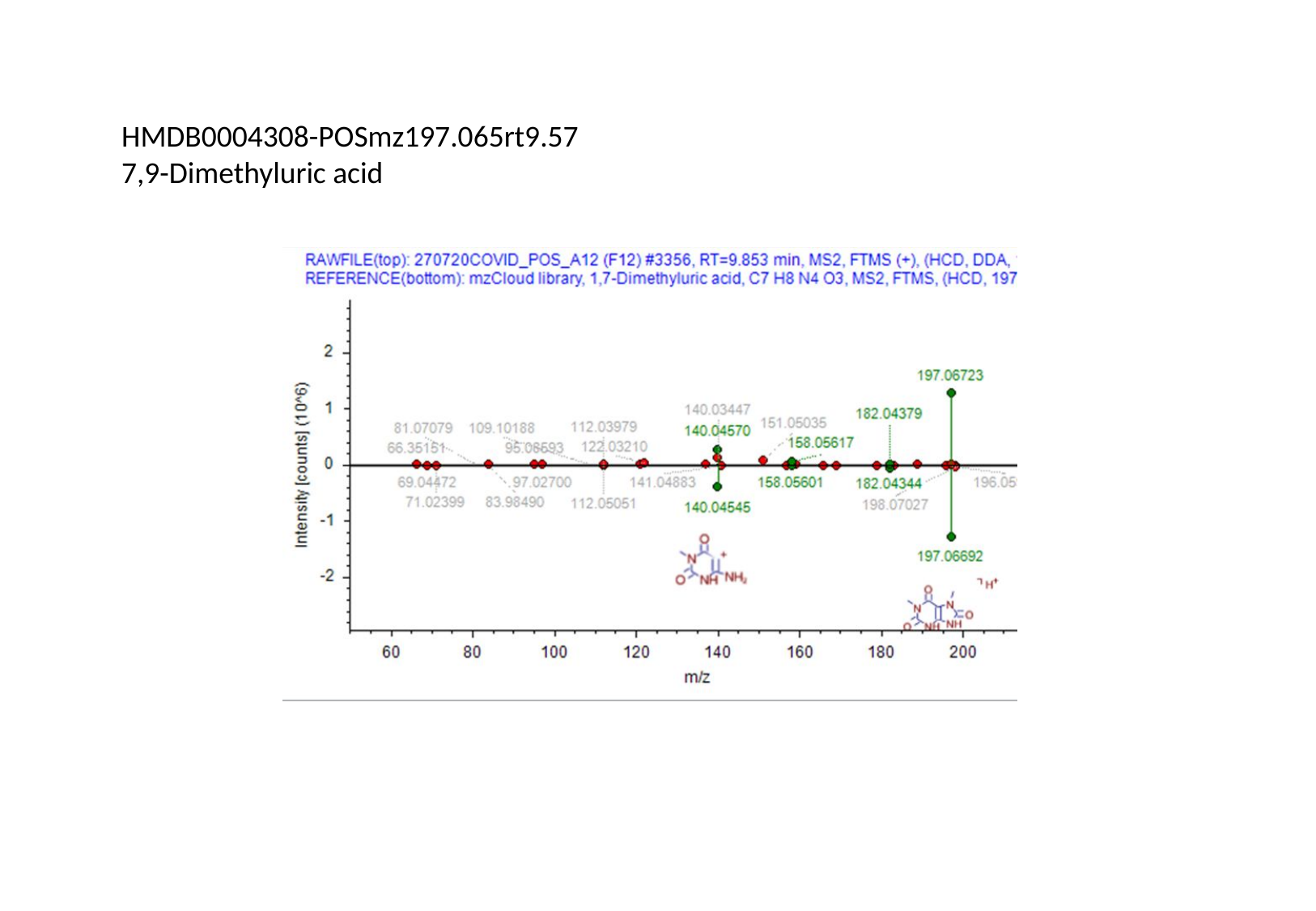

HMDB0004308-POSmz197.065rt9.57
7,9-Dimethyluric acid

## Slide 26
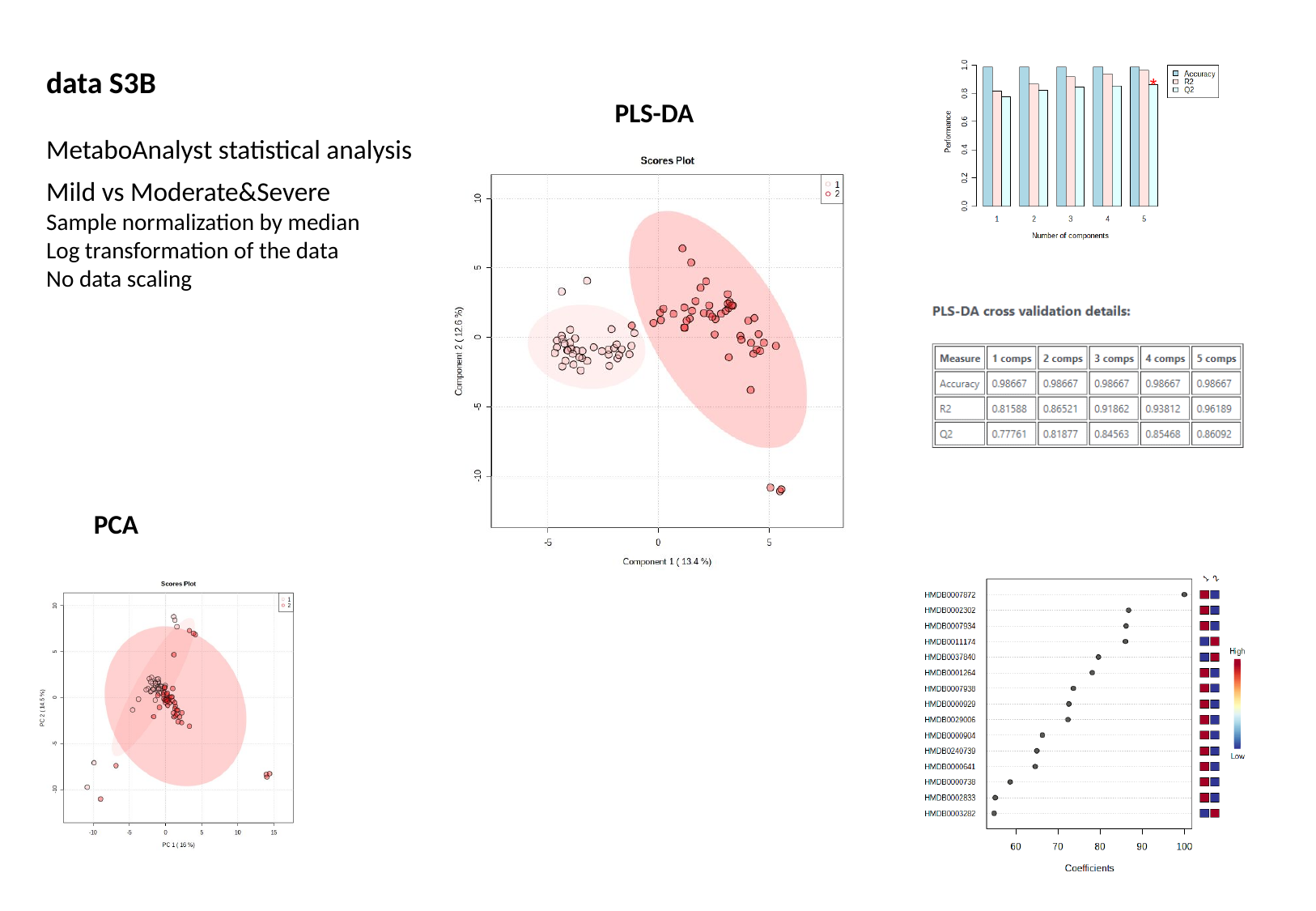

data S3B
MetaboAnalyst statistical analysis
Mild vs Moderate&Severe
Sample normalization by median
Log transformation of the data
No data scaling
PLS-DA
PCA

## Slide 27
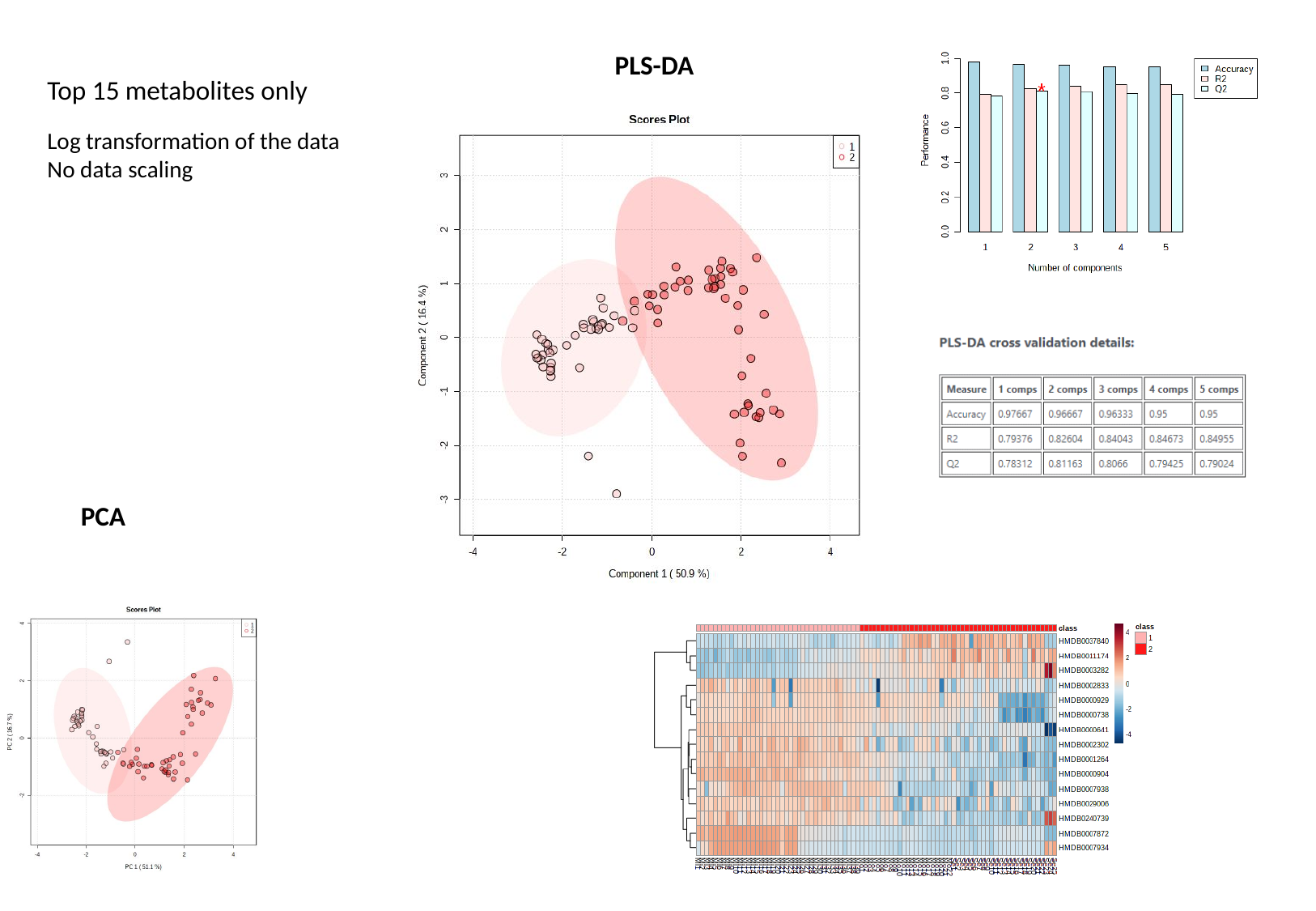

PLS-DA
Top 15 metabolites only
Log transformation of the data
No data scaling
PCA

## Slide 28
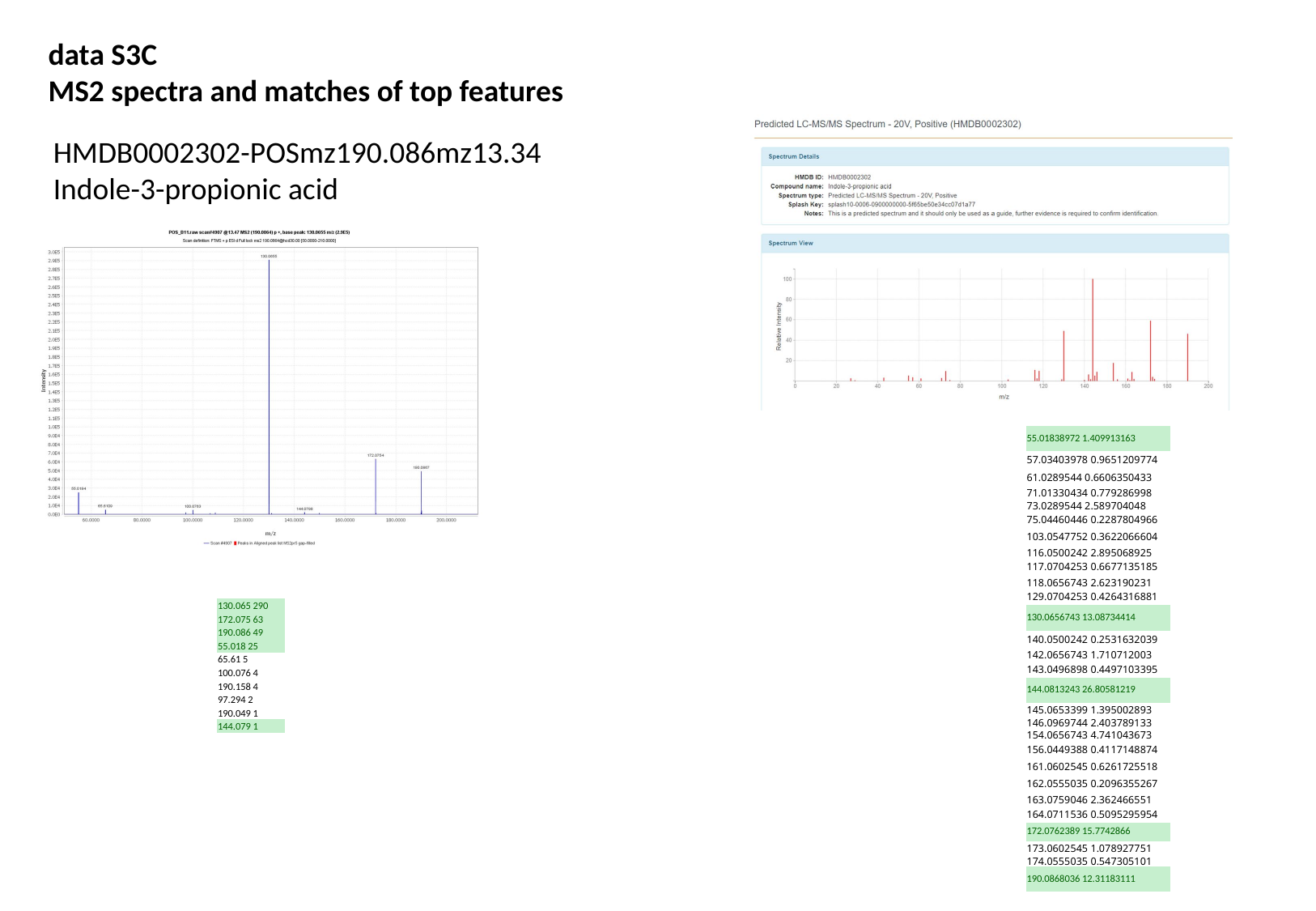

data S3C
MS2 spectra and matches of top features
HMDB0002302-POSmz190.086mz13.34
Indole-3-propionic acid
| 55.01838972 1.409913163 |
| --- |
| 57.03403978 0.9651209774 |
| 61.0289544 0.6606350433 |
| 71.01330434 0.779286998 |
| 73.0289544 2.589704048 |
| 75.04460446 0.2287804966 |
| 103.0547752 0.3622066604 |
| 116.0500242 2.895068925 |
| 117.0704253 0.6677135185 |
| 118.0656743 2.623190231 |
| 129.0704253 0.4264316881 |
| 130.0656743 13.08734414 |
| 140.0500242 0.2531632039 |
| 142.0656743 1.710712003 |
| 143.0496898 0.4497103395 |
| 144.0813243 26.80581219 |
| 145.0653399 1.395002893 |
| 146.0969744 2.403789133 |
| 154.0656743 4.741043673 |
| 156.0449388 0.4117148874 |
| 161.0602545 0.6261725518 |
| 162.0555035 0.2096355267 |
| 163.0759046 2.362466551 |
| 164.0711536 0.5095295954 |
| 172.0762389 15.7742866 |
| 173.0602545 1.078927751 |
| 174.0555035 0.547305101 |
| 190.0868036 12.31183111 |
| 130.065 290 |
| --- |
| 172.075 63 |
| 190.086 49 |
| 55.018 25 |
| 65.61 5 |
| 100.076 4 |
| 190.158 4 |
| 97.294 2 |
| 190.049 1 |
| 144.079 1 |

## Slide 29
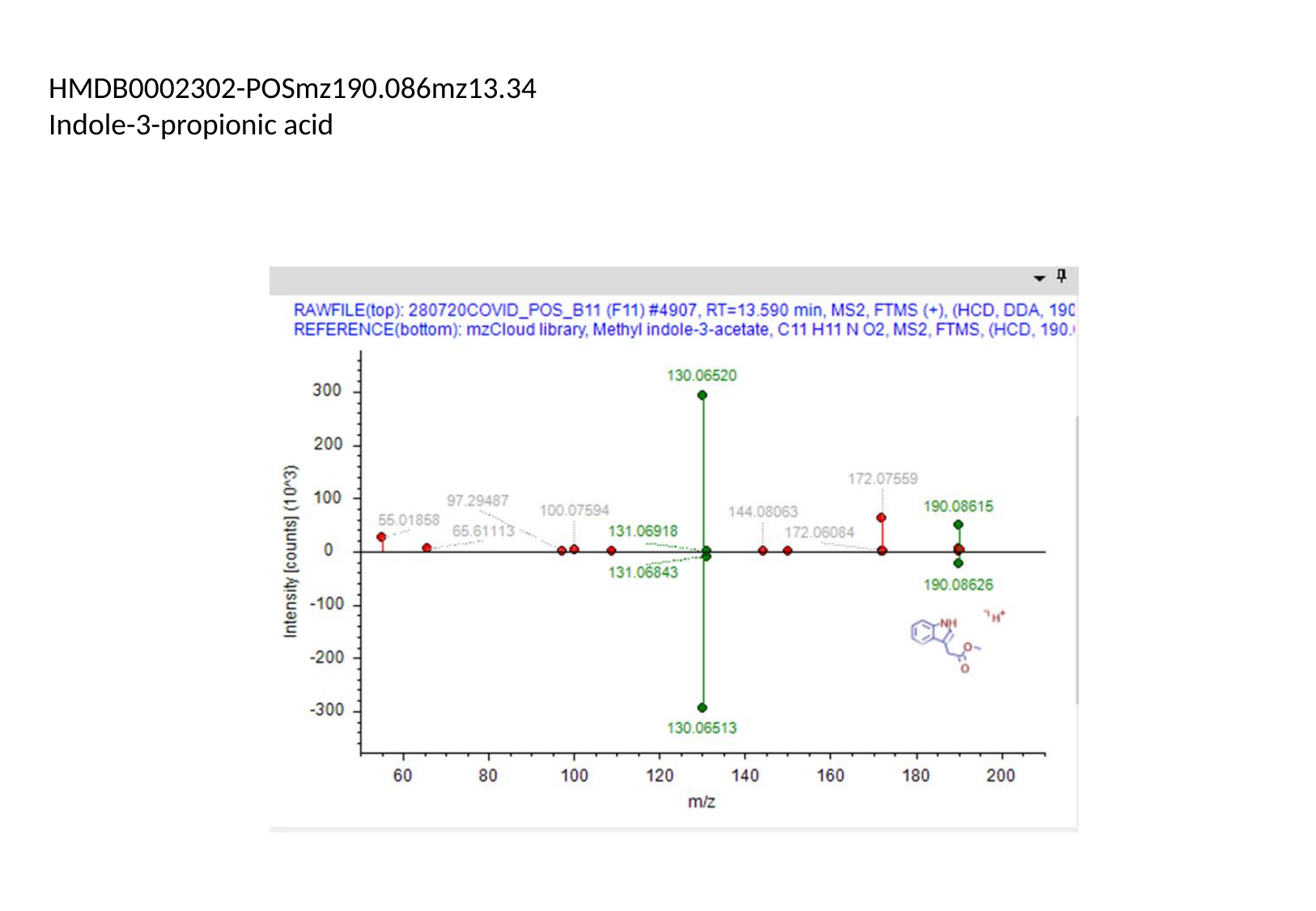

HMDB0002302-POSmz190.086mz13.34
Indole-3-propionic acid

## Slide 30
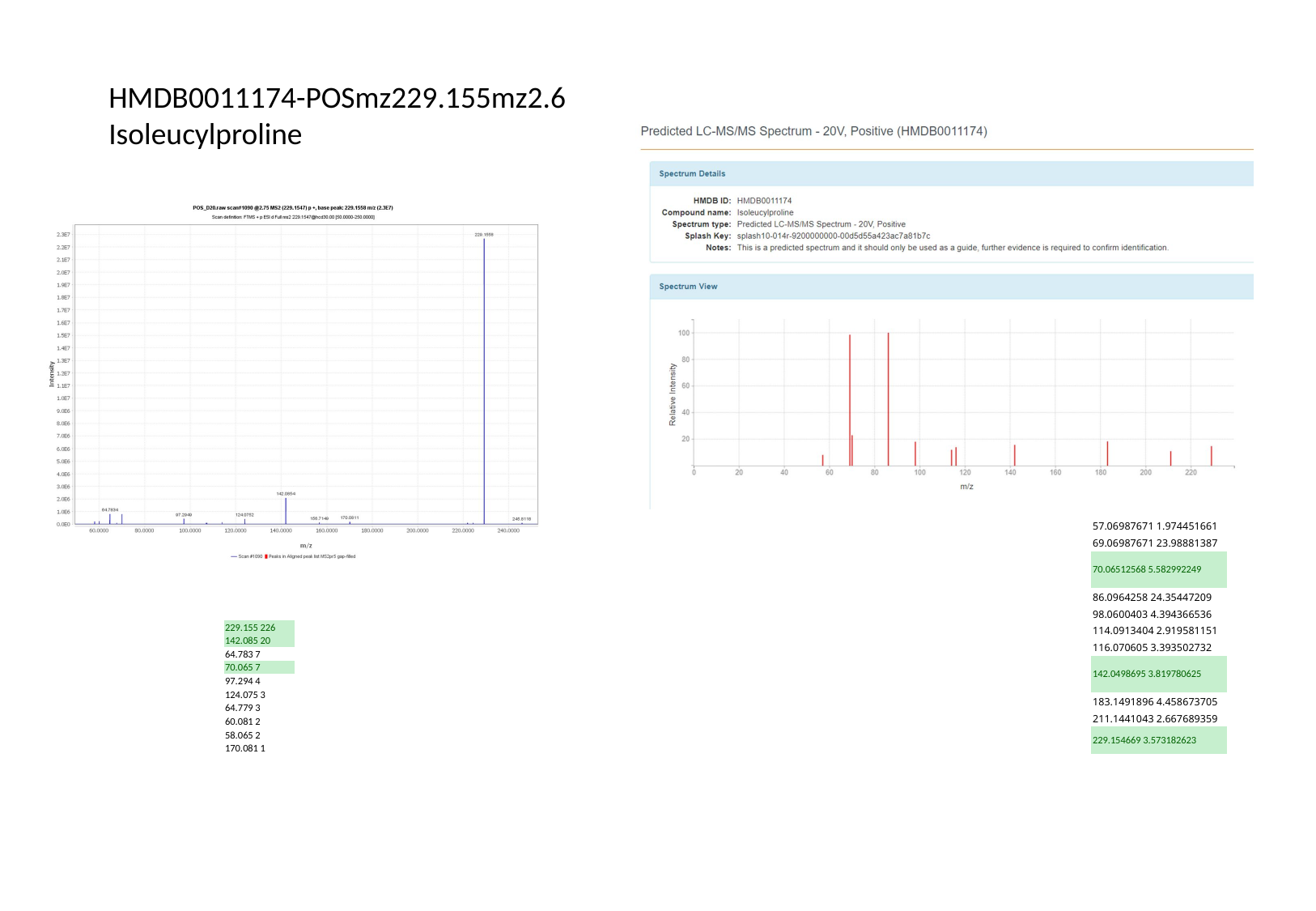

HMDB0011174-POSmz229.155mz2.6
Isoleucylproline
| 57.06987671 1.974451661 |
| --- |
| 69.06987671 23.98881387 |
| 70.06512568 5.582992249 |
| 86.0964258 24.35447209 |
| 98.0600403 4.394366536 |
| 114.0913404 2.919581151 |
| 116.070605 3.393502732 |
| 142.0498695 3.819780625 |
| 183.1491896 4.458673705 |
| 211.1441043 2.667689359 |
| 229.154669 3.573182623 |
| 229.155 226 |
| --- |
| 142.085 20 |
| 64.783 7 |
| 70.065 7 |
| 97.294 4 |
| 124.075 3 |
| 64.779 3 |
| 60.081 2 |
| 58.065 2 |
| 170.081 1 |

## Slide 31
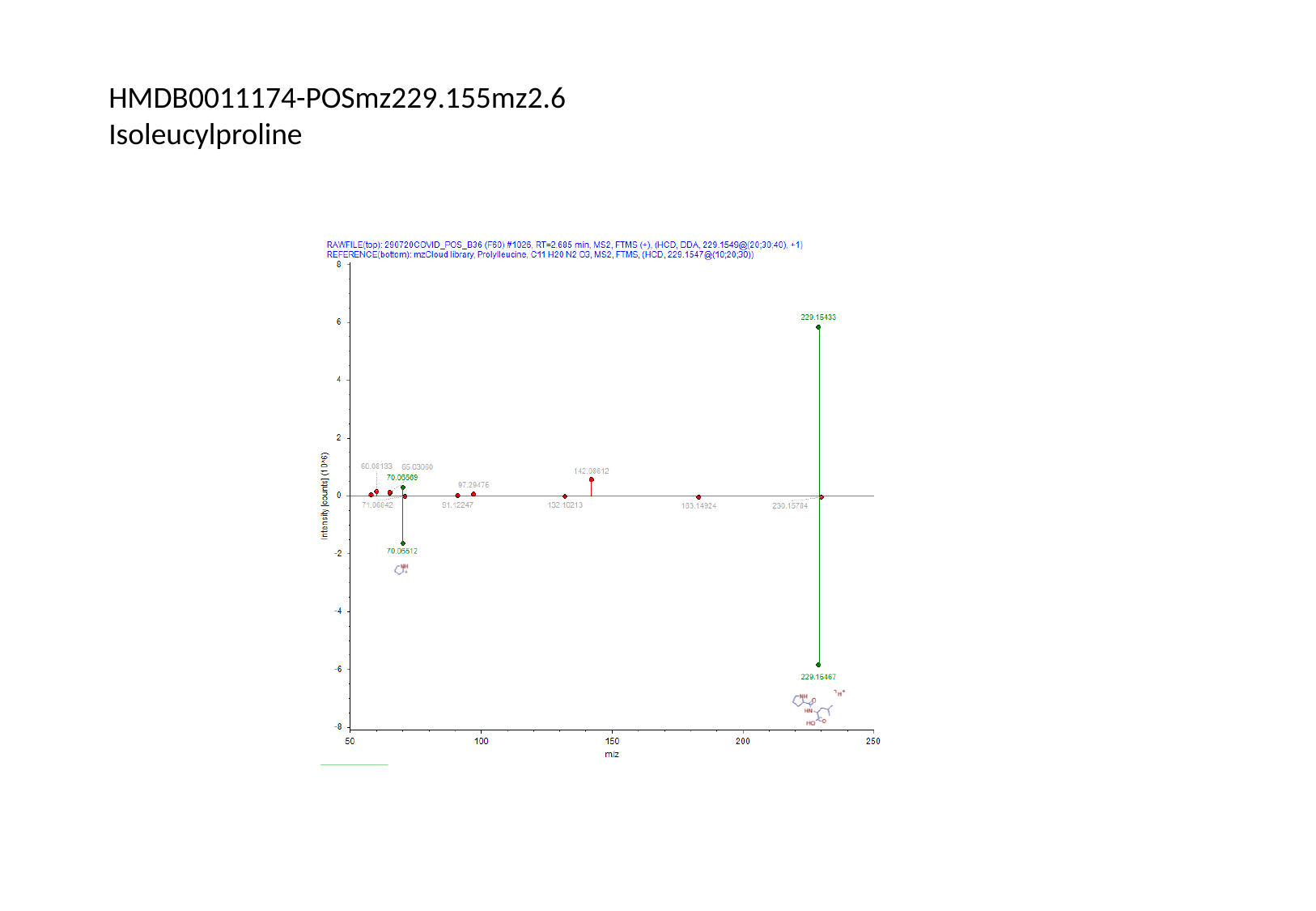

HMDB0011174-POSmz229.155mz2.6
Isoleucylproline

## Slide 32
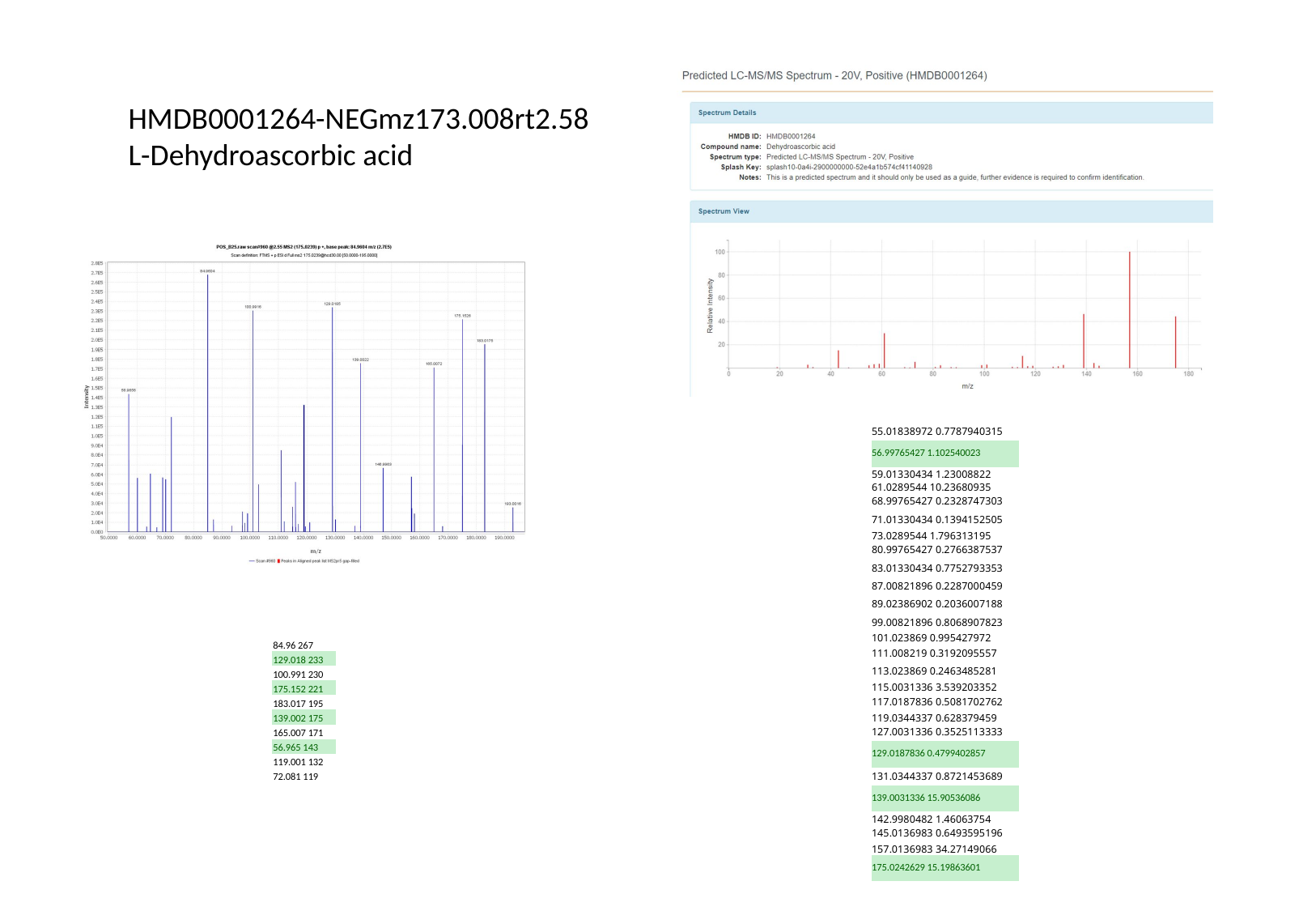

HMDB0001264-NEGmz173.008rt2.58
L-Dehydroascorbic acid
| 55.01838972 0.7787940315 |
| --- |
| 56.99765427 1.102540023 |
| 59.01330434 1.23008822 |
| 61.0289544 10.23680935 |
| 68.99765427 0.2328747303 |
| 71.01330434 0.1394152505 |
| 73.0289544 1.796313195 |
| 80.99765427 0.2766387537 |
| 83.01330434 0.7752793353 |
| 87.00821896 0.2287000459 |
| 89.02386902 0.2036007188 |
| 99.00821896 0.8068907823 |
| 101.023869 0.995427972 |
| 111.008219 0.3192095557 |
| 113.023869 0.2463485281 |
| 115.0031336 3.539203352 |
| 117.0187836 0.5081702762 |
| 119.0344337 0.628379459 |
| 127.0031336 0.3525113333 |
| 129.0187836 0.4799402857 |
| 131.0344337 0.8721453689 |
| 139.0031336 15.90536086 |
| 142.9980482 1.46063754 |
| 145.0136983 0.6493595196 |
| 157.0136983 34.27149066 |
| 175.0242629 15.19863601 |
| 84.96 267 |
| --- |
| 129.018 233 |
| 100.991 230 |
| 175.152 221 |
| 183.017 195 |
| 139.002 175 |
| 165.007 171 |
| 56.965 143 |
| 119.001 132 |
| 72.081 119 |

## Slide 33
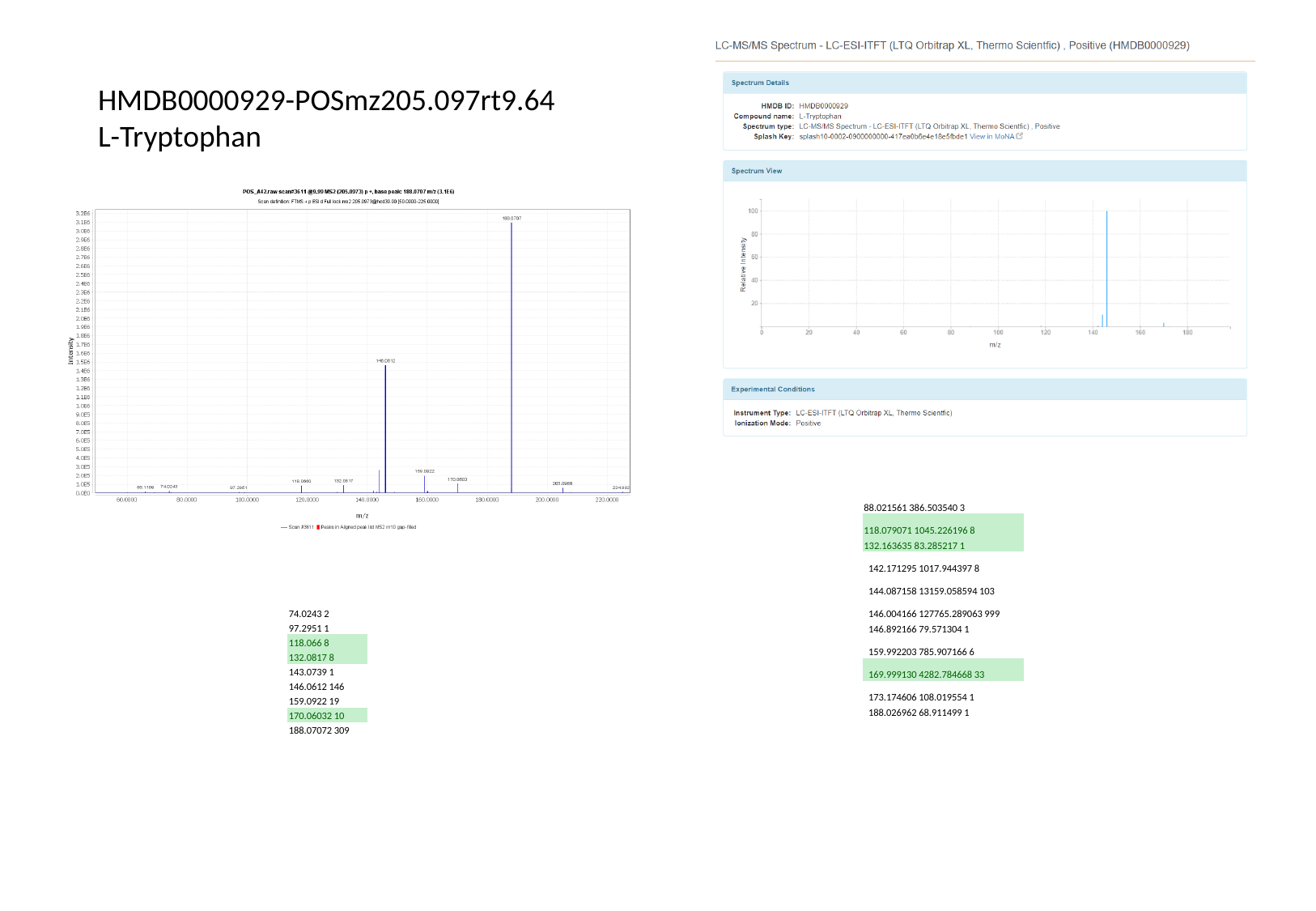

HMDB0000929-POSmz205.097rt9.64
L-Tryptophan
| 88.021561 386.503540 3 |
| --- |
| 118.079071 1045.226196 8 |
| 132.163635 83.285217 1 |
| 142.171295 1017.944397 8 |
| 144.087158 13159.058594 103 |
| 146.004166 127765.289063 999 |
| 146.892166 79.571304 1 |
| 159.992203 785.907166 6 |
| 169.999130 4282.784668 33 |
| 173.174606 108.019554 1 |
| 188.026962 68.911499 1 |
| 74.0243 2 |
| --- |
| 97.2951 1 |
| 118.066 8 |
| 132.0817 8 |
| 143.0739 1 |
| 146.0612 146 |
| 159.0922 19 |
| 170.06032 10 |
| 188.07072 309 |

## Slide 34
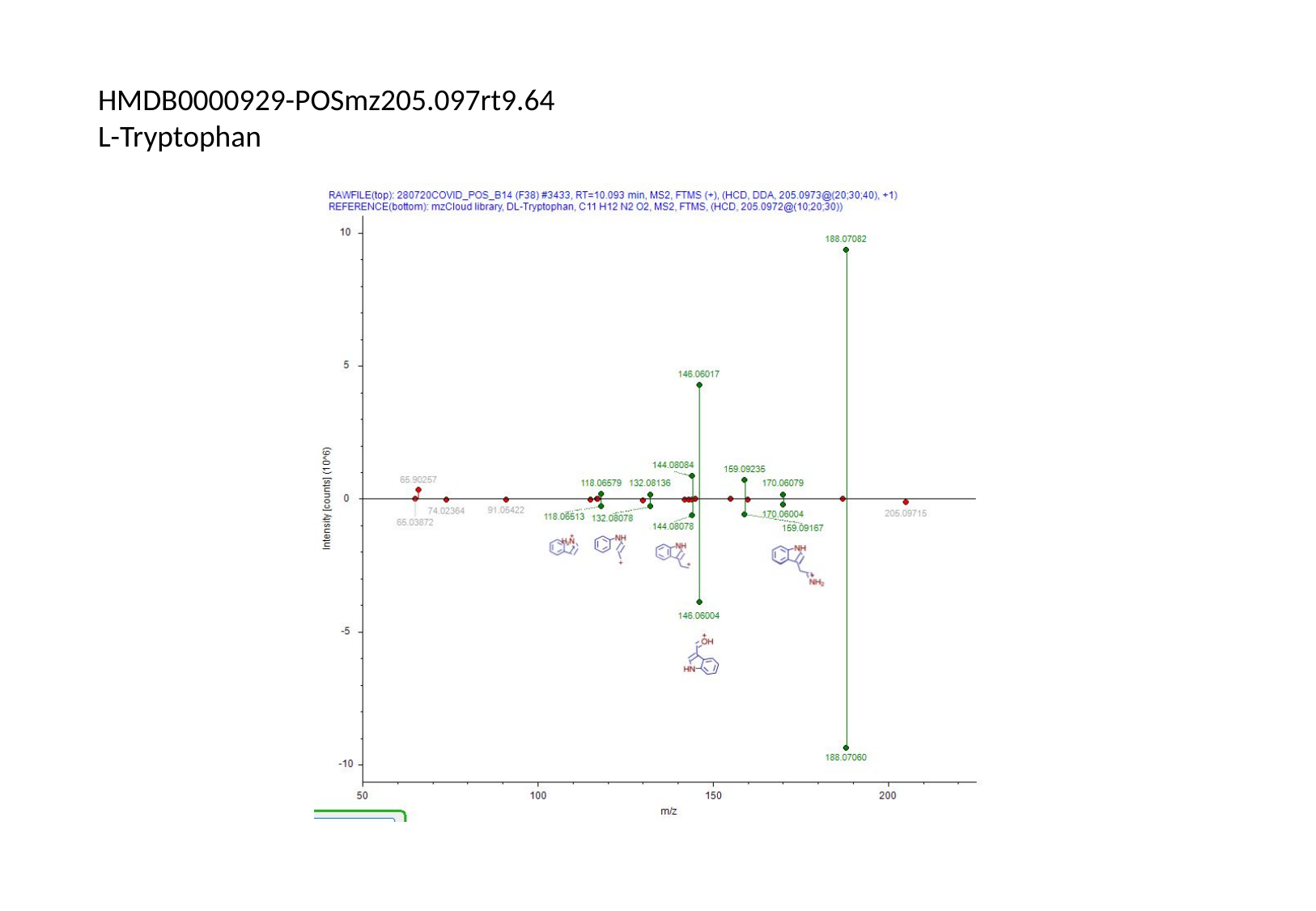

HMDB0000929-POSmz205.097rt9.64
L-Tryptophan

## Slide 35
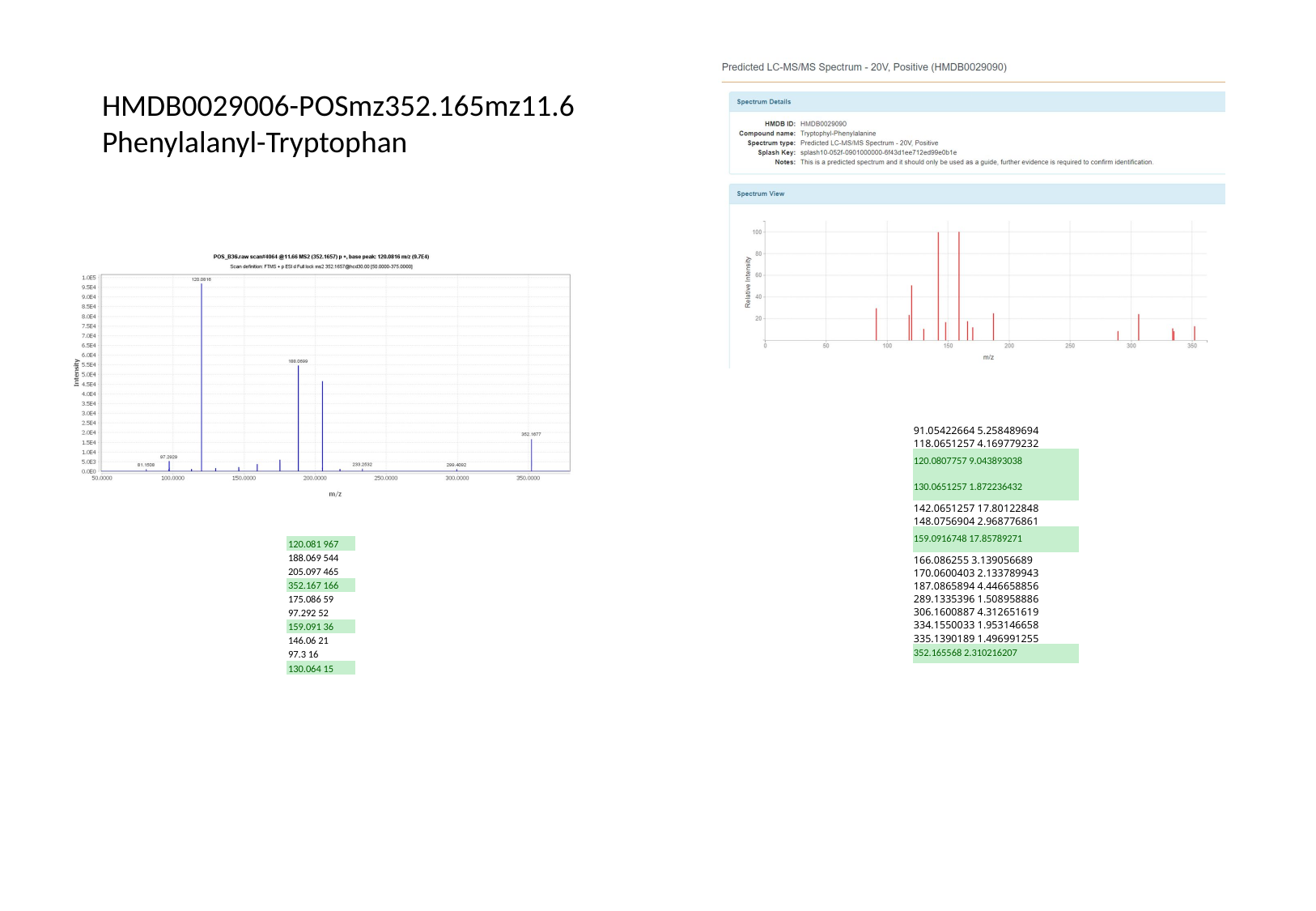

HMDB0029006-POSmz352.165mz11.6
Phenylalanyl-Tryptophan
| 91.05422664 5.258489694 |
| --- |
| 118.0651257 4.169779232 |
| 120.0807757 9.043893038 |
| 130.0651257 1.872236432 |
| 142.0651257 17.80122848 |
| 148.0756904 2.968776861 |
| 159.0916748 17.85789271 |
| 166.086255 3.139056689 |
| 170.0600403 2.133789943 |
| 187.0865894 4.446658856 |
| 289.1335396 1.508958886 |
| 306.1600887 4.312651619 |
| 334.1550033 1.953146658 |
| 335.1390189 1.496991255 |
| 352.165568 2.310216207 |
| 120.081 967 |
| --- |
| 188.069 544 |
| 205.097 465 |
| 352.167 166 |
| 175.086 59 |
| 97.292 52 |
| 159.091 36 |
| 146.06 21 |
| 97.3 16 |
| 130.064 15 |

## Slide 36
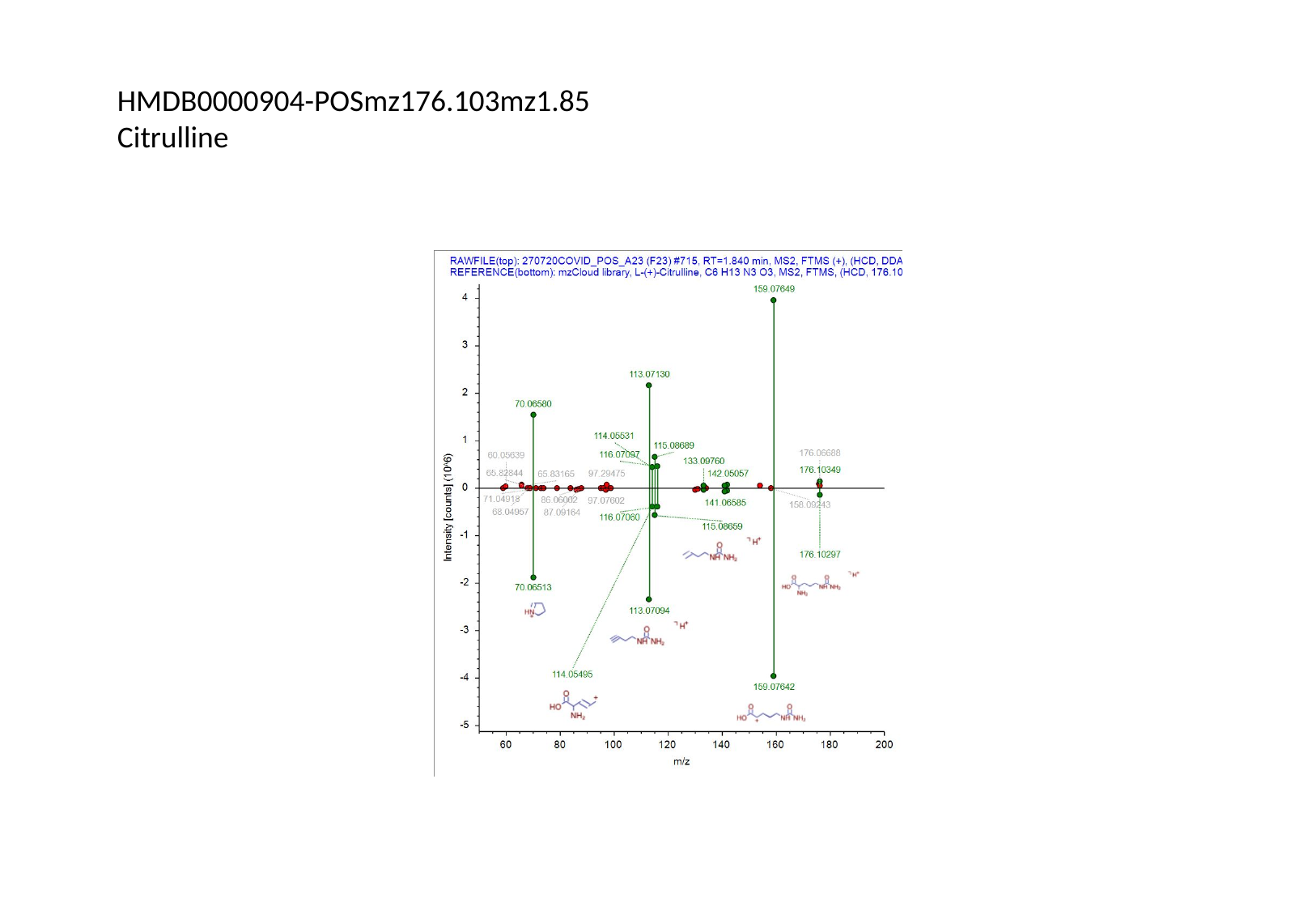

HMDB0000904-POSmz176.103mz1.85
Citrulline

## Slide 37
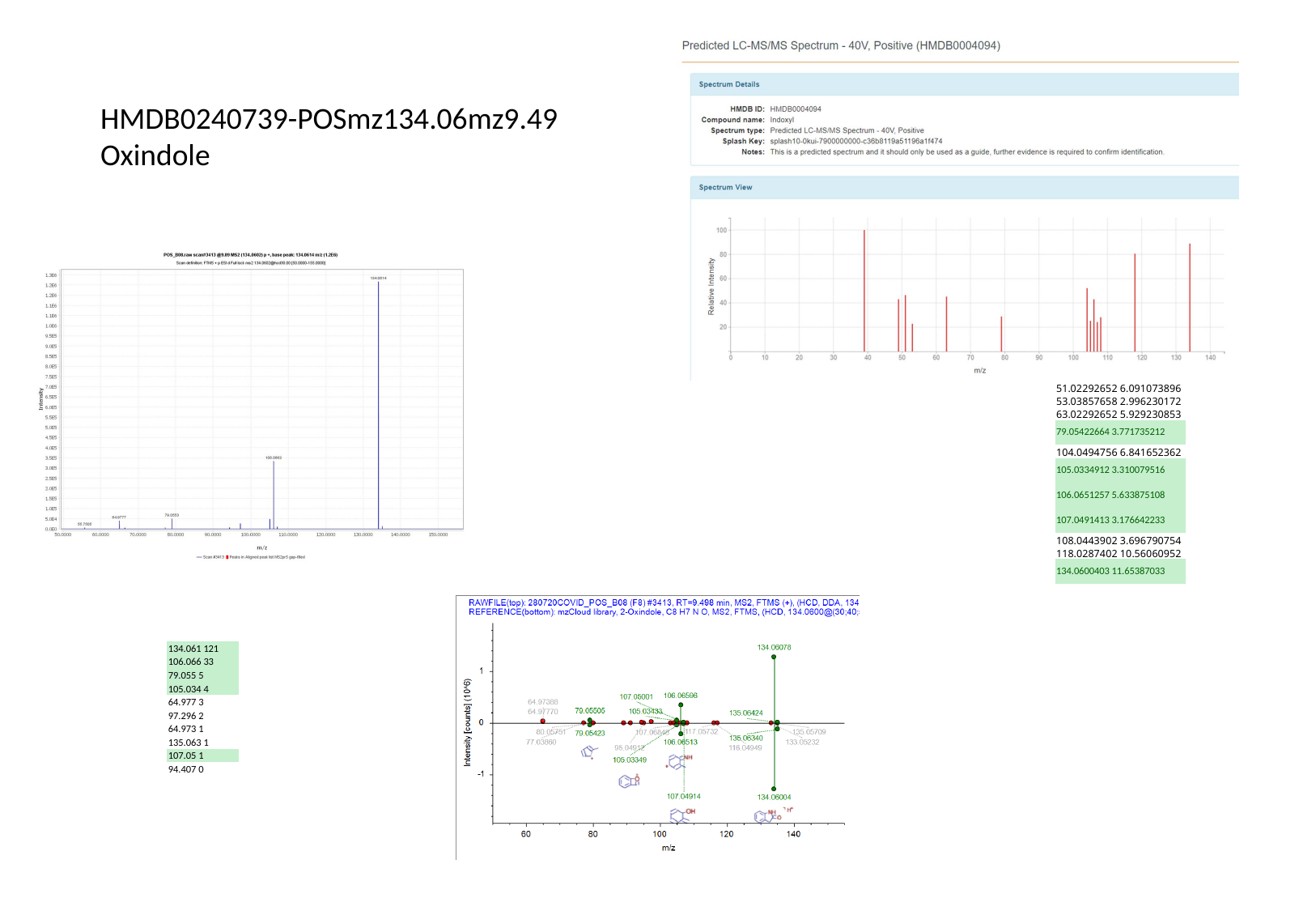

HMDB0240739-POSmz134.06mz9.49
Oxindole
| 51.02292652 6.091073896 |
| --- |
| 53.03857658 2.996230172 |
| 63.02292652 5.929230853 |
| 79.05422664 3.771735212 |
| 104.0494756 6.841652362 |
| 105.0334912 3.310079516 |
| 106.0651257 5.633875108 |
| 107.0491413 3.176642233 |
| 108.0443902 3.696790754 |
| 118.0287402 10.56060952 |
| 134.0600403 11.65387033 |
| 134.061 121 |
| --- |
| 106.066 33 |
| 79.055 5 |
| 105.034 4 |
| 64.977 3 |
| 97.296 2 |
| 64.973 1 |
| 135.063 1 |
| 107.05 1 |
| 94.407 0 |

## Slide 38
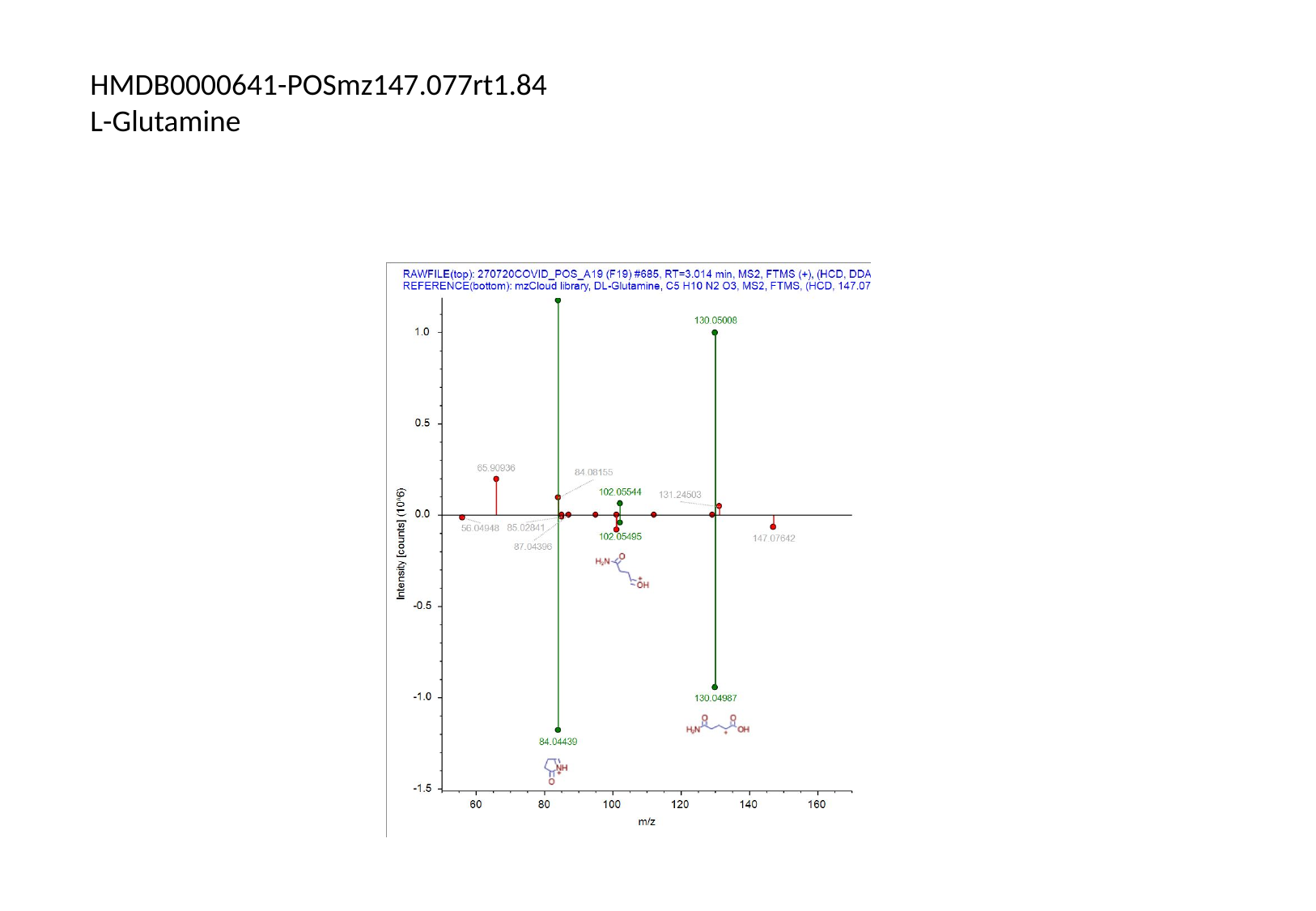

HMDB0000641-POSmz147.077rt1.84
L-Glutamine

## Slide 39
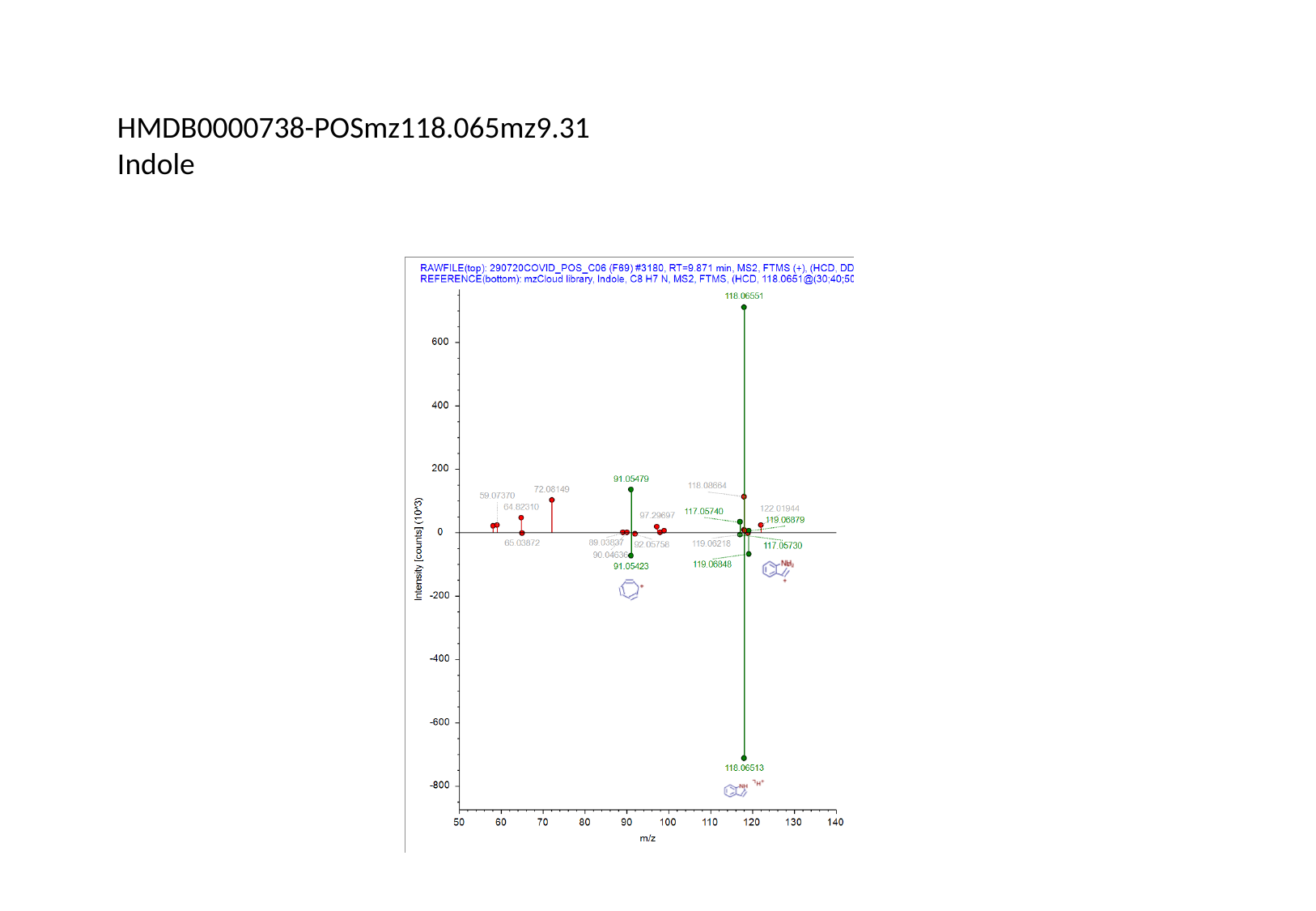

HMDB0000738-POSmz118.065mz9.31
Indole

## Slide 40
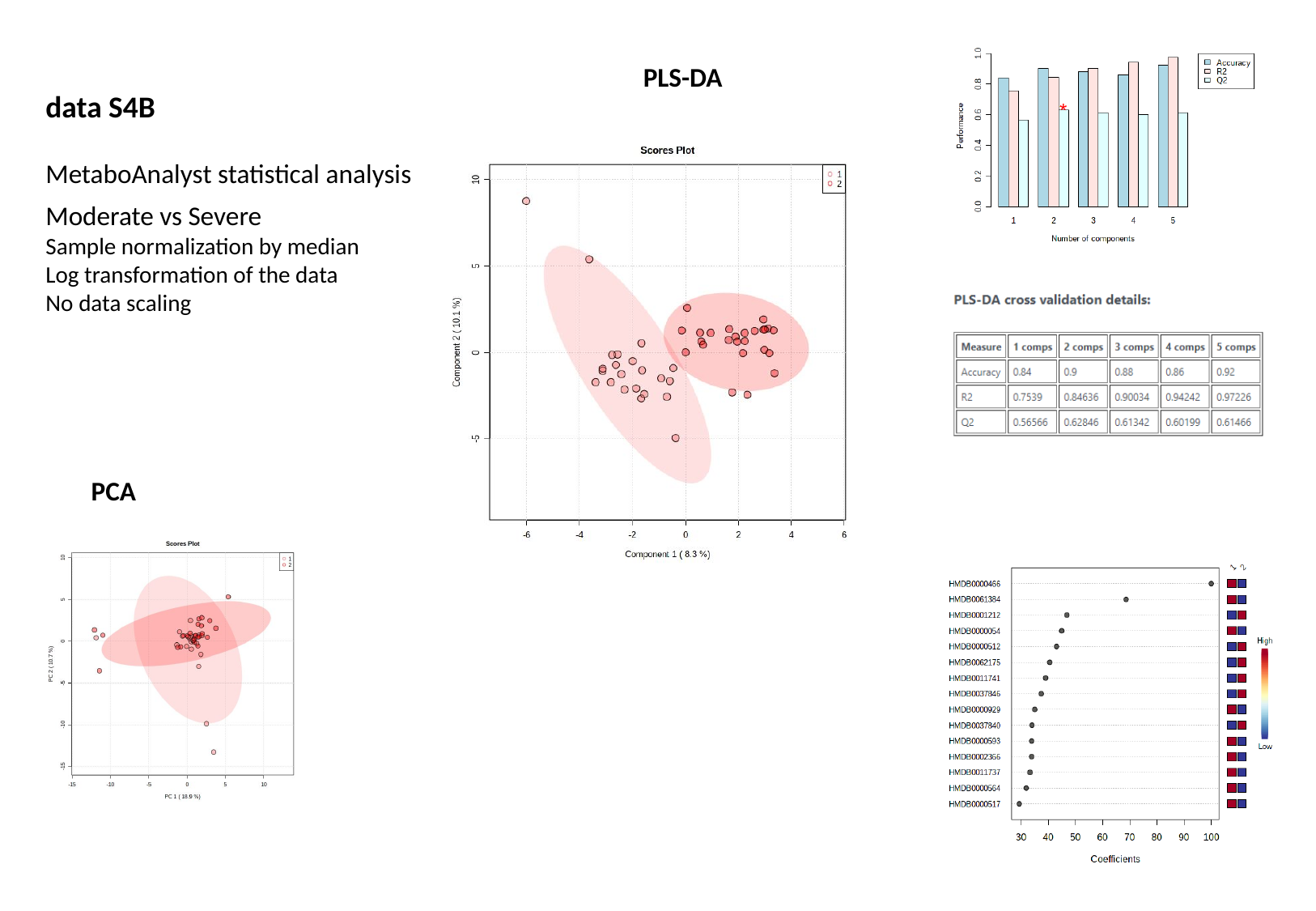

PLS-DA
data S4B
MetaboAnalyst statistical analysis
Moderate vs Severe
Sample normalization by median
Log transformation of the data
No data scaling
PCA

## Slide 41
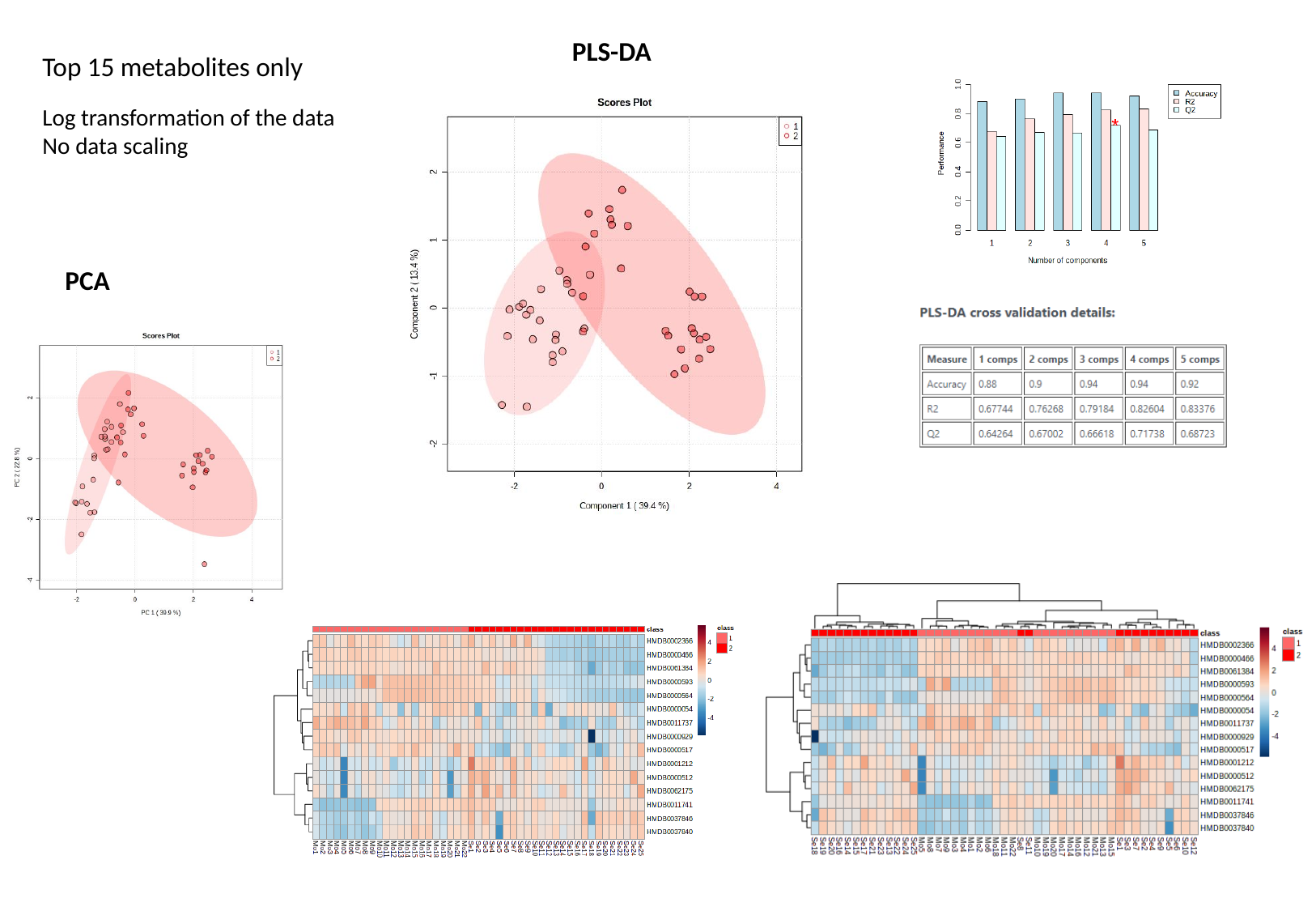

PLS-DA
Top 15 metabolites only
Log transformation of the data
No data scaling
PCA

## Slide 42
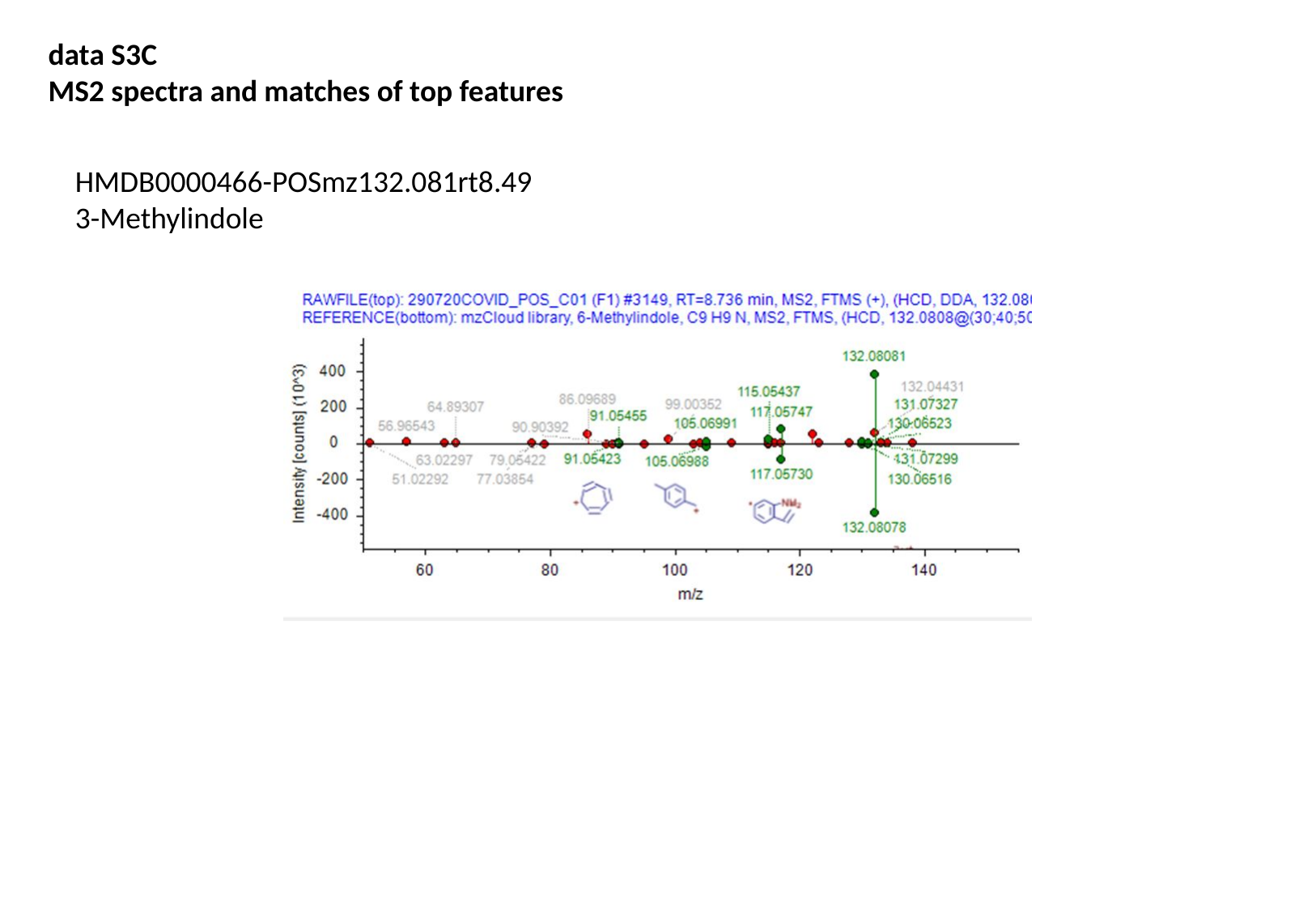

data S3C
MS2 spectra and matches of top features
HMDB0000466-POSmz132.081rt8.49
3-Methylindole

## Slide 43
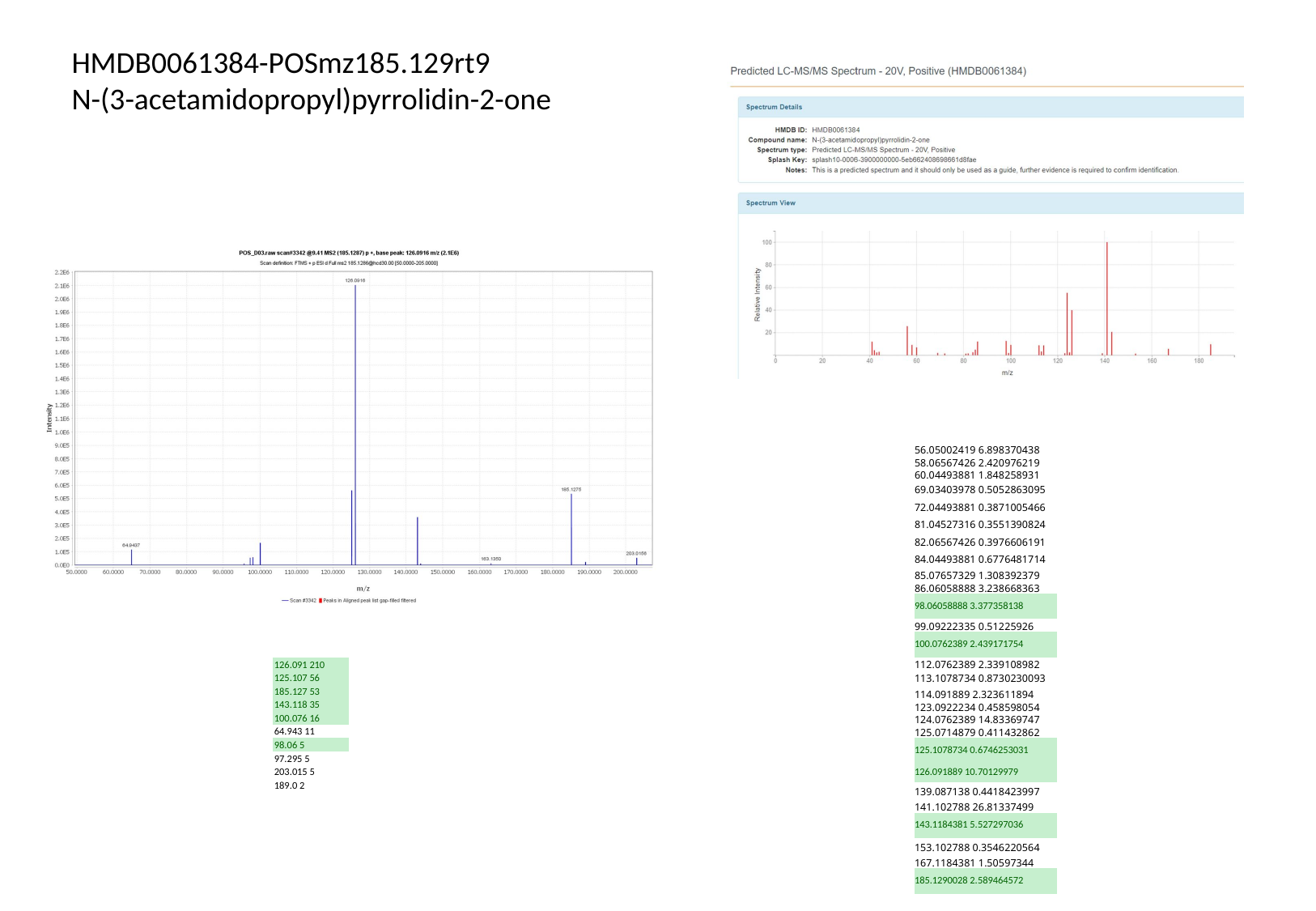

HMDB0061384-POSmz185.129rt9
N-(3-acetamidopropyl)pyrrolidin-2-one
| 56.05002419 6.898370438 |
| --- |
| 58.06567426 2.420976219 |
| 60.04493881 1.848258931 |
| 69.03403978 0.5052863095 |
| 72.04493881 0.3871005466 |
| 81.04527316 0.3551390824 |
| 82.06567426 0.3976606191 |
| 84.04493881 0.6776481714 |
| 85.07657329 1.308392379 |
| 86.06058888 3.238668363 |
| 98.06058888 3.377358138 |
| 99.09222335 0.51225926 |
| 100.0762389 2.439171754 |
| 112.0762389 2.339108982 |
| 113.1078734 0.8730230093 |
| 114.091889 2.323611894 |
| 123.0922234 0.458598054 |
| 124.0762389 14.83369747 |
| 125.0714879 0.411432862 |
| 125.1078734 0.6746253031 |
| 126.091889 10.70129979 |
| 139.087138 0.4418423997 |
| 141.102788 26.81337499 |
| 143.1184381 5.527297036 |
| 153.102788 0.3546220564 |
| 167.1184381 1.50597344 |
| 185.1290028 2.589464572 |
| 126.091 210 |
| --- |
| 125.107 56 |
| 185.127 53 |
| 143.118 35 |
| 100.076 16 |
| 64.943 11 |
| 98.06 5 |
| 97.295 5 |
| 203.015 5 |
| 189.0 2 |

## Slide 44
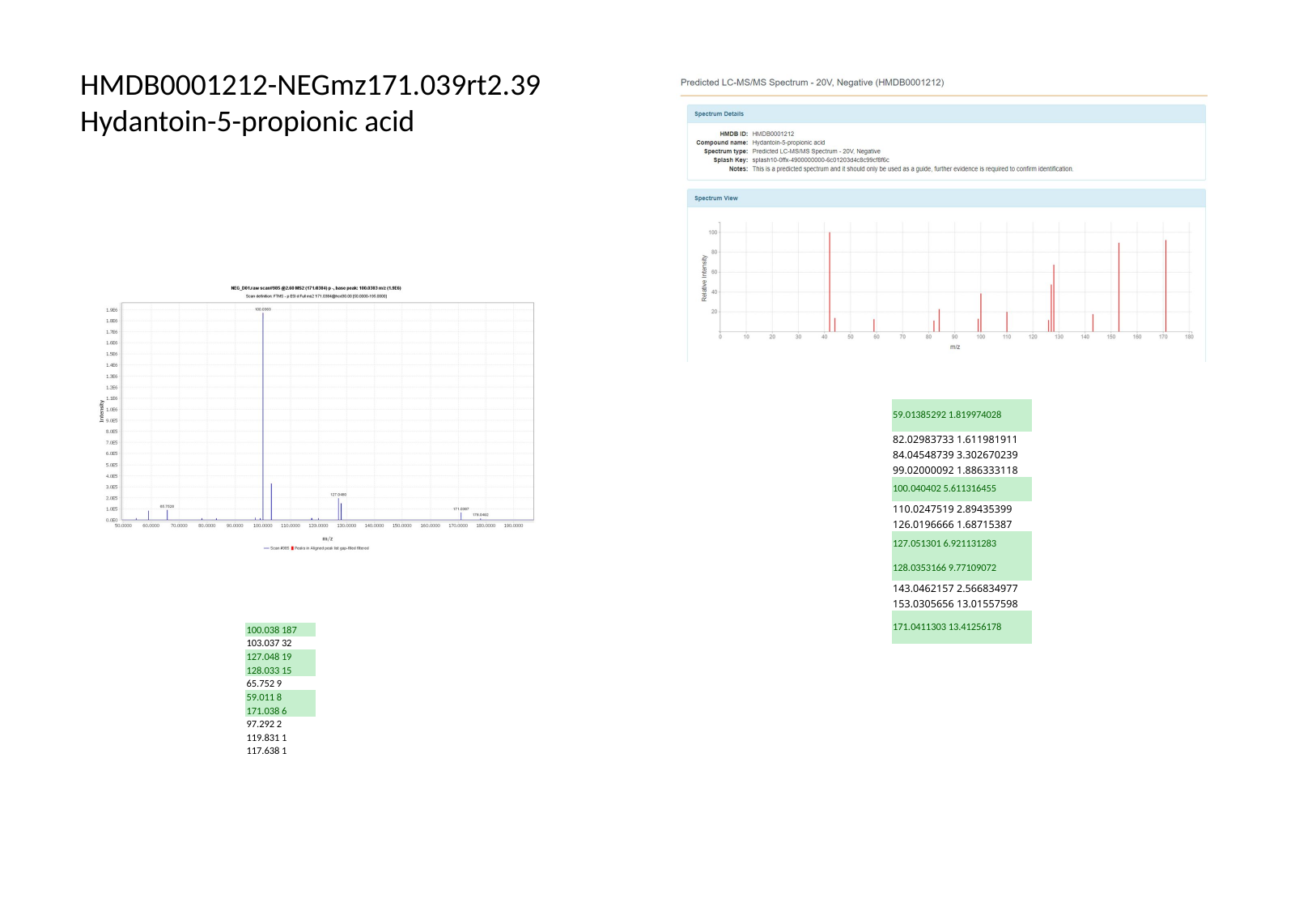

HMDB0001212-NEGmz171.039rt2.39
Hydantoin-5-propionic acid
| 59.01385292 1.819974028 |
| --- |
| 82.02983733 1.611981911 |
| 84.04548739 3.302670239 |
| 99.02000092 1.886333118 |
| 100.040402 5.611316455 |
| 110.0247519 2.89435399 |
| 126.0196666 1.68715387 |
| 127.051301 6.921131283 |
| 128.0353166 9.77109072 |
| 143.0462157 2.566834977 |
| 153.0305656 13.01557598 |
| 171.0411303 13.41256178 |
| 100.038 187 |
| --- |
| 103.037 32 |
| 127.048 19 |
| 128.033 15 |
| 65.752 9 |
| 59.011 8 |
| 171.038 6 |
| 97.292 2 |
| 119.831 1 |
| 117.638 1 |

## Slide 45
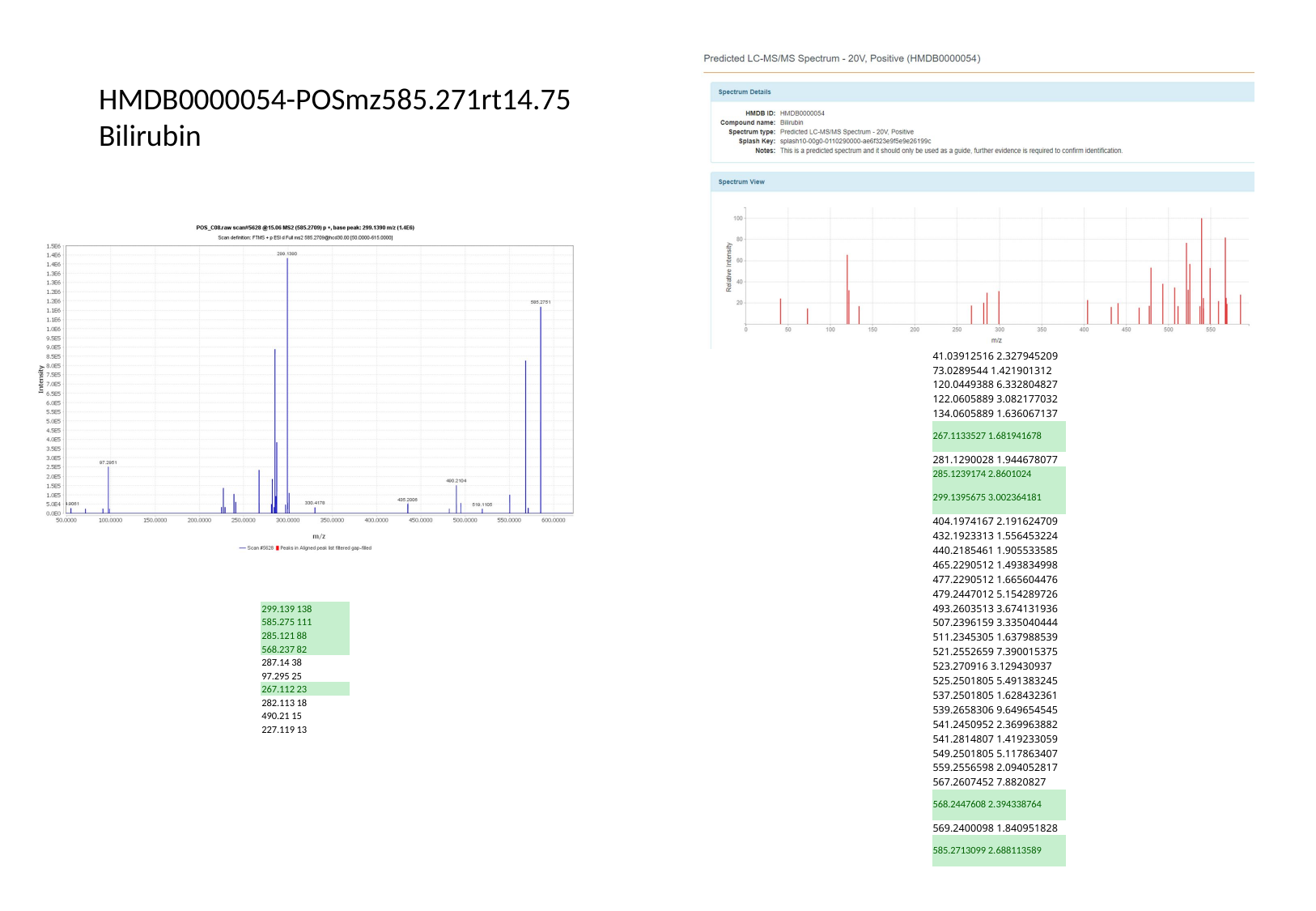

HMDB0000054-POSmz585.271rt14.75
Bilirubin
| 41.03912516 2.327945209 |
| --- |
| 73.0289544 1.421901312 |
| 120.0449388 6.332804827 |
| 122.0605889 3.082177032 |
| 134.0605889 1.636067137 |
| 267.1133527 1.681941678 |
| 281.1290028 1.944678077 |
| 285.1239174 2.8601024 |
| 299.1395675 3.002364181 |
| 404.1974167 2.191624709 |
| 432.1923313 1.556453224 |
| 440.2185461 1.905533585 |
| 465.2290512 1.493834998 |
| 477.2290512 1.665604476 |
| 479.2447012 5.154289726 |
| 493.2603513 3.674131936 |
| 507.2396159 3.335040444 |
| 511.2345305 1.637988539 |
| 521.2552659 7.390015375 |
| 523.270916 3.129430937 |
| 525.2501805 5.491383245 |
| 537.2501805 1.628432361 |
| 539.2658306 9.649654545 |
| 541.2450952 2.369963882 |
| 541.2814807 1.419233059 |
| 549.2501805 5.117863407 |
| 559.2556598 2.094052817 |
| 567.2607452 7.8820827 |
| 568.2447608 2.394338764 |
| 569.2400098 1.840951828 |
| 585.2713099 2.688113589 |
| 299.139 138 |
| --- |
| 585.275 111 |
| 285.121 88 |
| 568.237 82 |
| 287.14 38 |
| 97.295 25 |
| 267.112 23 |
| 282.113 18 |
| 490.21 15 |
| 227.119 13 |

## Slide 46
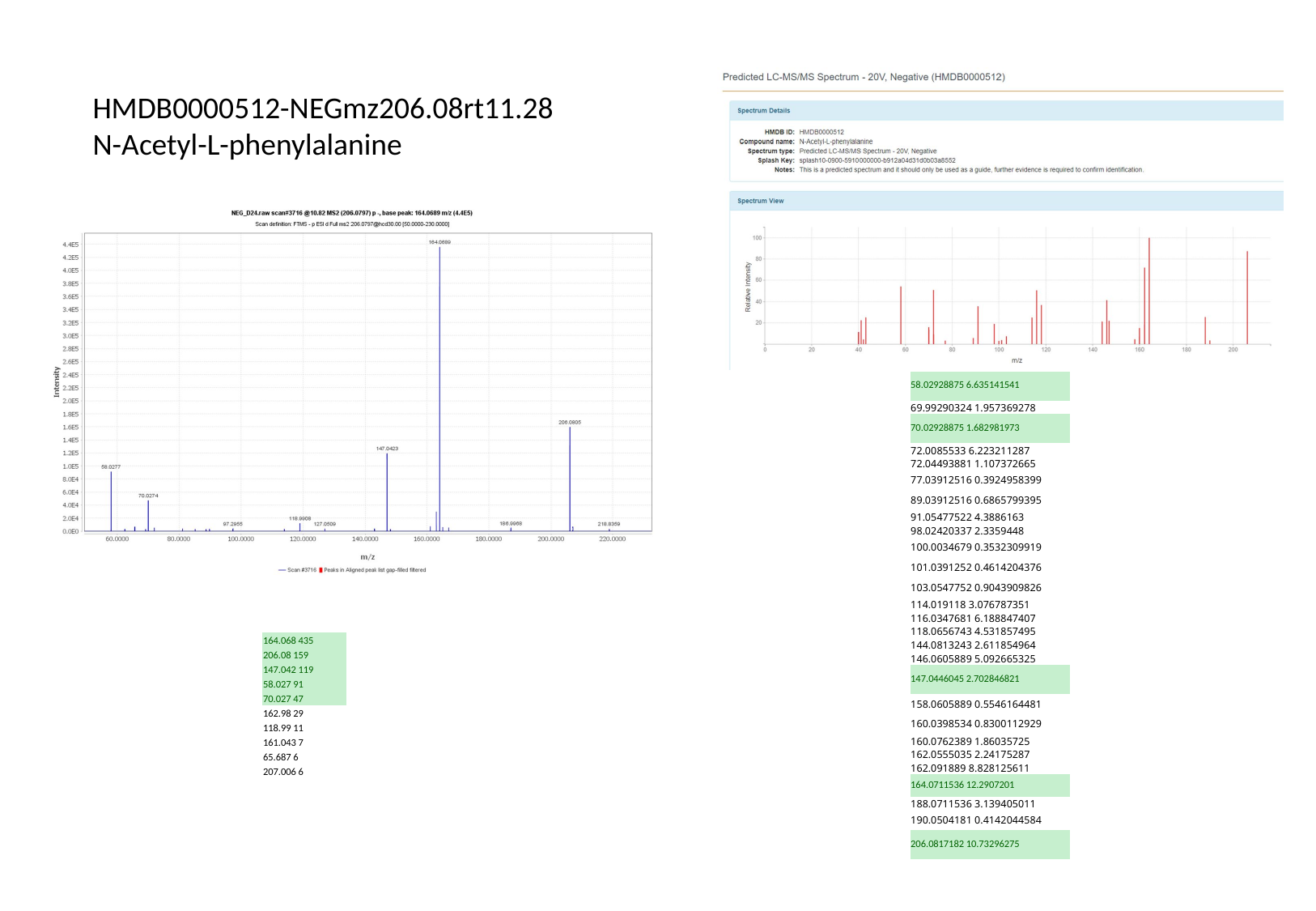

HMDB0000512-NEGmz206.08rt11.28
N-Acetyl-L-phenylalanine
| 58.02928875 6.635141541 |
| --- |
| 69.99290324 1.957369278 |
| 70.02928875 1.682981973 |
| 72.0085533 6.223211287 |
| 72.04493881 1.107372665 |
| 77.03912516 0.3924958399 |
| 89.03912516 0.6865799395 |
| 91.05477522 4.3886163 |
| 98.02420337 2.3359448 |
| 100.0034679 0.3532309919 |
| 101.0391252 0.4614204376 |
| 103.0547752 0.9043909826 |
| 114.019118 3.076787351 |
| 116.0347681 6.188847407 |
| 118.0656743 4.531857495 |
| 144.0813243 2.611854964 |
| 146.0605889 5.092665325 |
| 147.0446045 2.702846821 |
| 158.0605889 0.5546164481 |
| 160.0398534 0.8300112929 |
| 160.0762389 1.86035725 |
| 162.0555035 2.24175287 |
| 162.091889 8.828125611 |
| 164.0711536 12.2907201 |
| 188.0711536 3.139405011 |
| 190.0504181 0.4142044584 |
| 206.0817182 10.73296275 |
| 164.068 435 |
| --- |
| 206.08 159 |
| 147.042 119 |
| 58.027 91 |
| 70.027 47 |
| 162.98 29 |
| 118.99 11 |
| 161.043 7 |
| 65.687 6 |
| 207.006 6 |

## Slide 47
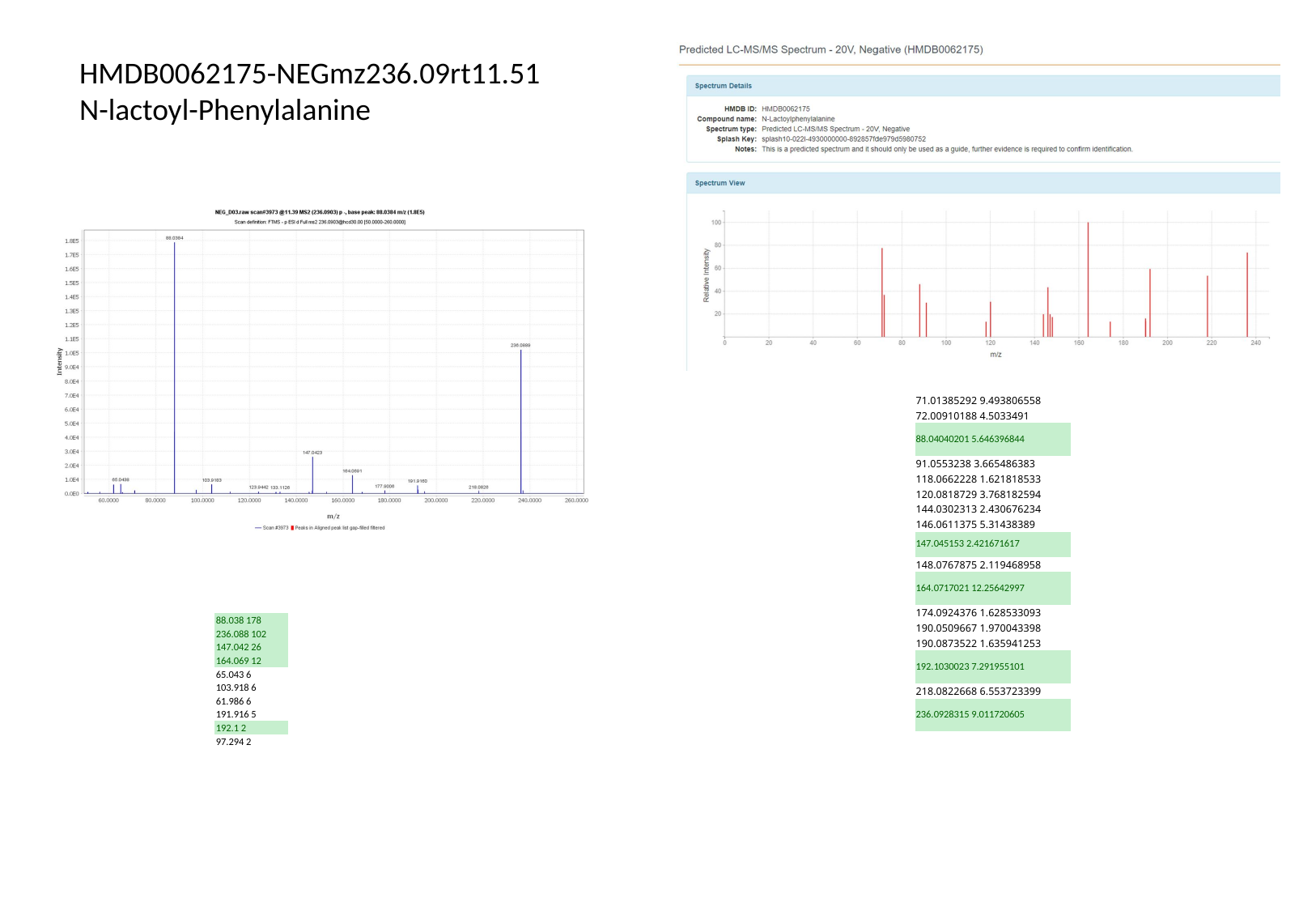

HMDB0062175-NEGmz236.09rt11.51
N-lactoyl-Phenylalanine
| 71.01385292 9.493806558 |
| --- |
| 72.00910188 4.5033491 |
| 88.04040201 5.646396844 |
| 91.0553238 3.665486383 |
| 118.0662228 1.621818533 |
| 120.0818729 3.768182594 |
| 144.0302313 2.430676234 |
| 146.0611375 5.31438389 |
| 147.045153 2.421671617 |
| 148.0767875 2.119468958 |
| 164.0717021 12.25642997 |
| 174.0924376 1.628533093 |
| 190.0509667 1.970043398 |
| 190.0873522 1.635941253 |
| 192.1030023 7.291955101 |
| 218.0822668 6.553723399 |
| 236.0928315 9.011720605 |
| 88.038 178 |
| --- |
| 236.088 102 |
| 147.042 26 |
| 164.069 12 |
| 65.043 6 |
| 103.918 6 |
| 61.986 6 |
| 191.916 5 |
| 192.1 2 |
| 97.294 2 |

## Slide 48
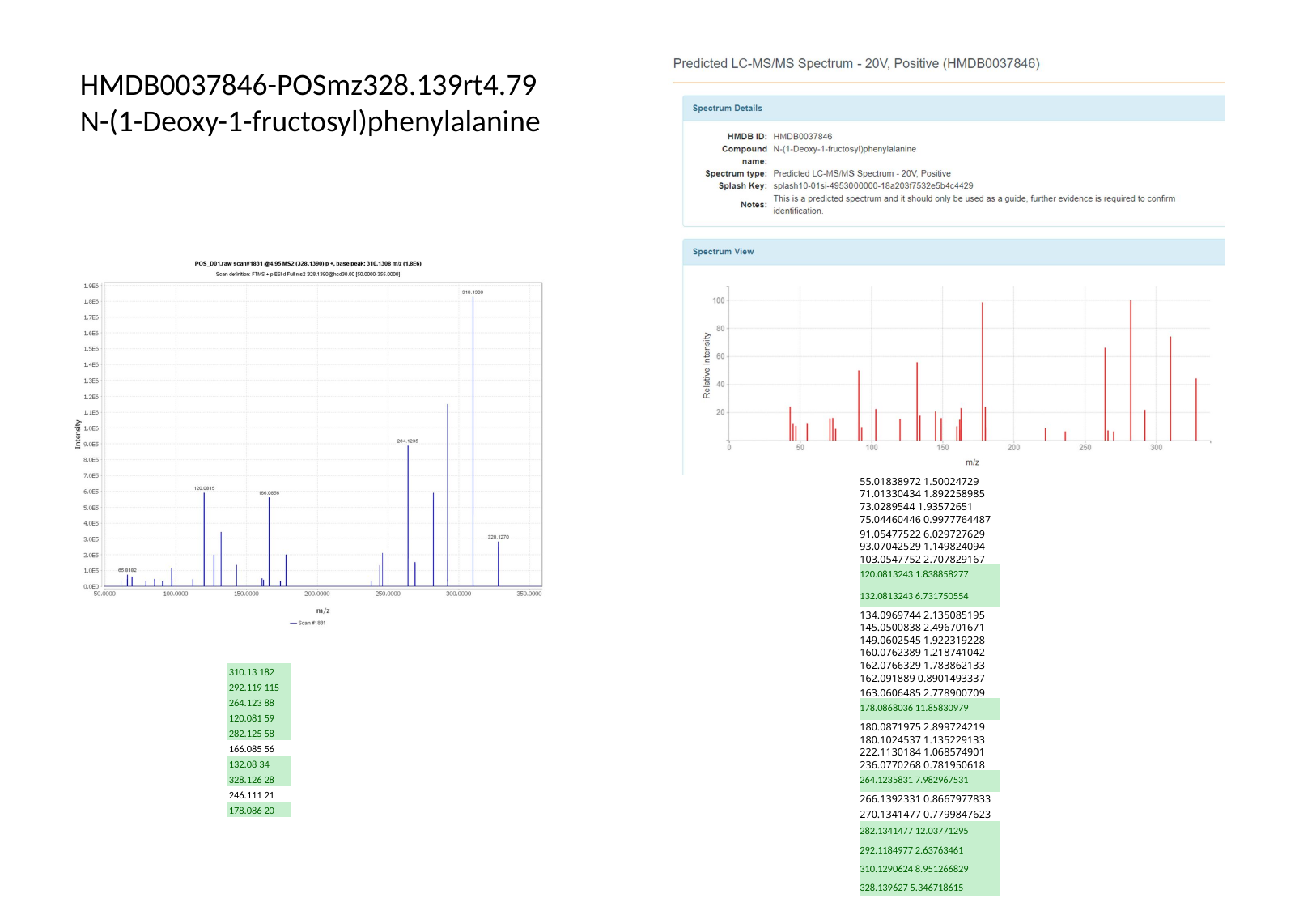

HMDB0037846-POSmz328.139rt4.79
N-(1-Deoxy-1-fructosyl)phenylalanine
| 55.01838972 1.50024729 |
| --- |
| 71.01330434 1.892258985 |
| 73.0289544 1.93572651 |
| 75.04460446 0.9977764487 |
| 91.05477522 6.029727629 |
| 93.07042529 1.149824094 |
| 103.0547752 2.707829167 |
| 120.0813243 1.838858277 |
| 132.0813243 6.731750554 |
| 134.0969744 2.135085195 |
| 145.0500838 2.496701671 |
| 149.0602545 1.922319228 |
| 160.0762389 1.218741042 |
| 162.0766329 1.783862133 |
| 162.091889 0.8901493337 |
| 163.0606485 2.778900709 |
| 178.0868036 11.85830979 |
| 180.0871975 2.899724219 |
| 180.1024537 1.135229133 |
| 222.1130184 1.068574901 |
| 236.0770268 0.781950618 |
| 264.1235831 7.982967531 |
| 266.1392331 0.8667977833 |
| 270.1341477 0.7799847623 |
| 282.1341477 12.03771295 |
| 292.1184977 2.63763461 |
| 310.1290624 8.951266829 |
| 328.139627 5.346718615 |
| 310.13 182 |
| --- |
| 292.119 115 |
| 264.123 88 |
| 120.081 59 |
| 282.125 58 |
| 166.085 56 |
| 132.08 34 |
| 328.126 28 |
| 246.111 21 |
| 178.086 20 |

## Slide 49
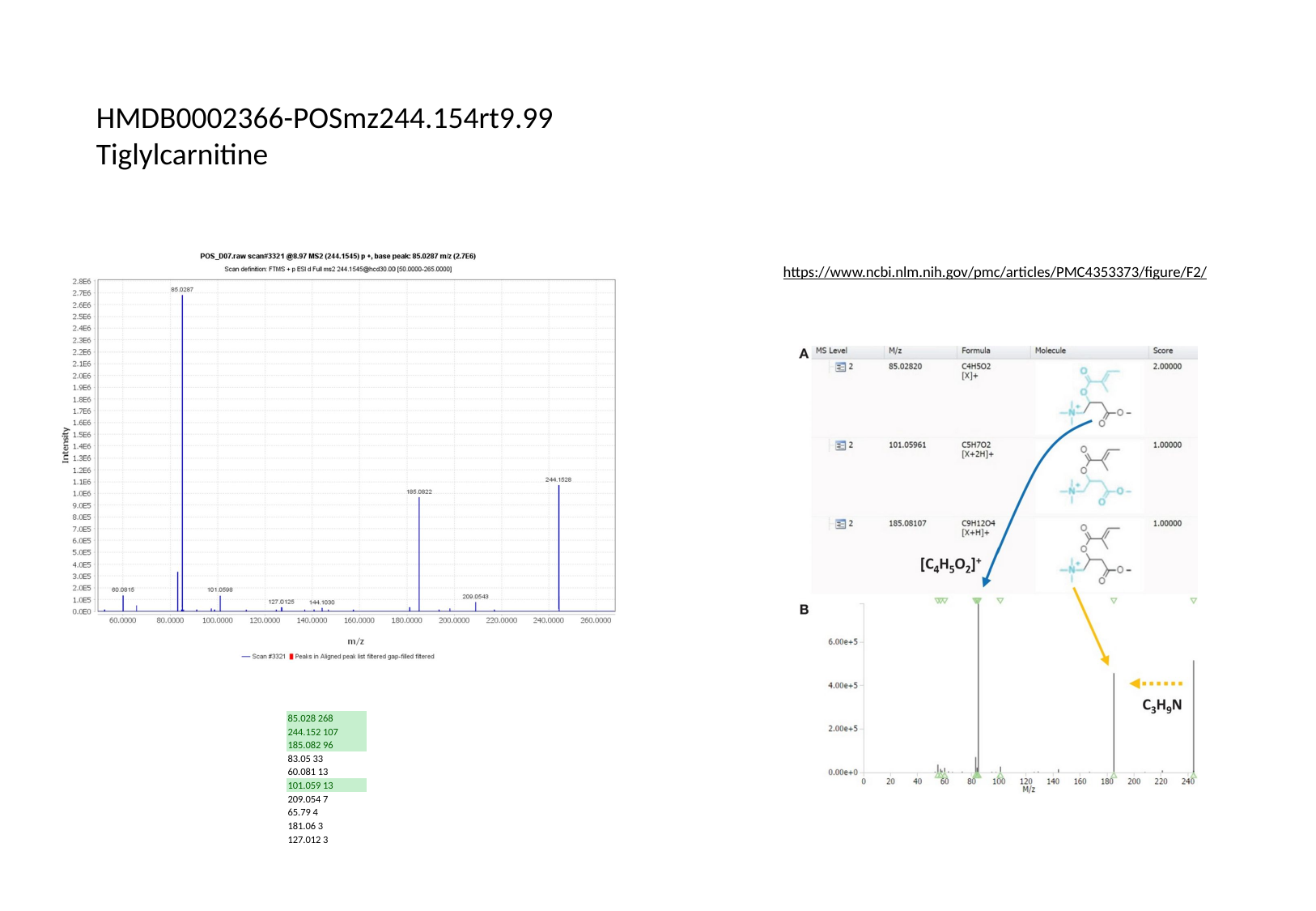

HMDB0002366-POSmz244.154rt9.99
Tiglylcarnitine
https://www.ncbi.nlm.nih.gov/pmc/articles/PMC4353373/figure/F2/
| 85.028 268 |
| --- |
| 244.152 107 |
| 185.082 96 |
| 83.05 33 |
| 60.081 13 |
| 101.059 13 |
| 209.054 7 |
| 65.79 4 |
| 181.06 3 |
| 127.012 3 |

## Slide 50
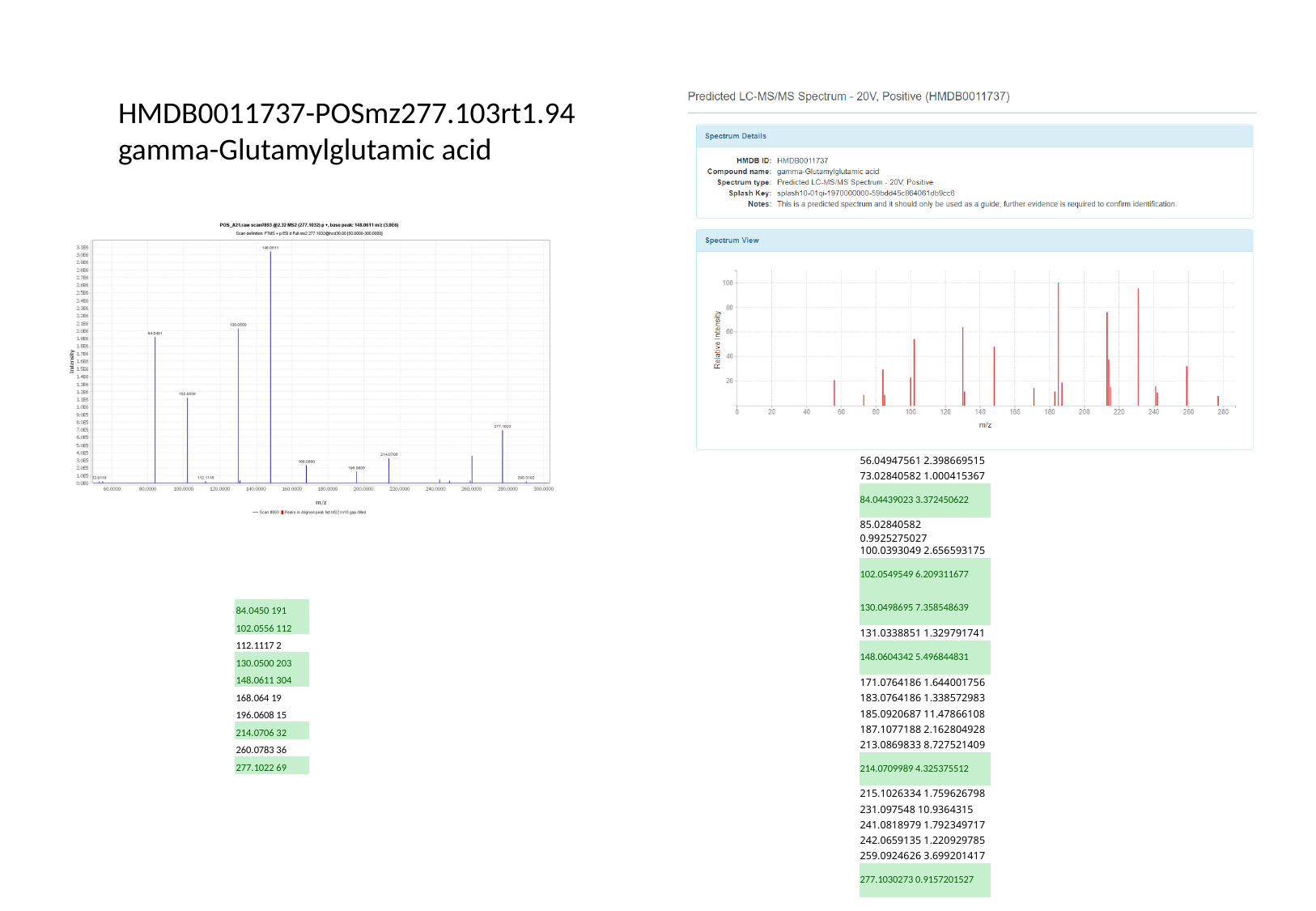

HMDB0011737-POSmz277.103rt1.94
gamma-Glutamylglutamic acid
| 56.04947561 2.398669515 |
| --- |
| 73.02840582 1.000415367 |
| 84.04439023 3.372450622 |
| 85.02840582 0.9925275027 |
| 100.0393049 2.656593175 |
| 102.0549549 6.209311677 |
| 130.0498695 7.358548639 |
| 131.0338851 1.329791741 |
| 148.0604342 5.496844831 |
| 171.0764186 1.644001756 |
| 183.0764186 1.338572983 |
| 185.0920687 11.47866108 |
| 187.1077188 2.162804928 |
| 213.0869833 8.727521409 |
| 214.0709989 4.325375512 |
| 215.1026334 1.759626798 |
| 231.097548 10.9364315 |
| 241.0818979 1.792349717 |
| 242.0659135 1.220929785 |
| 259.0924626 3.699201417 |
| 277.1030273 0.9157201527 |
| 84.0450 191 |
| --- |
| 102.0556 112 |
| 112.1117 2 |
| 130.0500 203 |
| 148.0611 304 |
| 168.064 19 |
| 196.0608 15 |
| 214.0706 32 |
| 260.0783 36 |
| 277.1022 69 |

## Slide 51
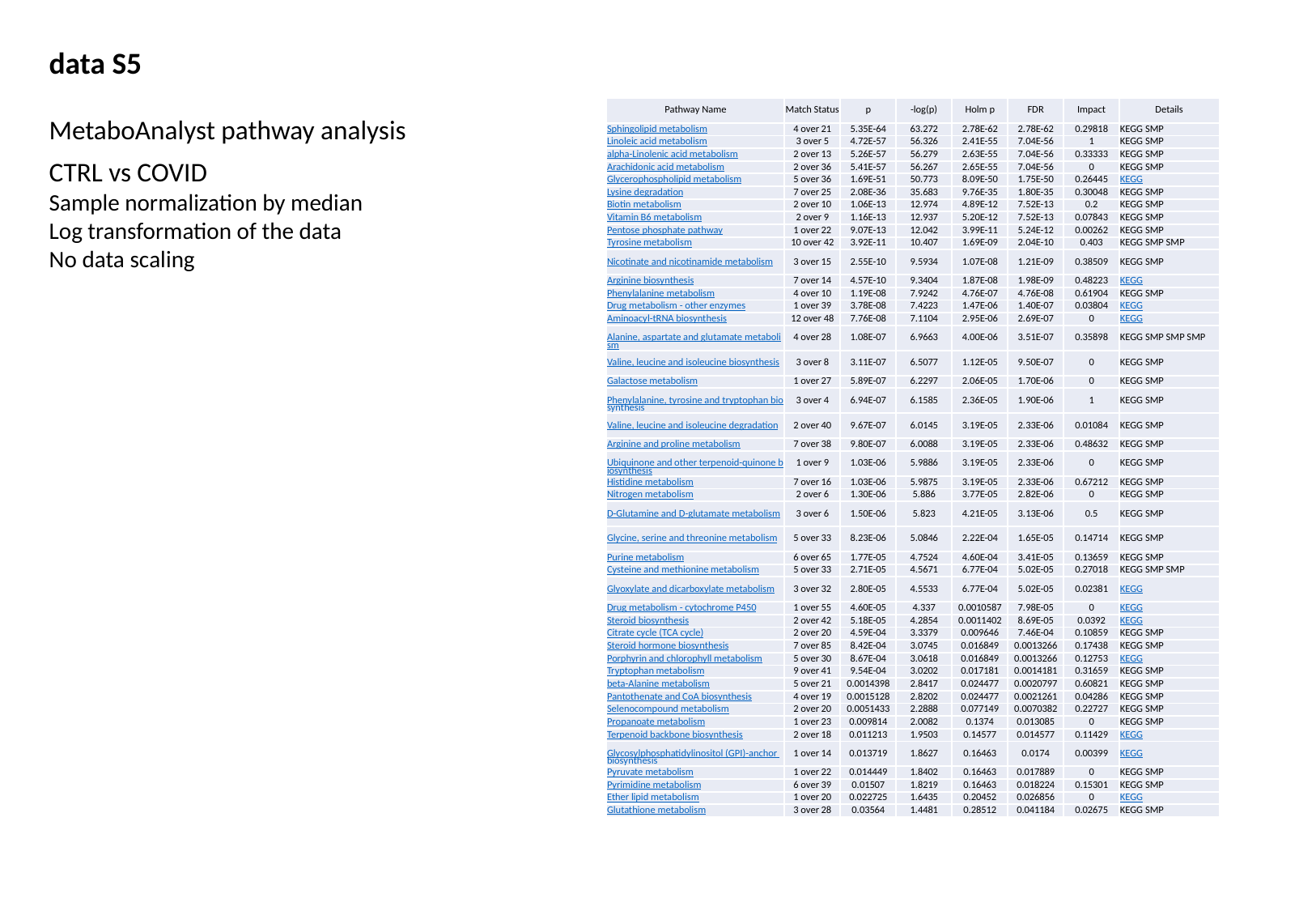

data S5
MetaboAnalyst pathway analysis
CTRL vs COVID
Sample normalization by median
Log transformation of the data
No data scaling
| Pathway Name | Match Status | p | -log(p) | Holm p | FDR | Impact | Details |
| --- | --- | --- | --- | --- | --- | --- | --- |
| Sphingolipid metabolism | 4 over 21 | 5.35E-64 | 63.272 | 2.78E-62 | 2.78E-62 | 0.29818 | KEGG SMP |
| Linoleic acid metabolism | 3 over 5 | 4.72E-57 | 56.326 | 2.41E-55 | 7.04E-56 | 1 | KEGG SMP |
| alpha-Linolenic acid metabolism | 2 over 13 | 5.26E-57 | 56.279 | 2.63E-55 | 7.04E-56 | 0.33333 | KEGG SMP |
| Arachidonic acid metabolism | 2 over 36 | 5.41E-57 | 56.267 | 2.65E-55 | 7.04E-56 | 0 | KEGG SMP |
| Glycerophospholipid metabolism | 5 over 36 | 1.69E-51 | 50.773 | 8.09E-50 | 1.75E-50 | 0.26445 | KEGG |
| Lysine degradation | 7 over 25 | 2.08E-36 | 35.683 | 9.76E-35 | 1.80E-35 | 0.30048 | KEGG SMP |
| Biotin metabolism | 2 over 10 | 1.06E-13 | 12.974 | 4.89E-12 | 7.52E-13 | 0.2 | KEGG SMP |
| Vitamin B6 metabolism | 2 over 9 | 1.16E-13 | 12.937 | 5.20E-12 | 7.52E-13 | 0.07843 | KEGG SMP |
| Pentose phosphate pathway | 1 over 22 | 9.07E-13 | 12.042 | 3.99E-11 | 5.24E-12 | 0.00262 | KEGG SMP |
| Tyrosine metabolism | 10 over 42 | 3.92E-11 | 10.407 | 1.69E-09 | 2.04E-10 | 0.403 | KEGG SMP SMP |
| Nicotinate and nicotinamide metabolism | 3 over 15 | 2.55E-10 | 9.5934 | 1.07E-08 | 1.21E-09 | 0.38509 | KEGG SMP |
| Arginine biosynthesis | 7 over 14 | 4.57E-10 | 9.3404 | 1.87E-08 | 1.98E-09 | 0.48223 | KEGG |
| Phenylalanine metabolism | 4 over 10 | 1.19E-08 | 7.9242 | 4.76E-07 | 4.76E-08 | 0.61904 | KEGG SMP |
| Drug metabolism - other enzymes | 1 over 39 | 3.78E-08 | 7.4223 | 1.47E-06 | 1.40E-07 | 0.03804 | KEGG |
| Aminoacyl-tRNA biosynthesis | 12 over 48 | 7.76E-08 | 7.1104 | 2.95E-06 | 2.69E-07 | 0 | KEGG |
| Alanine, aspartate and glutamate metabolism | 4 over 28 | 1.08E-07 | 6.9663 | 4.00E-06 | 3.51E-07 | 0.35898 | KEGG SMP SMP SMP |
| Valine, leucine and isoleucine biosynthesis | 3 over 8 | 3.11E-07 | 6.5077 | 1.12E-05 | 9.50E-07 | 0 | KEGG SMP |
| Galactose metabolism | 1 over 27 | 5.89E-07 | 6.2297 | 2.06E-05 | 1.70E-06 | 0 | KEGG SMP |
| Phenylalanine, tyrosine and tryptophan biosynthesis | 3 over 4 | 6.94E-07 | 6.1585 | 2.36E-05 | 1.90E-06 | 1 | KEGG SMP |
| Valine, leucine and isoleucine degradation | 2 over 40 | 9.67E-07 | 6.0145 | 3.19E-05 | 2.33E-06 | 0.01084 | KEGG SMP |
| Arginine and proline metabolism | 7 over 38 | 9.80E-07 | 6.0088 | 3.19E-05 | 2.33E-06 | 0.48632 | KEGG SMP |
| Ubiquinone and other terpenoid-quinone biosynthesis | 1 over 9 | 1.03E-06 | 5.9886 | 3.19E-05 | 2.33E-06 | 0 | KEGG SMP |
| Histidine metabolism | 7 over 16 | 1.03E-06 | 5.9875 | 3.19E-05 | 2.33E-06 | 0.67212 | KEGG SMP |
| Nitrogen metabolism | 2 over 6 | 1.30E-06 | 5.886 | 3.77E-05 | 2.82E-06 | 0 | KEGG SMP |
| D-Glutamine and D-glutamate metabolism | 3 over 6 | 1.50E-06 | 5.823 | 4.21E-05 | 3.13E-06 | 0.5 | KEGG SMP |
| Glycine, serine and threonine metabolism | 5 over 33 | 8.23E-06 | 5.0846 | 2.22E-04 | 1.65E-05 | 0.14714 | KEGG SMP |
| Purine metabolism | 6 over 65 | 1.77E-05 | 4.7524 | 4.60E-04 | 3.41E-05 | 0.13659 | KEGG SMP |
| Cysteine and methionine metabolism | 5 over 33 | 2.71E-05 | 4.5671 | 6.77E-04 | 5.02E-05 | 0.27018 | KEGG SMP SMP |
| Glyoxylate and dicarboxylate metabolism | 3 over 32 | 2.80E-05 | 4.5533 | 6.77E-04 | 5.02E-05 | 0.02381 | KEGG |
| Drug metabolism - cytochrome P450 | 1 over 55 | 4.60E-05 | 4.337 | 0.0010587 | 7.98E-05 | 0 | KEGG |
| Steroid biosynthesis | 2 over 42 | 5.18E-05 | 4.2854 | 0.0011402 | 8.69E-05 | 0.0392 | KEGG |
| Citrate cycle (TCA cycle) | 2 over 20 | 4.59E-04 | 3.3379 | 0.009646 | 7.46E-04 | 0.10859 | KEGG SMP |
| Steroid hormone biosynthesis | 7 over 85 | 8.42E-04 | 3.0745 | 0.016849 | 0.0013266 | 0.17438 | KEGG SMP |
| Porphyrin and chlorophyll metabolism | 5 over 30 | 8.67E-04 | 3.0618 | 0.016849 | 0.0013266 | 0.12753 | KEGG |
| Tryptophan metabolism | 9 over 41 | 9.54E-04 | 3.0202 | 0.017181 | 0.0014181 | 0.31659 | KEGG SMP |
| beta-Alanine metabolism | 5 over 21 | 0.0014398 | 2.8417 | 0.024477 | 0.0020797 | 0.60821 | KEGG SMP |
| Pantothenate and CoA biosynthesis | 4 over 19 | 0.0015128 | 2.8202 | 0.024477 | 0.0021261 | 0.04286 | KEGG SMP |
| Selenocompound metabolism | 2 over 20 | 0.0051433 | 2.2888 | 0.077149 | 0.0070382 | 0.22727 | KEGG SMP |
| Propanoate metabolism | 1 over 23 | 0.009814 | 2.0082 | 0.1374 | 0.013085 | 0 | KEGG SMP |
| Terpenoid backbone biosynthesis | 2 over 18 | 0.011213 | 1.9503 | 0.14577 | 0.014577 | 0.11429 | KEGG |
| Glycosylphosphatidylinositol (GPI)-anchor biosynthesis | 1 over 14 | 0.013719 | 1.8627 | 0.16463 | 0.0174 | 0.00399 | KEGG |
| Pyruvate metabolism | 1 over 22 | 0.014449 | 1.8402 | 0.16463 | 0.017889 | 0 | KEGG SMP |
| Pyrimidine metabolism | 6 over 39 | 0.01507 | 1.8219 | 0.16463 | 0.018224 | 0.15301 | KEGG SMP |
| Ether lipid metabolism | 1 over 20 | 0.022725 | 1.6435 | 0.20452 | 0.026856 | 0 | KEGG |
| Glutathione metabolism | 3 over 28 | 0.03564 | 1.4481 | 0.28512 | 0.041184 | 0.02675 | KEGG SMP |

## Slide 52
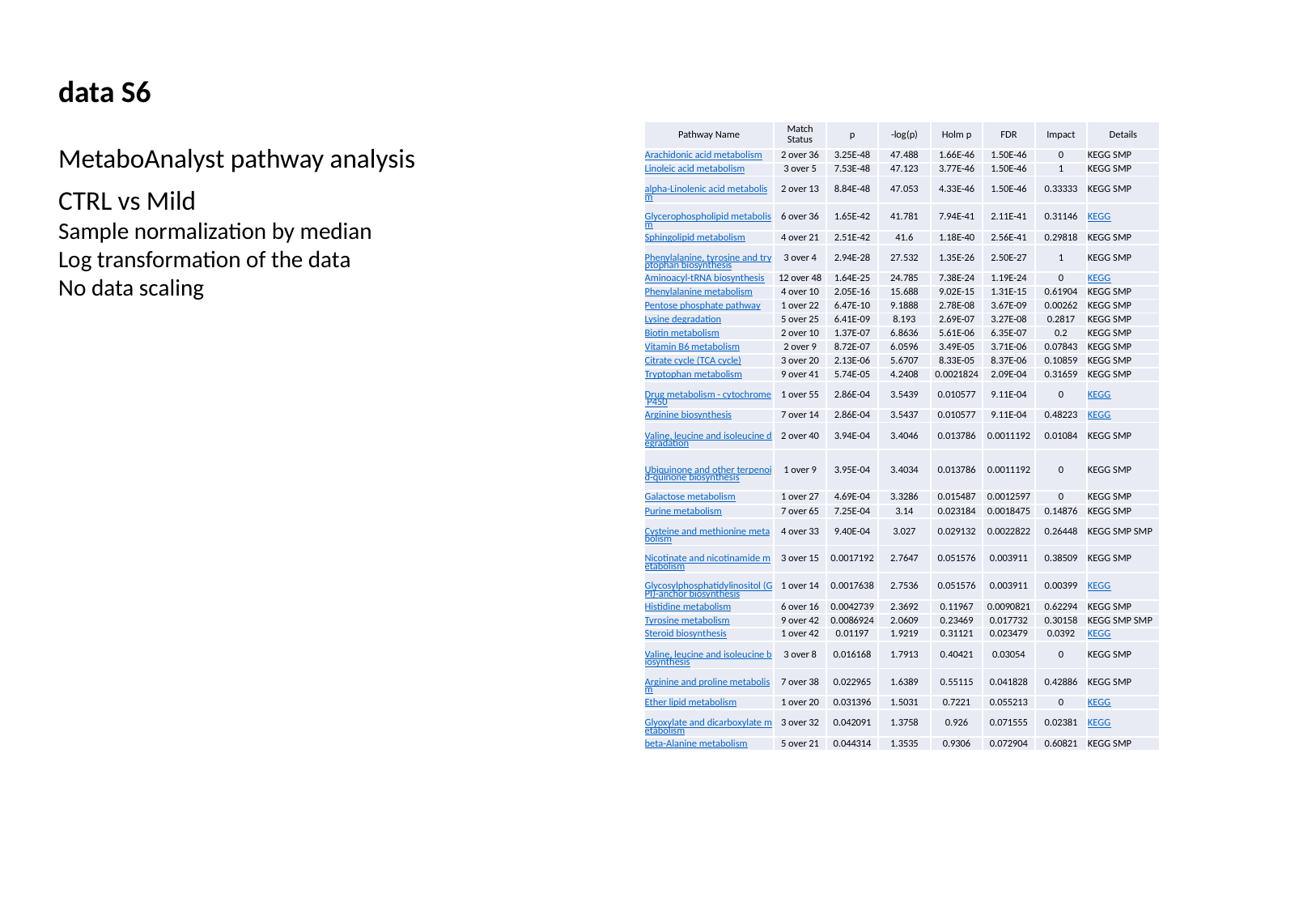

data S6
MetaboAnalyst pathway analysis
CTRL vs Mild
Sample normalization by median
Log transformation of the data
No data scaling
| Pathway Name | Match Status | p | -log(p) | Holm p | FDR | Impact | Details |
| --- | --- | --- | --- | --- | --- | --- | --- |
| Arachidonic acid metabolism | 2 over 36 | 3.25E-48 | 47.488 | 1.66E-46 | 1.50E-46 | 0 | KEGG SMP |
| Linoleic acid metabolism | 3 over 5 | 7.53E-48 | 47.123 | 3.77E-46 | 1.50E-46 | 1 | KEGG SMP |
| alpha-Linolenic acid metabolism | 2 over 13 | 8.84E-48 | 47.053 | 4.33E-46 | 1.50E-46 | 0.33333 | KEGG SMP |
| Glycerophospholipid metabolism | 6 over 36 | 1.65E-42 | 41.781 | 7.94E-41 | 2.11E-41 | 0.31146 | KEGG |
| Sphingolipid metabolism | 4 over 21 | 2.51E-42 | 41.6 | 1.18E-40 | 2.56E-41 | 0.29818 | KEGG SMP |
| Phenylalanine, tyrosine and tryptophan biosynthesis | 3 over 4 | 2.94E-28 | 27.532 | 1.35E-26 | 2.50E-27 | 1 | KEGG SMP |
| Aminoacyl-tRNA biosynthesis | 12 over 48 | 1.64E-25 | 24.785 | 7.38E-24 | 1.19E-24 | 0 | KEGG |
| Phenylalanine metabolism | 4 over 10 | 2.05E-16 | 15.688 | 9.02E-15 | 1.31E-15 | 0.61904 | KEGG SMP |
| Pentose phosphate pathway | 1 over 22 | 6.47E-10 | 9.1888 | 2.78E-08 | 3.67E-09 | 0.00262 | KEGG SMP |
| Lysine degradation | 5 over 25 | 6.41E-09 | 8.193 | 2.69E-07 | 3.27E-08 | 0.2817 | KEGG SMP |
| Biotin metabolism | 2 over 10 | 1.37E-07 | 6.8636 | 5.61E-06 | 6.35E-07 | 0.2 | KEGG SMP |
| Vitamin B6 metabolism | 2 over 9 | 8.72E-07 | 6.0596 | 3.49E-05 | 3.71E-06 | 0.07843 | KEGG SMP |
| Citrate cycle (TCA cycle) | 3 over 20 | 2.13E-06 | 5.6707 | 8.33E-05 | 8.37E-06 | 0.10859 | KEGG SMP |
| Tryptophan metabolism | 9 over 41 | 5.74E-05 | 4.2408 | 0.0021824 | 2.09E-04 | 0.31659 | KEGG SMP |
| Drug metabolism - cytochrome P450 | 1 over 55 | 2.86E-04 | 3.5439 | 0.010577 | 9.11E-04 | 0 | KEGG |
| Arginine biosynthesis | 7 over 14 | 2.86E-04 | 3.5437 | 0.010577 | 9.11E-04 | 0.48223 | KEGG |
| Valine, leucine and isoleucine degradation | 2 over 40 | 3.94E-04 | 3.4046 | 0.013786 | 0.0011192 | 0.01084 | KEGG SMP |
| Ubiquinone and other terpenoid-quinone biosynthesis | 1 over 9 | 3.95E-04 | 3.4034 | 0.013786 | 0.0011192 | 0 | KEGG SMP |
| Galactose metabolism | 1 over 27 | 4.69E-04 | 3.3286 | 0.015487 | 0.0012597 | 0 | KEGG SMP |
| Purine metabolism | 7 over 65 | 7.25E-04 | 3.14 | 0.023184 | 0.0018475 | 0.14876 | KEGG SMP |
| Cysteine and methionine metabolism | 4 over 33 | 9.40E-04 | 3.027 | 0.029132 | 0.0022822 | 0.26448 | KEGG SMP SMP |
| Nicotinate and nicotinamide metabolism | 3 over 15 | 0.0017192 | 2.7647 | 0.051576 | 0.003911 | 0.38509 | KEGG SMP |
| Glycosylphosphatidylinositol (GPI)-anchor biosynthesis | 1 over 14 | 0.0017638 | 2.7536 | 0.051576 | 0.003911 | 0.00399 | KEGG |
| Histidine metabolism | 6 over 16 | 0.0042739 | 2.3692 | 0.11967 | 0.0090821 | 0.62294 | KEGG SMP |
| Tyrosine metabolism | 9 over 42 | 0.0086924 | 2.0609 | 0.23469 | 0.017732 | 0.30158 | KEGG SMP SMP |
| Steroid biosynthesis | 1 over 42 | 0.01197 | 1.9219 | 0.31121 | 0.023479 | 0.0392 | KEGG |
| Valine, leucine and isoleucine biosynthesis | 3 over 8 | 0.016168 | 1.7913 | 0.40421 | 0.03054 | 0 | KEGG SMP |
| Arginine and proline metabolism | 7 over 38 | 0.022965 | 1.6389 | 0.55115 | 0.041828 | 0.42886 | KEGG SMP |
| Ether lipid metabolism | 1 over 20 | 0.031396 | 1.5031 | 0.7221 | 0.055213 | 0 | KEGG |
| Glyoxylate and dicarboxylate metabolism | 3 over 32 | 0.042091 | 1.3758 | 0.926 | 0.071555 | 0.02381 | KEGG |
| beta-Alanine metabolism | 5 over 21 | 0.044314 | 1.3535 | 0.9306 | 0.072904 | 0.60821 | KEGG SMP |

## Slide 53
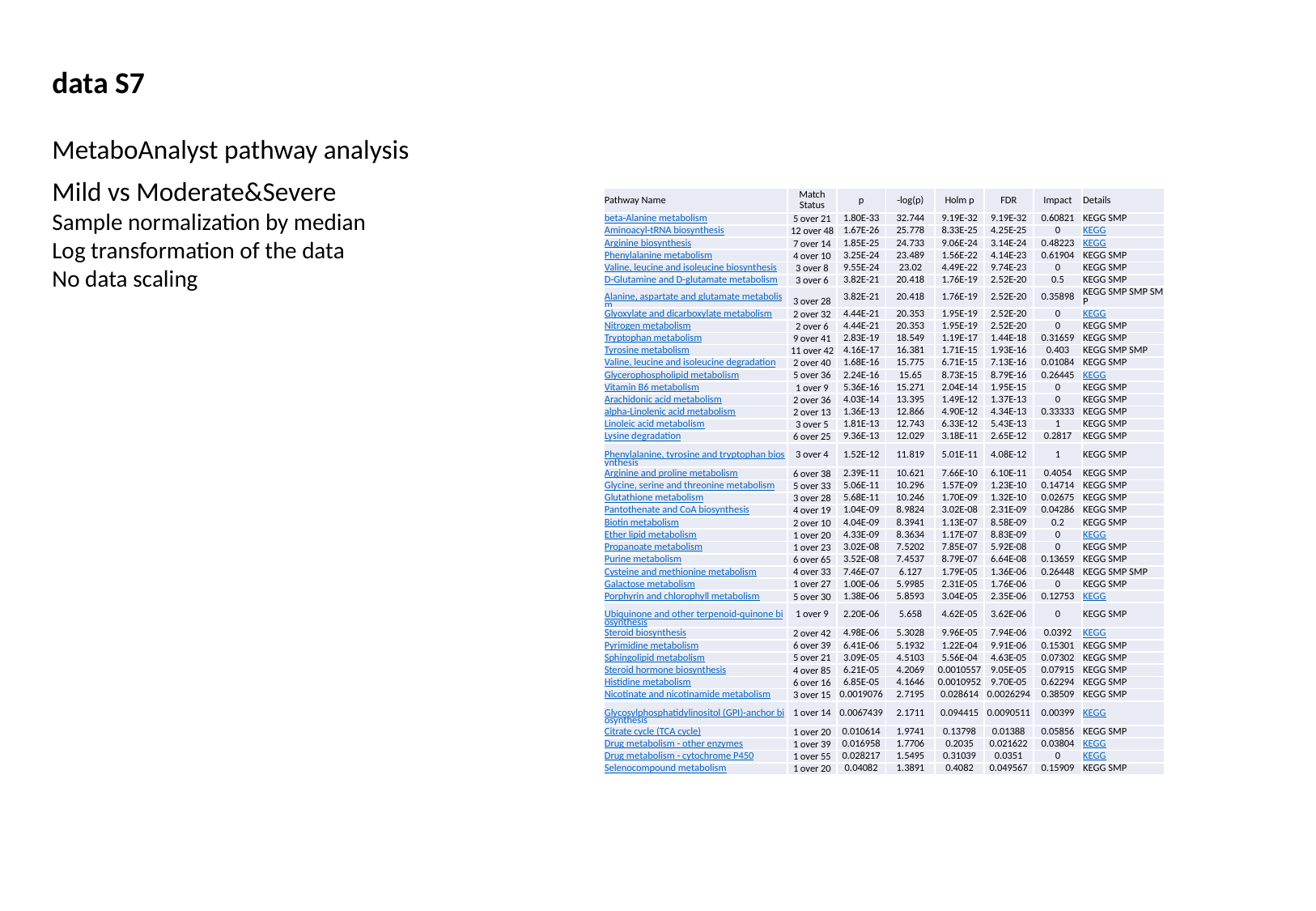

data S7
MetaboAnalyst pathway analysis
Mild vs Moderate&Severe
Sample normalization by median
Log transformation of the data
No data scaling
| Pathway Name | Match Status | p | -log(p) | Holm p | FDR | Impact | Details |
| --- | --- | --- | --- | --- | --- | --- | --- |
| beta-Alanine metabolism | 5 over 21 | 1.80E-33 | 32.744 | 9.19E-32 | 9.19E-32 | 0.60821 | KEGG SMP |
| Aminoacyl-tRNA biosynthesis | 12 over 48 | 1.67E-26 | 25.778 | 8.33E-25 | 4.25E-25 | 0 | KEGG |
| Arginine biosynthesis | 7 over 14 | 1.85E-25 | 24.733 | 9.06E-24 | 3.14E-24 | 0.48223 | KEGG |
| Phenylalanine metabolism | 4 over 10 | 3.25E-24 | 23.489 | 1.56E-22 | 4.14E-23 | 0.61904 | KEGG SMP |
| Valine, leucine and isoleucine biosynthesis | 3 over 8 | 9.55E-24 | 23.02 | 4.49E-22 | 9.74E-23 | 0 | KEGG SMP |
| D-Glutamine and D-glutamate metabolism | 3 over 6 | 3.82E-21 | 20.418 | 1.76E-19 | 2.52E-20 | 0.5 | KEGG SMP |
| Alanine, aspartate and glutamate metabolism | 3 over 28 | 3.82E-21 | 20.418 | 1.76E-19 | 2.52E-20 | 0.35898 | KEGG SMP SMP SMP |
| Glyoxylate and dicarboxylate metabolism | 2 over 32 | 4.44E-21 | 20.353 | 1.95E-19 | 2.52E-20 | 0 | KEGG |
| Nitrogen metabolism | 2 over 6 | 4.44E-21 | 20.353 | 1.95E-19 | 2.52E-20 | 0 | KEGG SMP |
| Tryptophan metabolism | 9 over 41 | 2.83E-19 | 18.549 | 1.19E-17 | 1.44E-18 | 0.31659 | KEGG SMP |
| Tyrosine metabolism | 11 over 42 | 4.16E-17 | 16.381 | 1.71E-15 | 1.93E-16 | 0.403 | KEGG SMP SMP |
| Valine, leucine and isoleucine degradation | 2 over 40 | 1.68E-16 | 15.775 | 6.71E-15 | 7.13E-16 | 0.01084 | KEGG SMP |
| Glycerophospholipid metabolism | 5 over 36 | 2.24E-16 | 15.65 | 8.73E-15 | 8.79E-16 | 0.26445 | KEGG |
| Vitamin B6 metabolism | 1 over 9 | 5.36E-16 | 15.271 | 2.04E-14 | 1.95E-15 | 0 | KEGG SMP |
| Arachidonic acid metabolism | 2 over 36 | 4.03E-14 | 13.395 | 1.49E-12 | 1.37E-13 | 0 | KEGG SMP |
| alpha-Linolenic acid metabolism | 2 over 13 | 1.36E-13 | 12.866 | 4.90E-12 | 4.34E-13 | 0.33333 | KEGG SMP |
| Linoleic acid metabolism | 3 over 5 | 1.81E-13 | 12.743 | 6.33E-12 | 5.43E-13 | 1 | KEGG SMP |
| Lysine degradation | 6 over 25 | 9.36E-13 | 12.029 | 3.18E-11 | 2.65E-12 | 0.2817 | KEGG SMP |
| Phenylalanine, tyrosine and tryptophan biosynthesis | 3 over 4 | 1.52E-12 | 11.819 | 5.01E-11 | 4.08E-12 | 1 | KEGG SMP |
| Arginine and proline metabolism | 6 over 38 | 2.39E-11 | 10.621 | 7.66E-10 | 6.10E-11 | 0.4054 | KEGG SMP |
| Glycine, serine and threonine metabolism | 5 over 33 | 5.06E-11 | 10.296 | 1.57E-09 | 1.23E-10 | 0.14714 | KEGG SMP |
| Glutathione metabolism | 3 over 28 | 5.68E-11 | 10.246 | 1.70E-09 | 1.32E-10 | 0.02675 | KEGG SMP |
| Pantothenate and CoA biosynthesis | 4 over 19 | 1.04E-09 | 8.9824 | 3.02E-08 | 2.31E-09 | 0.04286 | KEGG SMP |
| Biotin metabolism | 2 over 10 | 4.04E-09 | 8.3941 | 1.13E-07 | 8.58E-09 | 0.2 | KEGG SMP |
| Ether lipid metabolism | 1 over 20 | 4.33E-09 | 8.3634 | 1.17E-07 | 8.83E-09 | 0 | KEGG |
| Propanoate metabolism | 1 over 23 | 3.02E-08 | 7.5202 | 7.85E-07 | 5.92E-08 | 0 | KEGG SMP |
| Purine metabolism | 6 over 65 | 3.52E-08 | 7.4537 | 8.79E-07 | 6.64E-08 | 0.13659 | KEGG SMP |
| Cysteine and methionine metabolism | 4 over 33 | 7.46E-07 | 6.127 | 1.79E-05 | 1.36E-06 | 0.26448 | KEGG SMP SMP |
| Galactose metabolism | 1 over 27 | 1.00E-06 | 5.9985 | 2.31E-05 | 1.76E-06 | 0 | KEGG SMP |
| Porphyrin and chlorophyll metabolism | 5 over 30 | 1.38E-06 | 5.8593 | 3.04E-05 | 2.35E-06 | 0.12753 | KEGG |
| Ubiquinone and other terpenoid-quinone biosynthesis | 1 over 9 | 2.20E-06 | 5.658 | 4.62E-05 | 3.62E-06 | 0 | KEGG SMP |
| Steroid biosynthesis | 2 over 42 | 4.98E-06 | 5.3028 | 9.96E-05 | 7.94E-06 | 0.0392 | KEGG |
| Pyrimidine metabolism | 6 over 39 | 6.41E-06 | 5.1932 | 1.22E-04 | 9.91E-06 | 0.15301 | KEGG SMP |
| Sphingolipid metabolism | 5 over 21 | 3.09E-05 | 4.5103 | 5.56E-04 | 4.63E-05 | 0.07302 | KEGG SMP |
| Steroid hormone biosynthesis | 4 over 85 | 6.21E-05 | 4.2069 | 0.0010557 | 9.05E-05 | 0.07915 | KEGG SMP |
| Histidine metabolism | 6 over 16 | 6.85E-05 | 4.1646 | 0.0010952 | 9.70E-05 | 0.62294 | KEGG SMP |
| Nicotinate and nicotinamide metabolism | 3 over 15 | 0.0019076 | 2.7195 | 0.028614 | 0.0026294 | 0.38509 | KEGG SMP |
| Glycosylphosphatidylinositol (GPI)-anchor biosynthesis | 1 over 14 | 0.0067439 | 2.1711 | 0.094415 | 0.0090511 | 0.00399 | KEGG |
| Citrate cycle (TCA cycle) | 1 over 20 | 0.010614 | 1.9741 | 0.13798 | 0.01388 | 0.05856 | KEGG SMP |
| Drug metabolism - other enzymes | 1 over 39 | 0.016958 | 1.7706 | 0.2035 | 0.021622 | 0.03804 | KEGG |
| Drug metabolism - cytochrome P450 | 1 over 55 | 0.028217 | 1.5495 | 0.31039 | 0.0351 | 0 | KEGG |
| Selenocompound metabolism | 1 over 20 | 0.04082 | 1.3891 | 0.4082 | 0.049567 | 0.15909 | KEGG SMP |

## Slide 54
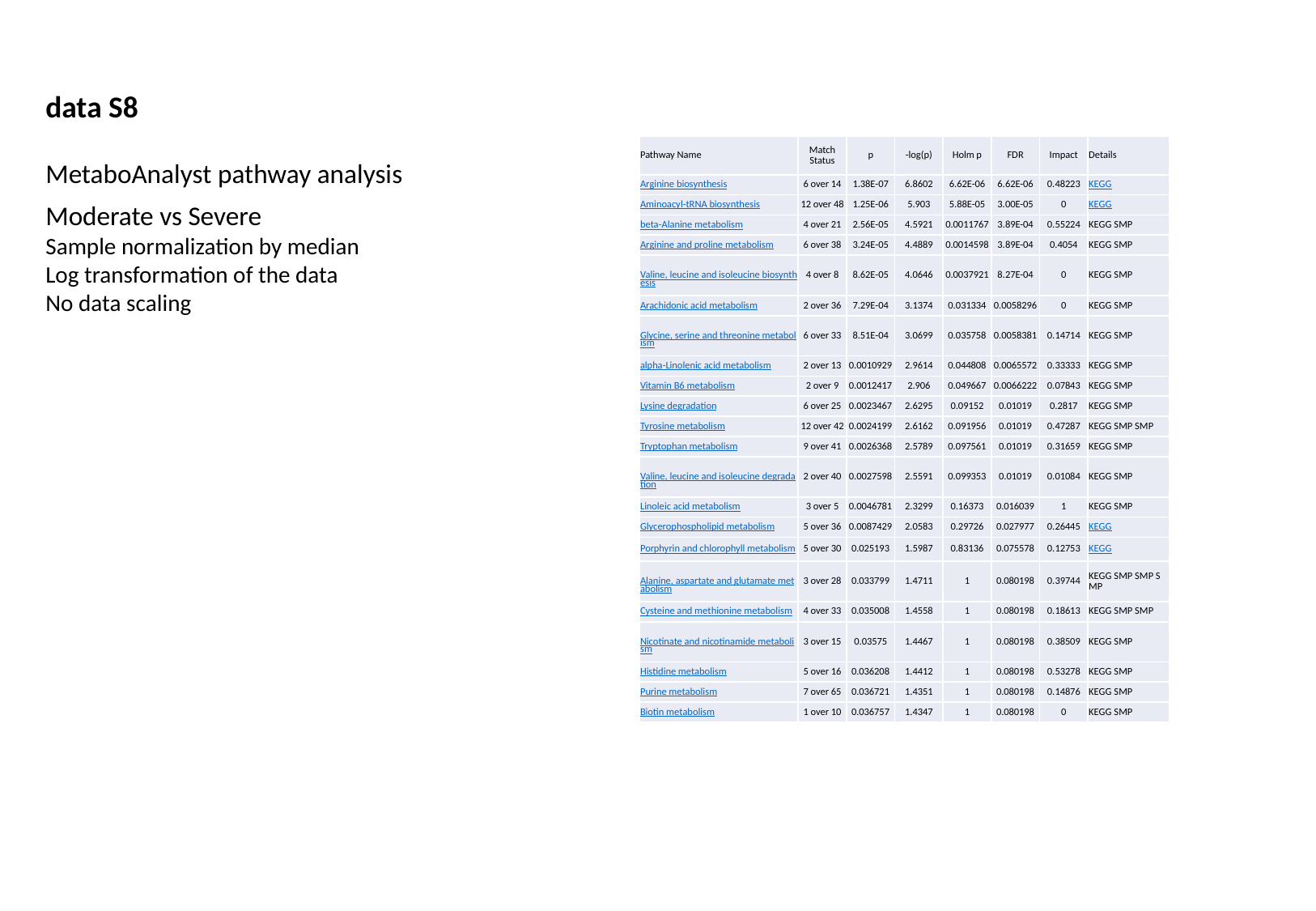

data S8
MetaboAnalyst pathway analysis
Moderate vs Severe
Sample normalization by median
Log transformation of the data
No data scaling
| Pathway Name | Match Status | p | -log(p) | Holm p | FDR | Impact | Details |
| --- | --- | --- | --- | --- | --- | --- | --- |
| Arginine biosynthesis | 6 over 14 | 1.38E-07 | 6.8602 | 6.62E-06 | 6.62E-06 | 0.48223 | KEGG |
| Aminoacyl-tRNA biosynthesis | 12 over 48 | 1.25E-06 | 5.903 | 5.88E-05 | 3.00E-05 | 0 | KEGG |
| beta-Alanine metabolism | 4 over 21 | 2.56E-05 | 4.5921 | 0.0011767 | 3.89E-04 | 0.55224 | KEGG SMP |
| Arginine and proline metabolism | 6 over 38 | 3.24E-05 | 4.4889 | 0.0014598 | 3.89E-04 | 0.4054 | KEGG SMP |
| Valine, leucine and isoleucine biosynthesis | 4 over 8 | 8.62E-05 | 4.0646 | 0.0037921 | 8.27E-04 | 0 | KEGG SMP |
| Arachidonic acid metabolism | 2 over 36 | 7.29E-04 | 3.1374 | 0.031334 | 0.0058296 | 0 | KEGG SMP |
| Glycine, serine and threonine metabolism | 6 over 33 | 8.51E-04 | 3.0699 | 0.035758 | 0.0058381 | 0.14714 | KEGG SMP |
| alpha-Linolenic acid metabolism | 2 over 13 | 0.0010929 | 2.9614 | 0.044808 | 0.0065572 | 0.33333 | KEGG SMP |
| Vitamin B6 metabolism | 2 over 9 | 0.0012417 | 2.906 | 0.049667 | 0.0066222 | 0.07843 | KEGG SMP |
| Lysine degradation | 6 over 25 | 0.0023467 | 2.6295 | 0.09152 | 0.01019 | 0.2817 | KEGG SMP |
| Tyrosine metabolism | 12 over 42 | 0.0024199 | 2.6162 | 0.091956 | 0.01019 | 0.47287 | KEGG SMP SMP |
| Tryptophan metabolism | 9 over 41 | 0.0026368 | 2.5789 | 0.097561 | 0.01019 | 0.31659 | KEGG SMP |
| Valine, leucine and isoleucine degradation | 2 over 40 | 0.0027598 | 2.5591 | 0.099353 | 0.01019 | 0.01084 | KEGG SMP |
| Linoleic acid metabolism | 3 over 5 | 0.0046781 | 2.3299 | 0.16373 | 0.016039 | 1 | KEGG SMP |
| Glycerophospholipid metabolism | 5 over 36 | 0.0087429 | 2.0583 | 0.29726 | 0.027977 | 0.26445 | KEGG |
| Porphyrin and chlorophyll metabolism | 5 over 30 | 0.025193 | 1.5987 | 0.83136 | 0.075578 | 0.12753 | KEGG |
| Alanine, aspartate and glutamate metabolism | 3 over 28 | 0.033799 | 1.4711 | 1 | 0.080198 | 0.39744 | KEGG SMP SMP SMP |
| Cysteine and methionine metabolism | 4 over 33 | 0.035008 | 1.4558 | 1 | 0.080198 | 0.18613 | KEGG SMP SMP |
| Nicotinate and nicotinamide metabolism | 3 over 15 | 0.03575 | 1.4467 | 1 | 0.080198 | 0.38509 | KEGG SMP |
| Histidine metabolism | 5 over 16 | 0.036208 | 1.4412 | 1 | 0.080198 | 0.53278 | KEGG SMP |
| Purine metabolism | 7 over 65 | 0.036721 | 1.4351 | 1 | 0.080198 | 0.14876 | KEGG SMP |
| Biotin metabolism | 1 over 10 | 0.036757 | 1.4347 | 1 | 0.080198 | 0 | KEGG SMP |

## Slide 55
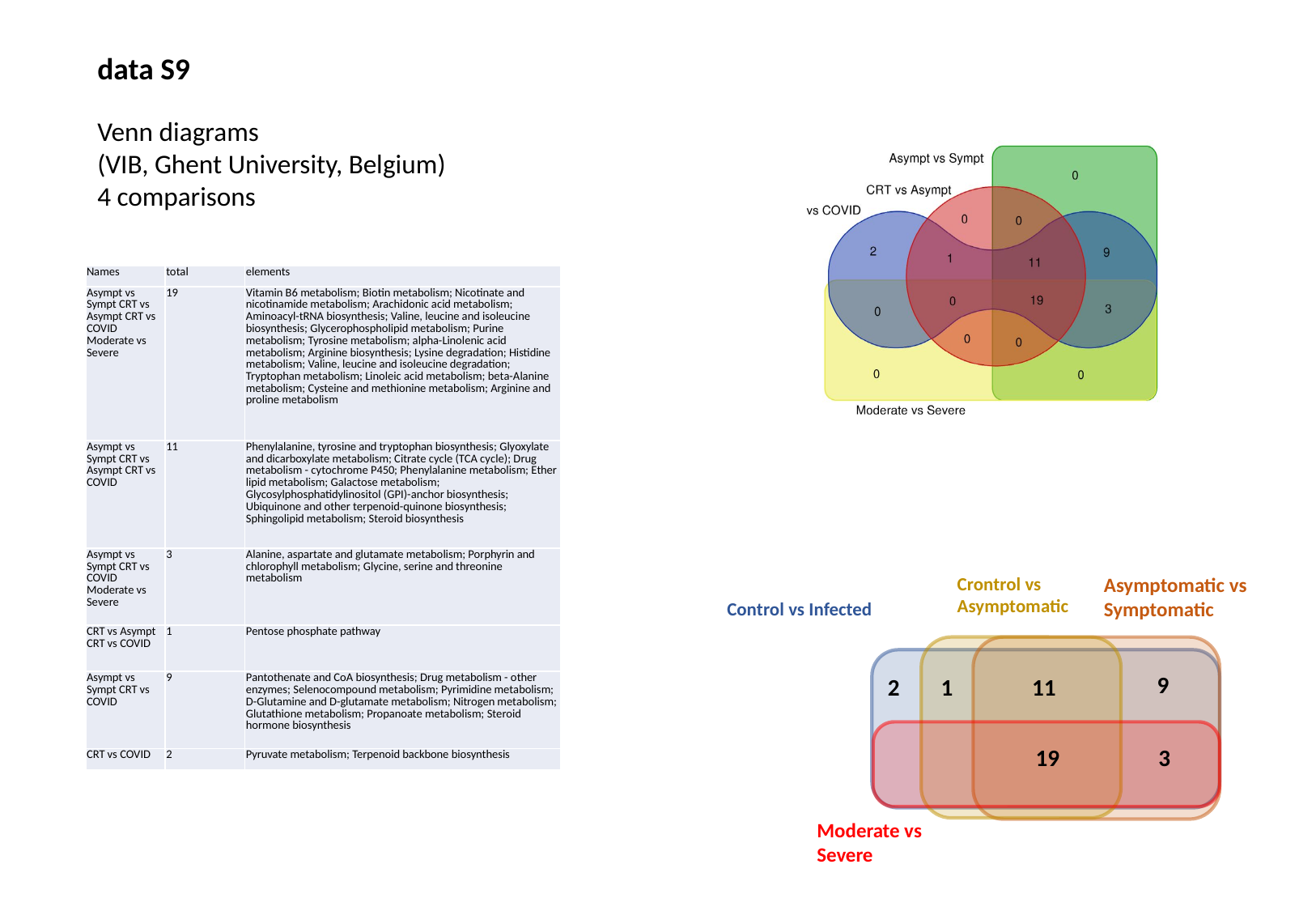

data S9
Venn diagrams
(VIB, Ghent University, Belgium)
4 comparisons
| Names | total | elements |
| --- | --- | --- |
| Asympt vs Sympt CRT vs Asympt CRT vs COVID Moderate vs Severe | 19 | Vitamin B6 metabolism; Biotin metabolism; Nicotinate and nicotinamide metabolism; Arachidonic acid metabolism; Aminoacyl-tRNA biosynthesis; Valine, leucine and isoleucine biosynthesis; Glycerophospholipid metabolism; Purine metabolism; Tyrosine metabolism; alpha-Linolenic acid metabolism; Arginine biosynthesis; Lysine degradation; Histidine metabolism; Valine, leucine and isoleucine degradation; Tryptophan metabolism; Linoleic acid metabolism; beta-Alanine metabolism; Cysteine and methionine metabolism; Arginine and proline metabolism |
| Asympt vs Sympt CRT vs Asympt CRT vs COVID | 11 | Phenylalanine, tyrosine and tryptophan biosynthesis; Glyoxylate and dicarboxylate metabolism; Citrate cycle (TCA cycle); Drug metabolism - cytochrome P450; Phenylalanine metabolism; Ether lipid metabolism; Galactose metabolism; Glycosylphosphatidylinositol (GPI)-anchor biosynthesis; Ubiquinone and other terpenoid-quinone biosynthesis; Sphingolipid metabolism; Steroid biosynthesis |
| Asympt vs Sympt CRT vs COVID Moderate vs Severe | 3 | Alanine, aspartate and glutamate metabolism; Porphyrin and chlorophyll metabolism; Glycine, serine and threonine metabolism |
| CRT vs Asympt CRT vs COVID | 1 | Pentose phosphate pathway |
| Asympt vs Sympt CRT vs COVID | 9 | Pantothenate and CoA biosynthesis; Drug metabolism - other enzymes; Selenocompound metabolism; Pyrimidine metabolism; D-Glutamine and D-glutamate metabolism; Nitrogen metabolism; Glutathione metabolism; Propanoate metabolism; Steroid hormone biosynthesis |
| CRT vs COVID | 2 | Pyruvate metabolism; Terpenoid backbone biosynthesis |
Crontrol vs Asymptomatic
Asymptomatic vs Symptomatic
Control vs Infected
9
2
1
11
19
3
Moderate vs Severe

## Slide 56
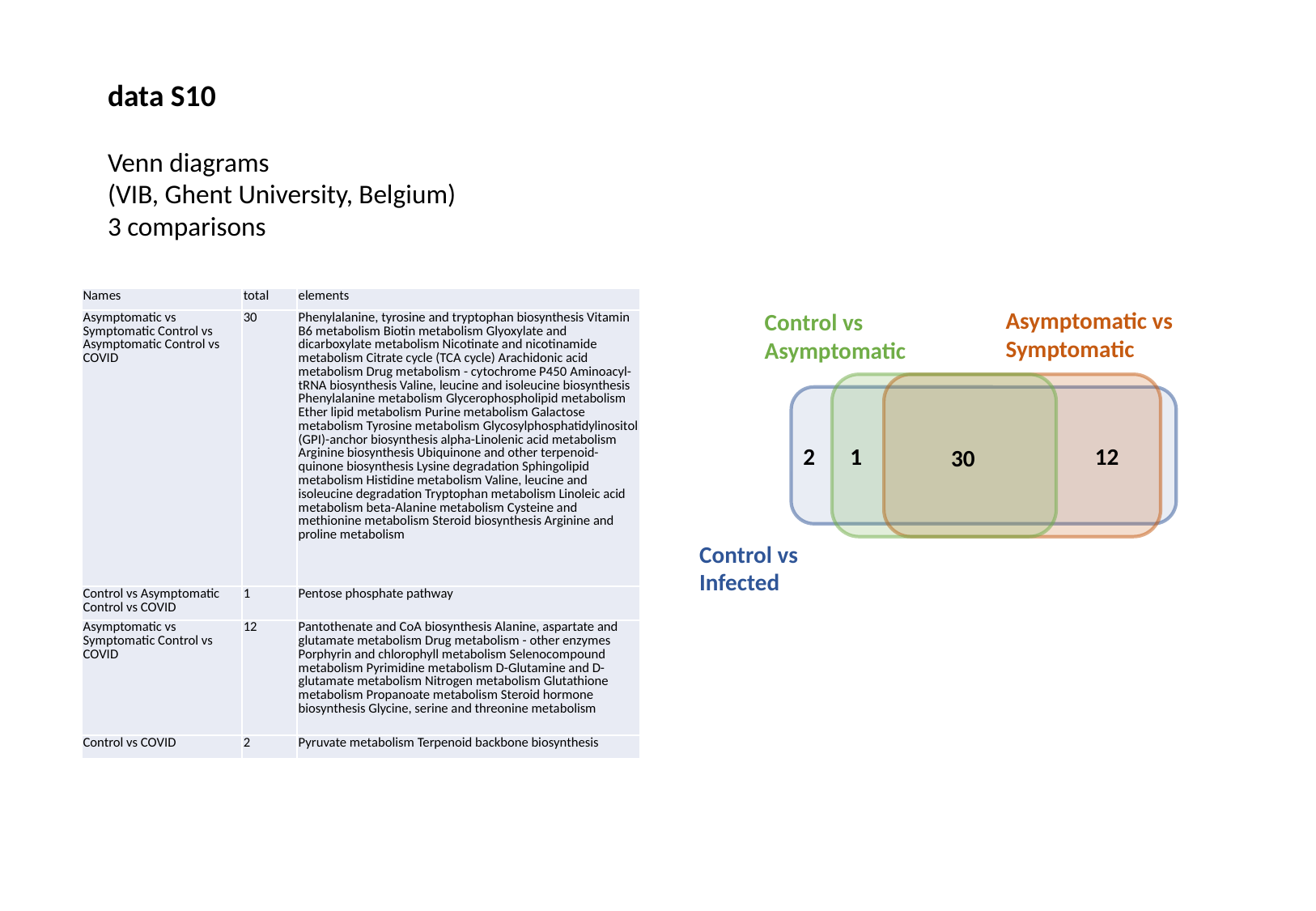

data S10
Venn diagrams
(VIB, Ghent University, Belgium)
3 comparisons
| Names | total | elements |
| --- | --- | --- |
| Asymptomatic vs Symptomatic Control vs Asymptomatic Control vs COVID | 30 | Phenylalanine, tyrosine and tryptophan biosynthesis Vitamin B6 metabolism Biotin metabolism Glyoxylate and dicarboxylate metabolism Nicotinate and nicotinamide metabolism Citrate cycle (TCA cycle) Arachidonic acid metabolism Drug metabolism - cytochrome P450 Aminoacyl-tRNA biosynthesis Valine, leucine and isoleucine biosynthesis Phenylalanine metabolism Glycerophospholipid metabolism Ether lipid metabolism Purine metabolism Galactose metabolism Tyrosine metabolism Glycosylphosphatidylinositol (GPI)-anchor biosynthesis alpha-Linolenic acid metabolism Arginine biosynthesis Ubiquinone and other terpenoid-quinone biosynthesis Lysine degradation Sphingolipid metabolism Histidine metabolism Valine, leucine and isoleucine degradation Tryptophan metabolism Linoleic acid metabolism beta-Alanine metabolism Cysteine and methionine metabolism Steroid biosynthesis Arginine and proline metabolism |
| Control vs Asymptomatic Control vs COVID | 1 | Pentose phosphate pathway |
| Asymptomatic vs Symptomatic Control vs COVID | 12 | Pantothenate and CoA biosynthesis Alanine, aspartate and glutamate metabolism Drug metabolism - other enzymes Porphyrin and chlorophyll metabolism Selenocompound metabolism Pyrimidine metabolism D-Glutamine and D-glutamate metabolism Nitrogen metabolism Glutathione metabolism Propanoate metabolism Steroid hormone biosynthesis Glycine, serine and threonine metabolism |
| Control vs COVID | 2 | Pyruvate metabolism Terpenoid backbone biosynthesis |
Asymptomatic vs Symptomatic
Control vs Asymptomatic
1
12
2
30
Control vs Infected
